# Supplementary material for: Analysis of differences and commonalities in wildlife hunting across the Africa-Europe South-North gradient
Source: PLoS Biol. 2022 Aug 30;20(8):e3001707. doi: 10.1371/journal.pbio.3001707 (PMC9426919; doi:10.1371/journal.pbio.3001707)
Supplement: S1 Appendix — (DOCX) [file pbio.3001707.s001.docx]

**Supplementary Online Material (SOM)**

1. **Data collection**

*Table A The list of PAs and their IUCN management category*

| **ID** | **WDPA ID** | **PA** | **Country** | **Continent** | **IUCN management** |
| --- | --- | --- | --- | --- | --- |
| 1 | 169110 | Donau-Auen National Park | Austria | Europe | II |
| 2 | 174775 | Gesäuse National Park | Austria | Europe | II |
| 3 | 169111 | Thayatal National Park | Austria | Europe | V |
| 4 | 555547993 | Mbam et Djerem National Park | Cameroon | Africa | II |
| 5 | 3011 | Bénoué National Park | Cameroon | Africa | II |
| 6 | 308624 | Boumba Bek National Park | Cameroon | Africa | II |
| 7 | 1245 | Lobéké National Park | Cameroon | Africa | II |
| 8 | 3012 | Dja Biosphere Reserve | Cameroon | Africa | UNESCO Biosphere Reserve |
| 9 | 1242 | Campo Ma'an National Park | Cameroon | Africa | II |
| 10 | 606 | Bouba Ndjida National Park | Cameroon | Africa | II |
| 11 | 555547994 | Mt. Cameroon National Park | Cameroon | Africa | II |
| 12 | 555547998 | Bakossi National Park | Cameroon | Africa | II |
| 13 | 31459 | Dzanga-Sangha Complex of Protected Areas | Central African Republic | Africa | not reported |
| 14 | 28745 | Mbaéré-Bodingué National Park | Central African Republic | Africa | VI |
| 15 | 641 | Zakouma National Park | Chad | Africa | II |
| 16 | 555558302 | Sena Oura National Park | Chad | Africa | II |
| 17 | 642 | Manda National Park | Chad | Africa | II |
| 18 | 5168 | Binder-Léré Faunal Reserve | Chad | Africa | IV |
| 19 | 643 | Odzala-Kokoua National Park | Congo Republic | Africa | II |
| 20 | 13694 | Dimonika Biosphere Reserve | Congo Republic | Africa | UNESCO Biosphere Reserve |
| 21 | 2266 | Lefini faunal reserve | Congo Republic | Africa | IV |
| 22 | 344525 | Bohemian Switzerland National Park | Czech Republic | Europe | II |
| 23 | 344526 | Podyjí National Park | Czech Republic | Europe | II |
| 24 | 344527 | Šumava National Park | Czech Republic | Europe | II |
| 25 | 344524 | Krkonoše National Park | Czech Republic | Europe | V |
| 26 | 2277 | Mago National Park | Ethiopia | Africa | II |
| 27 | 2281 | Bale Mountains National Park | Ethiopia | Africa | II |
| 28 | 2278 | Nechsar National Park | Ethiopia | Africa | II |
| 29 | 13704 | Gambella National Park | Ethiopia | Africa | II |
| 30 | 83231 | Écrins National Park | France | Europe | V |
| 31 | 103150 | Vanoise National Park | France | Europe | V |
| 32 | 103151 | Pyrénées National Park | France | Europe | V |
| 33 | 660 | Cévennes National Park | France | Europe | V |
| 34 | 303880 | Waka National Park | Gabon | Africa | II |
| 35 | 303877 | Moukalaba-Doudou National Park | Gabon | Africa | II |
| 36 | 301850 | Mayumba National Park | Gabon | Africa | II |
| 37 | 303873 | Ivindo National Park | Gabon | Africa | II |
| 38 | 102224 | Nationalpark Lower Odra Valley | Germany | Europe | II |
| 39 | 667 | Bavarian Forest National Park | Germany | Europe | II |
| 40 | 148534 | Hainich National Park | Germany | Europe | II |
| 41 | 32666 | Saxon Switzerland National Park | Germany | Europe | II |
| 42 | 670 | Digya National Park | Ghana | Africa | II |
| 43 | 5173 | Ankasa National Park | Ghana | Africa | II |
| 44 | 672 | Bia National Park | Ghana | Africa | II |
| 45 | 26460 | Kakum National Park | Ghana | Africa | II |
| 46 | 671 | Bui National Park | Ghana | Africa | II |
| 47 | 29421 | National Park of the Upper Niger | Guinea | Africa | II |
| 48 | 2574 | Mount Nimba Strict Nature Reserve | Guinea | Africa | UNESCO Biosphere Reserve |
| 49 | 29376 | Pinselli and Soyah Classified Forests | Guinea | Africa | Not assigned |
| 50 | 29069 | Badiar National Park | Guinea | Africa | II |
| 51 | 679 | Hortobágy National Park | Hungary | Europe | UNESCO Biosphere Reserve |
| 52 | 680 | Bükk National Park | Hungary | Europe | II |
| 53 | 555539806 | Balaton Uplands National Park | Hungary | Europe | V |
| 54 | 178325 | Duna-Ipoly National Park | Hungary | Europe | V |
| 55 | 13652 | Aggtelek National Park | Hungary | Europe | II |
| 56 | 757 | Mount Kenya National Park | Kenya | Africa | II |
| 57 | 752 | Tsavo East National Park | Kenya | Africa | II |
| 58 | 761 | Nairobi National Park | Kenya | Africa | II |
| 59 | 1297 | Maasai Mara National Reserve | Kenya | Africa | II |
| 60 | 7409 | Sapo National Park | Liberia | Africa | II |
| 61 | 9170 | Grebo-Krahn National Park | Liberia | Africa | Forest reserve |
| 62 | 9176 | East Nimba Nature Reserve | Liberia | Africa | Forest reserve |
| 63 | 9171 | Gola Rainforest National Park | Liberia | Africa | Forest reserve |
| 64 | 303691 | Tsau /Khaeb National Park | Namibia | Africa | II |
| 65 | 555543018 | Dorob National Park | Namibia | Africa | II |
| 66 | 851 | Bieszczady National Park | Poland | Europe | II |
| 67 | 11144 | Magura National Park | Poland | Europe | II |
| 68 | 11147 | Polesie National Park | Poland | Europe | II |
| 69 | 850 | Świętokrzyski National Park | Poland | Europe | II |
| 70 | 848 | Tatrzański National Park | Poland | Europe | II |
| 71 | 854 | Białowieża National Park | Poland | Europe | II |
| 72 | 11151 | Domogled-Valea Cernei National Park | Romania | Europe | II |
| 73 | 555531207 | Cozia National Park | Romania | Europe | II |
| 74 | 555531386 | Semenic-Caraș Gorge National Park | Romania | Europe | II |
| 75 | 67728 | Danube Delta Biosphere Reserve | Romania | Europe | UNESCO Biosphere Reserve |
| 76 | 555540945 | Nera Gorge-Beușnița National Park | Romania | Europe | II |
| 77 | 555531377 | Retezat National Park | Romania | Europe | II |
| 78 | 7417 | Outamba-Kilimi National Park | Sierra Leone | Africa | II |
| 79 | 555542335 | Gola Rainforest National Park | Sierra Leone | Africa | II |
| 80 | 148026 | Poloniny National Park | Slovakia | Europe | V |
| 81 | 12152 | Low Tatras National Park | Slovakia | Europe | II |
| 82 | 4377 | Slovak Paradise National Park | Slovakia | Europe | II |
| 83 | 1975 | Tatra National Park | Slovakia | Europe | II |
| 84 | 4375 | Malá Fatra National Park | Slovakia | Europe | V |
| 85 | 9999999 | Addo Elephant National Park | South Africa | Africa | not reported |
| 86 | 881 | Garden Route National Park | South Africa | Africa | not reported |
| 87 | 876 | Karoo National Park | South Africa | Africa | not reported |
| 88 | 300408 | Table Mountain National Park | South Africa | Africa | not reported |
| 89 | 877 | Mountain Zebra National Park | South Africa | Africa | not reported |
| 90 | 17368 | West Coast National Park | South Africa | Africa | not reported |
| 91 | 389012 | Doñana National Park | Spain | Europe | II |
| 92 | 71213 | Picos de Europa National Park | Spain | Europe | II |
| 93 | 389011 | Sierra Nevada National Park | Spain | Europe | II |
| 94 | 196014 | Aigüestortes i Estany de Sant Maurici National Park | Spain | Europe | II |
| 95 | 349125 | Cabañeros National Park | Spain | Europe | II |
| 96 | 909 | Abisko National Park | Sweden | Europe | II |
| 97 | 905 | Padjelanta National Park | Sweden | Europe | II |
| 98 | 906 | Sarek National Park | Sweden | Europe | II |
| 99 | 907 | Muddus/Muttos National Park | Sweden | Europe | II |
| 100 | 3998 | Stora Sjöfallet National Park | Sweden | Europe | II |
| 101 | 908 | Pieljekaise National Park | Sweden | Europe | II |
| 102 | 17744 | El Feidja National Park | Tunisia | Africa | Not assigned |
| 103 | 941 | Ichkeul National Park | Tunisia | Africa | UNESCO Biosphere Reserve |
| 104 | 4487 | Bou-Hedma National Park | Tunisia | Africa | II |
| 105 | 555624270 | Jebil National Park | Tunisia | Africa | Not assigned |
| 106 | 18776 | Sidi Toui National Park | Tunisia | Africa | Not assigned |
| 107 | 555624249 | Dghoumes National Park | Tunisia | Africa | Not assigned |
| 108 | 555624261 | Orbata National Park | Tunisia | Africa | Not assigned |
| 109 | 40042 | Toro Semliki Wildlife Reserve | Uganda | Africa | II |
| 110 | 1441 | Lake Mburo National Park | Uganda | Africa | II |
| 111 | 18437 | Bwindi Impenetrable National Park | Uganda | Africa | II |
| 112 | 40002 | Kibale National Park | Uganda | Africa | II |
| 113 | 18438 | Rwenzori Mountains National Park | Uganda | Africa | II |
| 114 | 957 | Queen Elizabeth National Park | Uganda | Africa | II |

1. **ANALYSES:**

*Table B Information for all variables (response variables, test predictors, control predictors) used in the different models. The table provides the variable name (‘Variables’), the class (‘Type’), a definition (‘Definition’) for each variable, the hypotheses which each variable is based on (‘Explanations, hypothesis and references’), the source of data for each variable (‘Sources of data’), and how each variable was constructed (‘Construction of variable’).*

| **Variables** | **Type** | **Definition** | **Explanations, hypothesis, and references** | **Sources of data** | **Construction of variable** |
| --- | --- | --- | --- | --- | --- |
| HDI | Test predictor | HDI (Human development index) is a measure of the level of socioeconomic development of a nation. It is an index obtained from three measures of socioeconomic indicators: health, wealth (GDP), and education. We used the value for 2017. | We approximated the socioeconomic gradations along the studied South-North axis by using HDI. HDI reflects the multidimensional aspects of development and provides a balanced view of a country's development status. We expected an effect on hunting, as HDI has been shown to be related to the intensity of general anthropogenic pressures [1], poaching activities [2], and related wildlife population declines [3]. | United Nations Development Programme, <http://hdr.undp.org/en/composite/HDI>  Accessed 15/12/18 | HDI was assigned to each country |
| Human population density | Test Predictor | Number of people per square kilometer. We used the value for 2015. | Human population density approximates the human-domination of a regional landscape. We expected an effect on human-wildlife relationships, since human densities have been driving wildlife extinctions for millennia [4], and predict current ranges of predators [5] and large mammals [6], hunting pressure [7] and conflict-related killings and behavioral adaptations [8]. | Center for International Earth Science Information Network - CIESIN - Columbia University. 2018. Gridded Population of the World, Version 4 (GPWv4): Population Density, Revision 11. The data files were produced as global rasters at 30 arc-second (~1 km at the equator) resolution. Palisades, NY: NASA Socioeconomic Data and Applications Center (SEDAC). <https://doi.org/10.7927/H49C6VHW>. Accessed  Accessed 15/12/18 | Pixel values were extracted in a neighborhood of 10 km around each PA and the mean was calculated |
| Community characteristics | Test predictor/response (follow-up analyses) | A composite index obtained from three independent indices: nature-friendly “*Culture”, “Attitude”*, and “*Mutual trust”* levels between communities and the PA management. | We expected a positive effect of supportive local community attributes, as they can enhance conservation outcomes, even against a pre-existing national context [9,10]. For the creation of the index, we combined theories on community-based conservation and commons theory and extracted three main attributes. ‘Attitudes’ [9,10] and local ‘culture’ [11] are shown to benefit conservation outcomes, meanwhile ‘mutual trust’ is central to any collaboration, including sustainable resource use [12,13], conservation [14] and conflict-resolution [15]. | *Item="Culture” (Index)*  Part VII: Qu.1. The culture of the local community is supportive of nature conservation  Qu. 2 The local communities have institutions related to nature conservation  Qu. 3. Local communities are willing to participate in nature conservation  Part VI. Qu. 7 The local communities support the protection of the NP  Qu. 4. The local communities would like to protect the wildlife in the NP  *Item= “Attitude” (index)*  Qu. 1 The local communities have positive attitude towards the NP  Qu. 2 The local communities have positive attitude towards the park authorities  Qu 3. The local communities are satisfied with the management of the NP  Part Involvement, Qu. 3 The local communities are satisfied with the benefits they get from the NP  *Item: “Trust” (index)*  Part VII: Qu. 5 I trust the local community that they protect the NP  Part VI Qu. 3 The local communities trust the park management and authorities | We standardized all variables to a range from 0 to 1. Before standardizing, variables collected on opposite scales were reversed to equalize the interpretation direction, and skewed variables were log or squared transformed to achieve a more normal distribution. The index was derived by averaging the variables per national park |
| Protection-based conservation interventions | Test predictor | A composite index obtained from three protection based interventions, regular ranger patrols, buffer zone, and presence of permanent research institutions. | We expected a positive effect of protection-based efforts that focus on protecting resources within PAs. Higher patrolling effort is shown to increase wildlife abundances [16], and buffer zones can mitigate the input of damage from the immediate surroundings [17]. We also included the presence of a research station, as the presence of staff can have a similar protective effect as ranger patrols [18] | Part: III. Conservation interventions, Question  a) Regular patrol  b) Buffer zone  f) Presence of permanent research institution | We standardized all variables to a range from 0 to 1. Before standardizing variables collected on opposite scales were reversed to equalize the interpretation direction and skewed variables were log or squared transformed to achieve a more normal distribution. The index was derived by averaging the variables per national park |
| Community-based conservation interventions | Test predictor | A composite index was obtained from four independent indices: “*Involvement”, “Economic benefits”, “Livelihood benefits”, “Awareness creation”*. | We expected a positive effect of community based conservation interventions that focus on increasing local socioeconomic conditions around PAs. We focus here on the four most common strategies. Institutional settings; in particular inclusion have proven central to conservation outcomes [11,14]. | *Item= “Involvement” (index):*  Part VI, Involvement, Qu.1 The local communities are involved in the decision-making process of the NP  Qu.5 The local communities participate in the protection of the NP  Qu. 6 The village chiefs/leaders participate in the decision-making process of the PA  *Item= “Economic benefits” (index).*  Part V: Qu 1. Associated benefits for local communities in terms of community projects such as schools, medical homes, roads, and others...  Qu. 2. Economic benefits to local communities from the NP in terms of income, employment...  Qu. 5 Benefits to local communities through tourism  Qu. 8. Legal right to access some resources from the NP  Part III, Qu l. Payment for local communities/farmers  *Item=” Livelihood benefits” (index)*  p. Alternative livelihood projects  m. Provision of community projects: schools/health facilities/roads/others  *Item =“Awareness” (index):*  Part III. Qu. q Environmental/conservation education and awareness creation | We standardized all variables to a range from 0 to 1. Before standardizing, variables collected on opposite scales were reversed to equalize the interpretation direction and skewed variables were log or squared transformed to achieve a more normal distribution. The index was derived by averaging the variables per national park. |
| Function of hunting (Hunting index:high, low) | Test predictor/ Interaction term | Economic, socio-cultural, and ecological function of hunting; Economic function: hunting for subsistence and income, socio-cultural function: non-market related hunting for entertainment and cultural reasons, ecological function: killing because of population control and human-wildlife conflict | We expected changing motivation of hunting across the HDI gradient reflecting varying socioeconomic conditions: a strong economic function of hunting due to reliance on hunting for livelihoods in the South [19], and in the North a stronger ecological function due to an increased control-based wildlife approach [20] and lack of regulative functions provided by predators [5]. We expected the social function to be equal across the range, as PAs primary goal is biodiversity protection. | Part VI  Qu. 12, Motivations for killing animals from the PA | We standardized the answers: (1=main motivation, 2= secondary motivation, 3= minor motivation, 4= no motivation) (index range of 0 to 1). We reversed the index (0= lowest, 1= highest). We further grouped ecological hunting (hunting for control + HWCs), economic hunting (subsistence+ commercial hunting) and socio-cultural hunting (culture + recreation). The index was derived by averaging the variables. We derived the hunting index by grouping the main and secondary motivation as high (major motivation) and minor, and no motivation as low (neglectable motivation). Therefore we defined for the standardized and grouped variable > 0.5 as high (1), <=0.5 as low. (0) We conducted sensitivity analysis considering >0.5 or >= as high (Table C and D in Appendix), but the effects did not change.  We tested the effect of the single categories based on an interaction between the variable 'hunting function' with its three levels and all main predictors (HDI, population density, community attributes). |
| Threat rating “hunting/poaching”, (legal, illegal).  (Threat: high, low) | Response | Assessment of threat by “Hunting and trapping” | We expected a decreasing threat by hunting along the HDI gradient [2], since increasing prosperity reduces hunting pressure related to maintaining livelihoods [19]. | Part IV  Qu. 35, Severity of the threat “hunting” to biodiversity conservation in your NP | We standardized the values, 1=very high, 2=high, 3=moderate, 4=low, 5 very low (index range of 0-1). We reversed the index to 0=lowest and 1=highest value. We split the variable into >0.5 (very high, high)=high, and <=0.5 (moderate, low, very low as low.)=low. We ran sensitivity analysis including also moderate as high, but effects did not change (see table S8/S9). |
| Threat rating “killing because of human-wildlife-conflict”, (legal, illegal).  (Threat: high, low) | Response | Assessment of threat by “Killing animals because of human-wildlife conflict” | We expect higher human-wildlife conflicts in the Global South as the proportional economic loss due to HWCs is here higher than in the North [21]. Nevertheless, more HWCs are reported in the Global North, which might be related to research biases [22]. We expected a decreasing threat of HWC with decreasing human densities as a function of lower encounter rates and potential conflicts. | Part IV  Qu. 35, Severity of the threat “Killing animals because of human-wildlife conflict” to biodiversity conservation in your NP | We standardized the values, 1=very high, 2=high, 3=moderate, 4=low, 5 very low (index range of 0-1). We reversed the index to 0=lowest and 1=highest value. We split the variable into >0.5 (very high, high)=high, and <=0.5 (moderate, low, very low as low.)=low. We ran sensitivity analysis including also moderate as high, but effects did not change (see table ). |
| Threat of illegal hunting by trophic level of threatened species. (Threatened: yes, no) |  | Assessment of species listed by respondents as threatened by illegal hunting, divided in predators including raptors, predatory mammals, versus non-predatory containing primates, apes, omnivorous, frugivorous, insectivorous mammals and non-predatory birds | We expected changes in the target animals related to changing motivations across the S-N gradient. |  | We grouped the species threatened by illegal hunting species into predators and non-predators (predator=1, non-predator=0). |
| Rating of the threat to wildlife from parks through illegal hunting (killing/poaching/poisoning) within the administrative PA boundaries.  (Threat: high, low). | Response | Assessment “Illegal killing/poaching/poisoning is a severe threat for protected species in the NP” | We expect a decreasing threat by illegal hunting within parks over the South-North gradient, since increasing prosperity decreases economic pressure to take high risks of illegal hunting within parks. | Part VI  Qu. 3,1  Illegal killing/poaching/poisoning is a severe threat for protected species in the NP | We standardized the values, 1=strongly agree, 2=agree, 3=slightly agree, 4=slightly disagree, 5=disagree, 6=strongly disagree (index range of 0-1). We reversed the index to 0=lowest and 1=highest value. We split >0.5 (strongly agree, agree, slightly agree)=high, and <0.5 (slightly disagree, disagree, strongly disagree)=low. |
| Rating of the threat to wildlife from parks through illegal hunting (killing/poaching/poisoning) outside the administrative PA boundaries.  (Threat: high, low). | Response | Assessment of “Illegal killing/poaching/poisoning is a severe threat for protected species of the NP outside the NP” | We expected a less pronounced decreasing threat by illegal hunting outside parks over the South-North gradient, as illegal hunting outside PAs is connected to lower risks and might also be conflict-driven. | Part VI  Qu. 3,2  Illegal killing/poaching/poisoning is a severe threat to protected species of the NP outside the NP | We standardized the values, 1=strongly agree, 2=agree, 3=slightly agree, 4=slightly disagree, 5=disagree, 6=strongly disagree (index of 0-1). We split >0.5 (strongly agree, agree, slightly agree) =high, and <0.5 (slightly disagree, disagree, strongly disagree) = low. |
| PA size | Control | Size of PA in km^2^ | We expected human disturbance and the threat of hunting less within larger PAs [23]. | Protected planet database  <https://www.protectedplanet.net/>  Accessed 15/12/18. |  |
| Country | Random effect | The country and the continent the PA is located in | We included the country as a control for unaccounted national effects, such as different rules or regulations. | Our questionnaire |  |
| Change in the abundance of mammals and birds | Control | Mean of abundance changes over all species (changes in abundance 1, when increasing/stable, 0 when decreasing) | We expected decreasing abundances when hunting pressure increases [24]. | Part IV, Biodiversity related questions | We derived a binary variable assuming 1 if the change is stable/improving or 0 otherwise in the past 10 years. We used the aggregated mean over all species. |
| Change in the abundance of predators | Control | Mean of abundance changes over all predatory species (changes in abundance 1, increasing/stable, 0 decreasing) | We expect decreasing abundances when hunting pressure increases [24]. | Part IV, Biodiversity related questions | We derived a binary variable assuming 1 if the change is stable/improving or 0 otherwise in the past 10 years. We used the aggregated mean over all predatory species. |

*1 All variables were standardized from zero to one before compiling the indices. All predictor variables were transformed into standard normal z distribution (with mean 0 and standard deviation 1).*

*
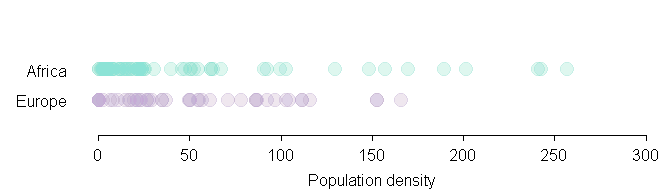
Fig. A Distribution of population density along the gradient in Europe and Africa*

*
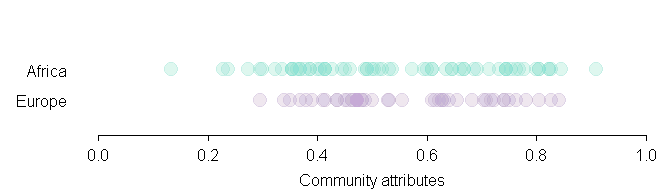
*

*Fig. B Distribution of community attributes along the gradient in Europe and Africa*

1. **RESULTS:**

*Table C Model results on the probability of prevailing ecological, cultural, and social functions of killing of wildlife (break of binary predictor <0.5). Predictors were standardized so slopes are comparable.*

| **Population-Level Effects:** | | |  |  |
| --- | --- | --- | --- | --- |
|  | **Estimate** | **Est.Error** | **l-95% CI** | **u-95% CI** |
| Intercept | -2.14 | 0.43 | -3.02 | -1.34 |
| HDI (2017) | 0.74 | 0.36 | 0.04 | 1.45 |
| Economic_hunting | 1.78 | 0.54 | 0.69 | 2.82 |
| Social_hunting | 0.03 | 0.5 | -0.95 | 0.99 |
| Population density (log) | -0.09 | 0.38 | -0.83 | 0.68 |
| Community characteristics index | 0.41 | 0.31 | -0.19 | 1.04 |
| Size (log) | -0.06 | 0.28 | -0.61 | 0.49 |
| HDI (2017):  Economic_hunting | -2.86 | 0.53 | -3.93 | -1.84 |
| HDI (2017):  Social_hunting | 1.01 | 0.52 | 0.03 | 2.06 |
| Economic_hunting:  Population density (log) | -0.28 | 0.52 | -1.32 | 0.72 |
| Social_hunting:  Population density (log) | 0.22 | 0.53 | -0.85 | 1.23 |
| Economic_hunting:  Community characteristics index | -0.44 | 0.43 | -1.28 | 0.4 |
| Social_hunting:  Community characteristics index | -0.48 | 0.45 | -1.35 | 0.41 |

*Table D Model results of the sensibility analysis. The probability of prevailing ecological, cultural and social functions of killing of wildlife (break of binary predictor <=0.5)*

| **Population-Level Effects:** | | |  |  |
| --- | --- | --- | --- | --- |
|  | **Estimate** | **Est.Error** | **l-95% CI** | **u-95% CI** |
| Intercept | -0.50 | 0.41 | -1.35 | 0.22 |
| HDI (2017) | 0.61 | 0.33 | -0.04 | 1.26 |
| Economic_hunting | 1.24 | 0.50 | 0.25 | 2.23 |
| Social_hunting | -0.16 | 0.39 | -0.95 | 0.60 |
| Population density (log) | 0.38 | 0.35 | -0.30 | 1.09 |
| Community characteristics index | 0.41 | 0.27 | -0.10 | 0.95 |
| Size (log) | 0.17 | 0.23 | -0.28 | 0.62 |
| HDI (2017):  Economic_hunting | -2.11 | 0.51 | -3.15 | -1.11 |
| HDI (2017):  Social_hunting | 0.33 | 0.40 | -0.43 | 1.12 |
| Economic_hunting:  Population density (log) | -0.57 | 0.51 | -1.58 | 0.42 |
| Social_hunting:  Population density (log) | 0.00 | 0.50 | -0.98 | 0.98 |
| Economic_hunting:  Community characteristics index | -0.66 | 0.42 | -1.48 | 0.17 |
| Social_hunting:  Community characteristics index | -0.52 | 0.40 | -1.31 | 0.27 |

*Table E Probability of high impact of hunting/poaching including very high, high)*

| **Population-Level Effects:** | | |  |  |
| --- | --- | --- | --- | --- |
|  | **Estimate** | **Est.Error** | **l-95% CI** | **u-95% CI** |
| Intercept | -1.28 | 0.52 | -2.38 | -0.34 |
| Population density (log) | -0.34 | 0.46 | -1.23 | 0.58 |
| HDI (2017) | -1.58 | 0.48 | -2.59 | -0.65 |
| Community characteristics index | -0.5 | 0.45 | -1.39 | 0.38 |
| Protection-based interventions | -0.26 | 0.42 | -1.12 | 0.55 |
| Community-based interventions index | -0.42 | 0.47 | -1.39 | 0.52 |
| Abundance (mean) | -0.76 | 0.44 | -1.69 | 0.06 |
| Size (log) | 0.13 | 0.49 | -0.78 | 1.14 |

*Table F Probability of high impact of hunting/poaching (including very high, high, moderate)(sensitivity analysis)*

| **Population-Level Effects:** | | |  |  |
| --- | --- | --- | --- | --- |
|  | **Estimate** | **Est.Error** | **l-95% CI** | **u-95% CI** |
| Intercept | 1.09 | 0.6 | -0.06 | 2.32 |
| Population density (log) | -0.07 | 0.48 | -1.04 | 0.88 |
| HDI (2017) | -1.43 | 0.51 | -2.49 | -0.46 |
| Community characteristics index | -0.54 | 0.46 | -1.45 | 0.35 |
| Protection-based interventions | -0.45 | 0.45 | -1.36 | 0.42 |
| Community-based interventions index | -0.36 | 0.52 | -1.38 | 0.67 |
| Abundance (mean) | -0.74 | 0.5 | -1.77 | 0.18 |
| Size (log) | 0.51 | 0.51 | -0.49 | 1.53 |

*Table G Probability of high impact of poaching (Including very high, high, only illegal poaching), n=73*

| **Population-Level Effects:** | | |  |  |
| --- | --- | --- | --- | --- |
|  | Estimate | Est.Error | l-95% CI | u-95% CI |
| Intercept | -1.73 | 0.67 | -3.18 | -0.49 |
| Population density (log) | -0.33 | 0.5 | -1.32 | 0.66 |
| HDI (2017) | -1.7 | 0.57 | -2.79 | -0.62 |
| Community characteristics index | -0.5 | 0.48 | -1.45 | 0.44 |
| Protection-based interventions | -0.35 | 0.46 | -1.26 | 0.55 |
| Community-based interventions index | -0.31 | 0.51 | -1.31 | 0.67 |
| Abundance (mean) | -0.37 | 0.47 | -1.34 | 0.54 |
| Size (log) | -0.07 | 0.52 | -1.06 | 1.01 |

*Table H Probability of high killing rate through human-wildlife-conflicts (including very high, high)*

| **Population-Level Effects:** | | |  |  |
| --- | --- | --- | --- | --- |
|  | **Estimate** | **Est.Error** | **l-95% CI** | **u-95% CI** |
| Intercept | -2.58 | 0.67 | -4.11 | -1.43 |
| Population density (log) | 0.96 | 0.53 | -0.06 | 2.04 |
| HDI (2017) | 0.37 | 0.5 | -0.62 | 1.35 |
| Community characteristics index | -0.52 | 0.47 | -1.47 | 0.4 |
| Protection-based interventions | -0.36 | 0.46 | -1.27 | 0.54 |
| Community-based interventions index | 0.4 | 0.49 | -0.55 | 1.39 |
| Abundance (mean) | -0.59 | 0.48 | -1.57 | 0.37 |
| Size (log) | 0.61 | 0.5 | -0.4 | 1.58 |

*Table I Probability of high killing rate due to human-wildlife-conflicts (including very high, high, moderate) (sensitivity analysis)*

| **Population-Level Effects:** | | |  |  |
| --- | --- | --- | --- | --- |
|  | **Estimate** | **Est.Error** | **l-95% CI** | **u-95% CI** |
| Intercept | -1 | 0.57 | -2.18 | 0.04 |
| Population density (log) | 0.87 | 0.52 | -0.14 | 1.93 |
| HDI (2017) | -0.35 | 0.49 | -1.32 | 0.61 |
| Community characteristics index | -0.53 | 0.44 | -1.42 | 0.31 |
| Protection-based interventions | -0.01 | 0.42 | -0.84 | 0.83 |
| Community-based interventions index | 0.09 | 0.46 | -0.82 | 1 |
| Abundance (mean) | -0.13 | 0.44 | -1 | 0.72 |
| Size (log) | 0.29 | 0.47 | -0.63 | 1.22 |

*Table J Probability that predators are threatened by killing of wildlife*

| **Population-Level Effects:** | | |  |  |
| --- | --- | --- | --- | --- |
|  | **Estimate** | **Est.Error** | **l-95% CI** | **u-95% CI** |
| Intercept | -0.99 | 0.98 | -2.91 | 0.93 |
| HDI (2017) | 1.22 | 0.62 | -0.11 | 2.36 |
| Size (log) | -0.2 | 0.46 | -1.13 | 0.68 |
| Community characteristics index | -0.06 | 0.33 | -0.72 | 0.6 |
| Population density (log) | 0.14 | 0.5 | -0.84 | 1.14 |
| CommunityIntervention_index | -0.24 | 0.94 | -2.09 | 1.61 |
| Protection_3_interv | -0.36 | 0.78 | -1.88 | 1.16 |
| z_Abundance_predMean | 0.57 | 0.45 | -0.31 | 1.49 |

*Table K Probability of high threat to animals in the PA*

| **Population-Level Effects:** | | |  |  |
| --- | --- | --- | --- | --- |
|  | **Estimate** | **Est.Error** | **l-95% CI** | **u-95% CI** |
| Intercept | 1.5 | 0.55 | 0.48 | 2.65 |
| HDI (2017) | -1.44 | 0.51 | -2.46 | -0.46 |
| Community characteristics index | -0.99 | 0.46 | -1.91 | -0.12 |
| Protection-based interventions | -0.58 | 0.45 | -1.51 | 0.28 |
| Community-based interventions index | 0.46 | 0.51 | -0.55 | 1.45 |
| Population density (log) | -0.61 | 0.5 | -1.59 | 0.4 |
| Size (log) | 0.28 | 0.51 | -0.69 | 1.31 |
| Abundance (mean) | -0.36 | 0.48 | -1.32 | 0.57 |

*Table L Probability of high threat to animals outside the PA*

| **Population-Level Effects:** | | |  |  |
| --- | --- | --- | --- | --- |
|  | **Estimate** | **Est.Error** | **l-95% CI** | **u-95% CI** |
| Intercept | 2 | 0.63 | 0.88 | 3.35 |
| HDI (2017) | -0.95 | 0.49 | -1.94 | -0.01 |
| Community characteristics index | -0.89 | 0.47 | -1.83 | 0.01 |
| Protection-based interventions | 0.07 | 0.47 | -0.86 | 1.02 |
| Community-based interventions index | -0.13 | 0.51 | -1.15 | 0.84 |
| Population density (log) | -0.44 | 0.54 | -1.49 | 0.66 |
| Size (log) | -0.05 | 0.5 | -1.02 | 0.94 |
| Abundance (mean) | -0.56 | 0.5 | -1.57 | 0.38 |

*Table M Effect of trust towards PA management on the probability that animals are threatened from poaching inside the PA*

| **Population-Level Effects:** | | |  |  |
| --- | --- | --- | --- | --- |
|  | **Estimate** | **Est.Error** | **l-95% CI** | **u-95% CI** |
| Intercept | 1.5 | 0.55 | 0.48 | 2.63 |
| HDI (2017) | -1.54 | 0.5 | -2.56 | -0.57 |
| Trust index | -1.26 | 0.48 | -2.23 | -0.34 |
| Protection-based interventions | -0.55 | 0.44 | -1.43 | 0.32 |
| Community-based interventions index | 0.38 | 0.5 | -0.64 | 1.37 |
| Population density (log) | -0.6 | 0.5 | -1.59 | 0.39 |
| Size (log) | 0.31 | 0.51 | -0.65 | 1.35 |
| Abundance (mean) | -0.44 | 0.48 | -1.42 | 0.49 |

*Table N Effect of trust towards PA management on the probability that animals are threatened from poaching outside the PA*

| **Population-Level Effects:** | | |  |  |
| --- | --- | --- | --- | --- |
|  | **Estimate** | **Est.Error** | **l-95% CI** | **u-95% CI** |
| Intercept | 1.95 | 0.6 | 0.87 | 3.2 |
| HDI (2017) | -0.94 | 0.49 | -1.92 | 0.04 |
| Trust index | -0.82 | 0.46 | -1.73 | 0.03 |
| Protection-based interventions | 0.08 | 0.45 | -0.78 | 1 |
| Community-based interventions index | -0.27 | 0.49 | -1.24 | 0.65 |
| Population density (log) | -0.42 | 0.56 | -1.51 | 0.72 |
| Size (log) | -0.04 | 0.5 | -1.01 | 0.96 |
| Abundance (mean) | -0.63 | 0.5 | -1.65 | 0.32 |

*Table O Effect of attitudes towards PA management on the probability that animals are threatened from poaching inside the PA*

| **Population-Level Effects:** | | |  |  |
| --- | --- | --- | --- | --- |
|  | **Estimate** | **Est.Error** | **l-95% CI** | **u-95% CI** |
| Intercept | 1.45 | 0.56 | 0.44 | 2.63 |
| HDI (2017) | -1.37 | 0.51 | -2.38 | -0.36 |
| Attitude index | -0.82 | 0.44 | -1.72 | 0.02 |
| Protection-based interventions | -0.63 | 0.45 | -1.56 | 0.24 |
| Community-based interventions index | 0.41 | 0.51 | -0.64 | 1.38 |
| Population density (log) | -0.59 | 0.51 | -1.57 | 0.43 |
| Size (log) | 0.26 | 0.5 | -0.7 | 1.27 |
| Abundance (mean) | -0.4 | 0.49 | -1.37 | 0.53 |

*Table P Effect of attitudes towards PA management on the probability that animals are threatened from poaching outside the PA*

| **Population-Level Effects:** | | |  |  |
| --- | --- | --- | --- | --- |
|  | **Estimate** | **Est.Error** | **l-95% CI** | **u-95% CI** |
| Intercept | 2.06 | 0.65 | 0.92 | 3.43 |
| HDI (2017) | -0.82 | 0.52 | -1.9 | 0.16 |
| Attitude index | -0.71 | 0.49 | -1.72 | 0.27 |
| Protection-based interventions | -0.03 | 0.49 | -1 | 0.94 |
| Community-based interventions index | -0.17 | 0.51 | -1.18 | 0.83 |
| Population density (log) | -0.46 | 0.55 | -1.52 | 0.64 |
| Size (log) | -0.06 | 0.5 | -1.05 | 0.92 |
| Abundance (mean) | -0.61 | 0.5 | -1.63 | 0.36 |

*Table Q Effects of supportive local culture on the probability that animals are threatened from poaching inside the PA*

| **Population-Level Effects:** | | |  |  |
| --- | --- | --- | --- | --- |
|  | **Estimate** | **Est.Error** | **l-95% CI** | **u-95% CI** |
| Intercept | 1.49 | 0.57 | 0.47 | 2.71 |
| HDI (2017) | -1.41 | 0.5 | -2.44 | -0.48 |
| Local culture index | -0.7 | 0.44 | -1.6 | 0.14 |
| Protection-based interventions | -0.56 | 0.46 | -1.51 | 0.32 |
| Community-based interventions index | 0.34 | 0.49 | -0.6 | 1.32 |
| Population density (log) | -0.56 | 0.53 | -1.59 | 0.49 |
| Size (log) | 0.34 | 0.51 | -0.6 | 1.38 |
| Abundance (mean) | -0.4 | 0.47 | -1.34 | 0.52 |

*Table R Effects of supportive local culture on the probability that animals are threatened from poaching outside the PA*

| **Population-Level Effects:** | | |  |  |
| --- | --- | --- | --- | --- |
|  | **Estimate** | **Est.Error** | **l-95% CI** | **u-95% CI** |
| Intercept | 1.96 | 0.62 | 0.89 | 3.34 |
| HDI (2017) | -0.98 | 0.5 | -2.01 | -0.01 |
| Local culture index | -0.88 | 0.45 | -1.82 | -0.03 |
| Protection-based interventions | 0.13 | 0.46 | -0.76 | 1.07 |
| Community-based interventions index | -0.2 | 0.48 | -1.14 | 0.74 |
| Population density (log) | -0.42 | 0.55 | -1.49 | 0.72 |
| Size (log) | 0.02 | 0.5 | -0.91 | 1.04 |
| Abundance (mean) | -0.58 | 0.49 | -1.61 | 0.36 |

*Table S AIC-Values and ∆AIC of models containing only compositions of the indices for community characteristics. Values were estimated separately for “model location in” and “model location out”.*

| **Model** | **AIC** | **∆AIC** |
| --- | --- | --- |
| Model location in Trust | 121.44 | 0.00 |
| Model location in | 128.35 | 6.91 |
| Model location in Attitude | 131.23 | 9.78 |
| Model location in Culture | 132.41 | 10.96 |
| Model location out Trust | 125.08 | 0.00 |
| Model location in out | 125.64 | 0.56 |
| Model location out Culture | 126.09 | 1.00 |
| Model location Attitude | 127.80 | 2.72 |

*∆AIC > 2 indicates that the model receives 'considerably less' empirical support and models that differ from the best model by more than 10 are 'substantially' worse than the best and unlikely to be the truly best model.*

*Table T Model results for the probability of beneficial local community conditions*

| **Population-Level Effects:** | |  |  |  |
| --- | --- | --- | --- | --- |
|  | **Estimate** | **Est.Error** | **l-95% CI** | **u-95% CI** |
| Intercept | -0.23 | 1.38 | -3.11 | 2.35 |
| Population density (log) | -0.24 | 0.64 | -1.55 | 1.01 |
| Provision economic benefits | 0.72 | 0.65 | -0.67 | 1.93 |
| Implementation livelihood projects | -0.25 | 0.58 | -1.4 | 0.92 |
| Scale of local inclusion | 1.4 | 0.75 | -0.21 | 2.77 |
| Implementation environmental awareness (No) | 0.33 | 0.92 | -1.46 | 2.19 |
| Size (log) | -0.61 | 0.62 | -1.79 | 0.68 |

*Table U Legally and illegal killed species*

| No park | Country | Region | Species | Scientific name | Species guild | N° hun-ted | Unit | Legal setting | Reason | Weapon |
| --- | --- | --- | --- | --- | --- | --- | --- | --- | --- | --- |
| 1 | Kenya | East Africa | Animals | NA | NA | 0 | per year | legal | NA | NA |
| 2 | Uganda | East Africa | Animals | NA | NA | 0 | per year | legal | NA | NA |
| 3 | Ethiopia | East Africa | Animals | NA | NA | 0 | per year | legal | NA | NA |
| 4 | Ethiopia | East Africa | Animals | NA | NA | 0 | per year | legal | NA | NA |
| 5 | Austria | West Europe | NA | Animals | NA | 0 | NA | illegal | NA | NA |
| 5 | Austria | West Europe | Red deer | *Cervus elaphus* | Large non-predatory mammals | 150 | per year | legal | population control, protection of flora | NA |
| 5 | Austria | West Europe | Wild pig | *Sus scrofa* | Omnivorous mammals | 400 | per year | legal | population control, protection of flora | NA |
| 6 | Austria | West Europe | Chamois | *Rupicapra rupicapra* | Small to medium non-predatory mammals | 0.3 | per year | illegal | Hunting/ poaching | NA |
| 6 | Austria | West Europe | Chamois | *Rupicapra rupicapra* | Small to medium non-predatory mammals | 150 | per year | legal | population control | NA |
| 6 | Austria | West Europe | Red deer | *Cervus elaphus* | Large non-predatory mammals | 150 | per year | legal | population control | NA |
| 6 | Austria | West Europe | Roe deer | *Capreolus capreolus* | Small to medium non-predatory mammals | 150 | per year | legal | population control | NA |
| 7 | Austria | West Europe | Animals | NA | NA | 0 | NA | illegal | NA | NA |
| 7 | Austria | West Europe | Wild pig | *Sus scrofa* | Omnivorous mammals | 50 | per year | legal | population control | NA |
| 8 | Hungary | East Europe | Hooded crow | *Corvus cornix* | Omnivorous birds | 280 | 2016/2017 | legal | population control | traps |
| 8 | Hungary | East Europe | common_magpie | *Pica pica* | Omnivorous birds | 60 | 2016/2017 | legal | population control | traps |
| 8 | Hungary | East Europe | red_fox | *Vulpes vulpes* | Small mammalian predators | 540 | 2016/2017 | legal | population control | traps/shooting |
| 8 | Hungary | East Europe | European_badger | *Meles meles* | Omnivorous mammals | 15 | 2016/2017 | legal | population control | traps |
| 8 | Hungary | East Europe | Wild pig | *Sus scrofa* | Omnivorous mammals | 420 | 2016/2017 | legal | population control | shooting |
| 8 | Hungary | East Europe | Red deer | *Cervus elaphus* | Large non-predatory mammals | 170 | 2016/2017 | legal | population control | shooting |
| 9 | Hungary | East Europe | wolf | *Canis lupus* | Top mammalian predators | 1 | per year | illegal | human wildlife conflict | shooting |
| 9 | Hungary | East Europe | Animals | NA | NA | 0 | per year | legal | NA | NA |
| 10 | Hungary | East Europe | hunting wild boar, deer, roc | NA | NA | 600 | per year | legal | population control | NA |
| 11 | Hungary | East Europe | coypu | *Myocastor coypus* | NA | 10 | NA | illegal | NA | NA |
| 11 | Hungary | East Europe | Animals | NA | NA | 0 | per year | legal | NA | NA |
| 12 | Hungary | East Europe | wolf | *Canis lupus* | Top mammalian predators | 1 | 2015 | illegal | NA | accident in with a fence |
| 12 | Hungary | East Europe | Wild pig | *Sus scrofa* | Omnivorous mammals | 160-240 | per year | legal | reduce damage to vegitation and ground dewelling species | NA |
| 13 | Uganda | East Africa | Animals | NA | NA | 0 | per year | legal | NA | NA |
| 14 | Uganda | East Africa | Animals | NA | NA | 0 | per year | legal | NA | NA |
| 15 | Uganda | East Africa | sitatunga | *Tragelaphus spekii* | Large non-predatory mammals | 20 | per year | legal | population control | NA |
| 16 | Kenya | East Africa | Animals | NA | NA | 0 | per year | legal | NA | NA |
| 17 | Ghana | West Africa | Animals | NA | NA | 0 | per year | legal | NA | NA |
| 18 | Ghana | West Africa | Animals | NA | NA | 0 | per year | legal | NA | NA |
| 19 | Ghana | West Africa | Animals | NA | NA | 0 | per year | legal | NA | NA |
| 20 | Ghana | West Africa | Animals | NA | NA | 0 | per year | legal | NA | NA |
| 21 | Ghana | West Africa | Animals | NA | NA | 0 | per year | legal | NA | NA |
| 22 | Liberia | West Africa | Animals | NA | NA | 0 | per year | legal | NA | NA |
| 23 | Liberia | West Africa | Animals | NA | NA | 0 | per year | legal | NA | NA |
| 24 | Liberia | West Africa | Animals | NA | NA | 0 | per year | legal | NA | NA |
| 25 | Liberia | West Africa | Hippopotamus | *Hippopotamus spec.* | Large non-predatory mammals | 1 | 2014 | illegal | human consumption | Snare |
| 25 | Liberia | East Africa | Chimpanzee | *Pan troglodytes* | Great apes | 1 | 2017 | illegal | human consumption | firearm |
| 25 | Liberia | East Africa | Red colobus | *Piliocolobus badius* | Small bodied primates | 20 | per year | illegal | Commercial | firearm |
| 25 | Liberia | East Africa | Animals | NA | NA | 0 | per year | legal | NA | NA |
| 26 | Kenya | East Africa | Animals | NA | NA | 0 | per year | legal | NA | NA |
| 27 | Kenya | West Africa | Animals | NA | NA | 0 | per year | legal | NA | NA |
| 28 | Ethiopia | West Africa | Grants Gazells | NA*nger granti* | NA | 150 | per year | legal | population control | NA |
| 28 | Ethiopia | West Africa | Greater Kudu | *Tragelaphus strepsiceros* | NA | 150 | per year | legal | population control | NA |
| 28 | Ethiopia | West Africa | Lesser kudu | *Tragelaphus imberbis* | NA | 150 | per year | legal | population control | NA |
| 28 | Ethiopia | West Africa | waterbuck | *Kobus ellipsiprymnus* | Large non-predatory mammals | 150 | per year | legal | population control | NA |
| 28 | Ethiopia | West Africa | warthogs | *Phacochoerus africanus* | Omnivorous mammals | 150 | per year | legal | population control | NA |
| 28 | Ethiopia | West Africa | Bushbuck | *Tragelaphus spec.* | Small to medium non-predatory mammals | 150 | per year | legal | population control | NA |
| 28 | Ethiopia | West Africa | Red river hog | *Potamochoerus porcus* | Omnivorous mammals | 150 | per year | legal | population control | NA |
| 29 | Sierra Leone | East Africa | Animals | NA | NA | 0 | per year | legal | NA | NA |
| 30 | Sierra Leone | East Africa | Animals | NA | NA | 0 | per year | legal | NA | NA |
| 31 | Uganda | East Africa | Animals | NA | NA | 0 | per year | legal | NA | NA |
| 32 | Uganda | West Europe | Animals | NA | NA | 0 | per year | legal | NA | NA |
| 33 | Ethiopia | East Europe | Animals | NA | NA | 0 | per year | legal | NA | NA |
| 34 | Germany | East Europe | Animals | NA | NA | 0 | NA | illegal | NA | NA |
| 34 | Germany | East Europe | wild pig | *Sus scrofa* | Omnivorous mammals | 284 | per year | legal | population control | NA |
| 35 | Romania | North Africa | Animals | NA | NA | 0 | per year | legal | NA | NA |
| 36 | Romania | North Africa | Animals | NA | NA | 0 | per year | illegal | NA | NA |
| 36 | Romania | North Africa | Animals | NA | NA | 0 | per year | legal | NA | NA |
| 37 | Tunisia | North Africa | Animals | NA | NA | 0 | per year | legal | NA | NA |
| 38 | Tunisia | North Africa | Animals | NA | NA | 0 | per year | legal | NA | NA |
| 39 | Tunisia | North Africa | Animals | NA | NA | 0 | per year | legal | NA | NA |
| 40 | Tunisia | North Africa | Jackals | *Canis adustus* | Small mammalian predators | 10 | per year | legal | NA | NA |
| 41 | Tunisia | North Africa | Animals | NA | NA | 0 | per year | legal | NA | NA |
| 42 | Cameroon | West Africa | Giant Ground Pangolin | *Smutsia gigantea* | Insectivorous mammals | 8 legs | 2015 | illegal | Hunting/poaching | NA |
| 42 | Cameroon | West Africa | African buffalo | *Syncerus caffer* | Large non-predatory mammals | 21 legs, 12 trophies | 2015 | illegal | Hunting/poaching | NA |
| 42 | Cameroon | West Africa | Red river hog | *Potamochoerus porcus* | Omnivorous mammals | 2 animals+ 45 legs | 2015 | illegal | Hunting/poaching | NA |
| 42 | Cameroon | West Africa | Sitatunga | *Tragelaphus spekii* | Large non-predatory mammals | 6 animals+ 9 legs | 2015 | illegal | Hunting/poaching | NA |
| 42 | Cameroon | West Africa | Bay Duiker | *Cephalophus dorsalis* | Small to medium non-predatory mammals | 13 animals+ 94 legs+ 12 trophies ( 6 skins) | 2015 | illegal | Hunting/poaching | NA |
| 42 | Cameroon | West Africa | Blue duiker | *Philantomba monticola* | Small to medium non-predatory mammals | 38 animals+ 25 legs, 138 trophies | 2015 | illegal | Hunting/poaching | NA |
| 42 | Cameroon | West Africa | Cynocéphale | *Cynophalus spp* | NA | 2 animals | 2015 | illegal | Hunting/poaching | NA |
| 42 | Cameroon | West Africa | Bongo | *Tragelaphus eurycerus.* | NA | 16 legs | 2015 | illegal | Hunting/poaching | NA |
| 42 | Cameroon | West Africa | Water chevrotain | *Hyemoschus aquaticus* | Small to medium non-predatory mammals | 5 legs + 6 trophies (skin) | 2015 | illegal | Hunting/poaching | NA |
| 42 | Cameroon | West Africa | Chimpanzee | *Pan troglodytes* | Great apes | 3 animals | 2015 | illegal | Hunting/poaching | NA |
| 42 | Cameroon | West Africa | Gambian pouched rat | *Cricetomys gambianus* | NA | 1 animals | 2015 | illegal | Hunting/poaching | NA |
| 42 | Cameroon | West Africa | African palm civet | NA*ndinia binotata* | Small mammalian predators | 1 animals + 3 trophies (skin) | 2015 | illegal | Hunting/poaching | NA |
| 42 | Cameroon | West Africa | Mountain reedbuck | *Redunca fulvorufula* | Large non-predatory mammals | 2 trophies | 2015 | illegal | Hunting/poaching | NA |
| 42 | Cameroon | West Africa | Toco toucan | *Ramphastos toco* | NA | 5 trophies (3 feeds) | 2015 | illegal | Hunting/poaching | NA |
| 42 | Cameroon | West Africa | Long-tailed pangolin | *Manis tetradactyla* | NA | 5 animals | 2015 | illegal | Hunting/poaching | NA |
| 42 | Cameroon | West Africa | Sun-tailed monkey | *Cercopithecus solatus* | Small bodied primates | 7 animals | 2015 | illegal | Hunting/poaching | NA |
| 42 | Cameroon | West Africa | Brush-tailed porcupines | *Atherurus africanus* | NA | 4 animals | 2015 | illegal | Hunting/poaching | NA |
| 42 | Cameroon | West Africa | African rock python | *Python sebae* | NA | 7 legs | 2015 | illegal | Hunting/poaching | NA |
| 42 | Cameroon | West Africa | Greater cane rat | *Thryonomys swinderianus* | NA | 1 animals | 2015 | illegal | Hunting/poaching | NA |
| 42 | Cameroon | West Africa | Southern tree hyrax | *Dendrohyrax arboreus* | NA | 3 animals | 2015 | illegal | Hunting/poaching | NA |
| 42 | Cameroon | West Africa | Red-flanked duiker | *Cephalophus rufilatus* | Small to medium non-predatory mammals | 4 legs | 2015 | illegal | Hunting/poaching | NA |
| 42 | Cameroon | West Africa | Red-fronted gazelle | *Eudorcas rufifrons* | NA | 19 legs | 2015 | illegal | Hunting/poaching | NA |
| 42 | Cameroon | West Africa | Water chevrotain | *Hyemoschus aquaticus* | Small to medium non-predatory mammals | 11 fentes | 2015 | illegal | Hunting/poaching | NA |
| 42 | Cameroon | West Africa | Red river hog | *Potamochoerus porcus* | Omnivorous mammals | 16 legs | 2015 | illegal | Hunting/poaching | NA |
| 42 | Cameroon | West Africa | Bushbuck | *Tragelaphus scriptus* | Small to medium non-predatory mammals | 3 animals | 2015 | illegal | Hunting/poaching | NA |
| 42 | Cameroon | West Africa | Sitatunga | *Tragelaphus spekii* | Large non-predatory mammals | 14 fentes | 2015 | illegal | Hunting/poaching | NA |
| 42 | Cameroon | West Africa | Bay Duiker | *Cephalophus dorsalis* | Small to medium non-predatory mammals | 23 animals + 8 fentes | 2015 | illegal | Hunting/poaching | NA |
| 42 | Cameroon | West Africa | Red-flanked duiker | *Cephalophus rufilatus* | Small to medium non-predatory mammals | 13 animals | 2015 | illegal | Hunting/poaching | NA |
| 42 | Cameroon | West Africa | African rock python | *Python sebae* | NA | 1 animals | 2015 | illegal | Hunting/poaching | NA |
| 42 | Cameroon | West Africa | Brush-tailed porcupines | *Atherurus africanus* | NA | 16 animals | 2015 | illegal | Hunting/poaching | NA |
| 42 | Cameroon | West Africa | African palm civet | NA*ndinia binotata* | Small mammalian predators | 2 animals | 2015 | illegal | Hunting/poaching | NA |
| 42 | Cameroon | West Africa | Western tree hyrax | *Dendrohyrax dorsalis* | NA | 4 animals | 2015 | illegal | Hunting/poaching | NA |
| 42 | Cameroon | West Africa | Black-fronted duiker | *Cephalophus nigrifrons* | NA | 6 animals + 8 fuentes | 2015 | illegal | Hunting/poaching | NA |
| 42 | Cameroon | West Africa | Blue duiker | *Philantomba monticola* | Small to medium non-predatory mammals | 9 animals + 21 legs | 2015 | illegal | Hunting/poaching | NA |
| 42 | Cameroon | West Africa | Greater spot-nosed monkey | *Cercopithecus nictitans* | Small bodied primates | 15 animals + 36 fentes | 2015 | illegal | Hunting/poaching | NA |
| 42 | Cameroon | West Africa | Red-eared guenon | *Cercopithecus cephus* | Small bodied primates | 1 animals | 2015 | illegal | Hunting/poaching | NA |
| 42 | Cameroon | West Africa | Long-tailed pangolin | *Manis tetradactyla* | NA | 5 animals | 2015 | illegal | Hunting/poaching | NA |
| 42 | Cameroon | West Africa | Chimpanzee | *Pan troglodytes* | Great apes | 4 legs | 2015 | illegal | Hunting/poaching | NA |
| 42 | Cameroon | West Africa | Cynocéphale | *Cynophalus spp* | NA | 2 fuentes | 2015 | illegal | Hunting/poaching | NA |
| 42 | Cameroon | West Africa | Common genet | *Genetta genetta* | Small mammalian predators | 1 fuente | 2015 | illegal | Hunting/poaching | NA |
| 42 | Cameroon | West Africa | African buffalo | *Syncerus caffer* | Large non-predatory mammals | 2 trophies | 2015 | illegal | Hunting/poaching | NA |
| 42 | Cameroon | West Africa | Long-tailed pangolin | *Manis tetradactyla* | NA | 1 animals | 2015 | illegal | Hunting/poaching | NA |
| 42 | Cameroon | West Africa | Red-fronted Gazelle | *Eudorcas rufifrons* | NA | 35 legs | 2016 | illegal | Hunting/poaching | NA |
| 42 | Cameroon | West Africa | Water chevrotain | *Hyemoschus aquaticus* | Small to medium non-predatory mammals | 11 fentes | 2016 | illegal | Hunting/poaching | NA |
| 42 | Cameroon | West Africa | Bushbuck | *Tragelaphus scriptus* | Small to medium non-predatory mammals | 3 animals | 2016 | illegal | Hunting/poaching | NA |
| 42 | Cameroon | West Africa | Red river hog | *Potamochoerus porcus* | Omnivorous mammals | 26 legs | 2016 | illegal | Hunting/poaching | NA |
| 42 | Cameroon | West Africa | Sitatunga | *Tragelaphus spekii* | Large non-predatory mammals | 14 fentes | 2016 | illegal | Hunting/poaching | NA |
| 42 | Cameroon | West Africa | Bay Duiker | *Cephalophus dorsalis* | Small to medium non-predatory mammals | 23 animals + 15 fentes | 2016 | illegal | Hunting/poaching | NA |
| 42 | Cameroon | West Africa | Red-flanked duiker | *Cephalophus rufilatus* | Small to medium non-predatory mammals | 5 animals | 2016 | illegal | Hunting/poaching | NA |
| 42 | Cameroon | West Africa | African rock python | *Python sebae* | NA | 1 animals | 2016 | illegal | Hunting/poaching | NA |
| 42 | Cameroon | West Africa | Brush-tailed porcupines | *Atherurus africanus* | NA | 25 animals | 2016 | illegal | Hunting/poaching | NA |
| 42 | Cameroon | West Africa | African palm civet | NA*ndinia binotata* | Small mammalian predators | 2 animals | 2016 | illegal | Hunting/poaching | NA |
| 42 | Cameroon | West Africa | Western tree hyrax | *Dendrohyrax dorsalis* | NA | 4 animals | 2016 | illegal | Hunting/poaching | NA |
| 42 | Cameroon | West Africa | Black-fronted Duiker | *Cephalophus nigrifrons* | NA | 5 animals | 2016 | illegal | Hunting/poaching | NA |
| 42 | Cameroon | West Africa | Blue duiker | *Philantomba monticola* | Small to medium non-predatory mammals | 34 legs + 1 animals | 2016 | illegal | Hunting/poaching | NA |
| 42 | Cameroon | West Africa | Greater spot-nosed monkey | *Cercopithecus nictitans* | Small bodied primates | 15 animals | 2016 | illegal | Hunting/poaching | NA |
| 42 | Cameroon | West Africa | Moustached Monkey | *Cercopithecus cephus* | Small bodied primates | 2 animals | 2016 | illegal | Hunting/poaching | NA |
| 42 | Cameroon | West Africa | Long-tailed pangolin | *Manis tetradactyla* | NA | 5 animals | 2016 | illegal | Hunting/poaching | NA |
| 42 | Cameroon | West Africa | Giant Ground Pangolin | *Smutsia gigantea* | Insectivorous mammals | 1 animals + 23.5 kg scale | 2016 | illegal | Hunting/poaching | NA |
| 42 | Cameroon | West Africa | Sun-tailed monkey | *Cercopithecus solatus* | Small bodied primates | 4 legs | 2016 | illegal | Hunting/poaching | NA |
| 42 | Cameroon | West Africa | Common genet | *Genetta genetta* | Small mammalian predators | 1 fentes | 2016 | illegal | Hunting/poaching | NA |
| 42 | Cameroon | West Africa | African buffalo | *Syncerus caffer* | Large non-predatory mammals | 2 trophies | 2016 | illegal | Hunting/poaching | NA |
| 42 | Cameroon | West Africa | Brush-tailed porcupines | *Atherurus africanus* | NA | 2 animals | 2016 | illegal | Hunting/poaching | NA |
| 42 | Cameroon | West Africa | Black-fronted Duiker | *Cephalophus nigrifrons* | NA | 1 animals + 8 fentes | 2016 | illegal | Hunting/poaching | NA |
| 42 | Cameroon | West Africa | Blue duiker | *Philantomba monticola* | Small to medium non-predatory mammals | 7 animals + 1 fente | 2016 | illegal | Hunting/poaching | NA |
| 42 | Cameroon | West Africa | Greater spot-nosed monkey | *Cercopithecus nictitans* | Small bodied primates | 18 fentes | 2016 | illegal | Hunting/poaching | NA |
| 42 | Cameroon | West Africa | Giant Ground Pangolin | *Smutsia gigantea* | Insectivorous mammals | 4 kg scales | 2016 | illegal | Hunting/poaching | NA |
| 42 | Cameroon | West Africa | Red river hog | *Potamochoerus porcus* | Omnivorous mammals | 4 fentes | 2016 | illegal | Hunting/poaching | NA |
| 42 | Cameroon | West Africa | Red-eared guenon | *Cercopithecus cephus* | Small bodied primates | 28 animals | 2016 | illegal | Hunting/poaching | NA |
| 42 | Cameroon | West Africa | Brush-tailed porcupines | *Atherurus africanus* | NA | 17 animals | 2016 | illegal | Hunting/poaching | NA |
| 42 | Cameroon | West Africa | Black-fronted duiker | *Cephalophus nigrifrons* | NA | 12 fentes | 2016 | illegal | Hunting/poaching | NA |
| 42 | Cameroon | West Africa | Blue duiker | *Philantomba monticola* | Small to medium non-predatory mammals | 32 animals | 2016 | illegal | Hunting/poaching | NA |
| 42 | Cameroon | West Africa | Long-tailed pangolin | *Manis tetradactyla* | NA | 8 gigets | 2017 | illegal | Hunting/poaching | NA |
| 42 | Cameroon | West Africa | African buffalo | *Syncerus caffer* | Large non-predatory mammals | 21 legs + 12 trophies | 2017 | illegal | Hunting/poaching | NA |
| 42 | Cameroon | West Africa | Red river hog | *Potamochoerus porcus* | Omnivorous mammals | 2 animals + 45 legs | 2017 | illegal | Hunting/poaching | NA |
| 42 | Cameroon | West Africa | Sitatunga | *Tragelaphus spekii* | Large non-predatory mammals | 6 animals + 9 legs | 2017 | illegal | Hunting/poaching | NA |
| 42 | Cameroon | West Africa | Bay Duiker | *Cephalophus dorsalis* | Small to medium non-predatory mammals | 13 animals +94 legs+12 trophies (6 skins) | 2017 | illegal | Hunting/poaching | NA |
| 42 | Cameroon | West Africa | Blue duiker | *Philantomba monticola* | Small to medium non-predatory mammals | 38 animals + 25 legs +138 legs | 2017 | illegal | Hunting/poaching | NA |
| 42 | Cameroon | West Africa | Cynocéphale | *Cynophalus spp* | NA | 2 animals | 2017 | illegal | Hunting/poaching | NA |
| 42 | Cameroon | West Africa | Bongo | *Tragelaphus eurycerus.* | NA | 16 legs | 2017 | illegal | Hunting/poaching | NA |
| 42 | Cameroon | West Africa | Water chevrotain | *Hyemoschus aquaticus* | Small to medium non-predatory mammals | 5 legs+6 trophies (skin) | 2017 | illegal | Hunting/poaching | NA |
| 42 | Cameroon | West Africa | Chimpanzee | *Pan troglodytes* | Great apes | 3 animals | 2017 | illegal | Hunting/poaching | NA |
| 42 | Cameroon | West Africa | Gambian pouched rat | *Cricetomys gambianus* | NA | 1 animals | 2017 | illegal | Hunting/poaching | NA |
| 42 | Cameroon | West Africa | African palm civet | NA*ndinia binotata* | Small mammalian predators | 1 animals + 3 | 2017 | illegal | Hunting/poaching | NA |
| 42 | Cameroon | West Africa | Mountain reedbuck | *Redunca fulvorufula* | Large non-predatory mammals | 2 trophies | 2017 | illegal | Hunting/poaching | NA |
| 42 | Cameroon | West Africa | Toco toucan | *Ramphastos toco* | NA | 5 trophies (3 legs) | 2017 | illegal | Hunting/poaching | NA |
| 42 | Cameroon | West Africa | Long-tailed pangolin | *Manis tetradactyla* | NA | 5 animals + trophies (scales) | 2017 | illegal | Hunting/poaching | NA |
| 42 | Cameroon | West Africa | Sun-tailed monkey | *Cercopithecus solatus* | Small bodied primates | 7 animals | 2017 | illegal | Hunting/poaching | NA |
| 42 | Cameroon | West Africa | Brush-tailed porcupines | *Atherurus africanus* | NA | 4 animals | 2017 | illegal | Hunting/poaching | NA |
| 42 | Cameroon | West Africa | African rock python | *Python sebae* | NA | 7 legs | 2017 | illegal | Hunting/poaching | NA |
| 42 | Cameroon | West Africa | Greater cane rat | *Thryonomys swinderianus* | NA | 1 animals | 2017 | illegal | Hunting/poaching | NA |
| 42 | Cameroon | West Africa | Western tree hyrax | *Dendrohyrax dorsalis* | NA | 3 animals | 2017 | illegal | Hunting/poaching | NA |
| 42 | Cameroon | West Africa | Red-flanked duiker | *Cephalophus rufilatus* | Small to medium non-predatory mammals | 4 legs | 2017 | illegal | Hunting/poaching | NA |
| 42 | Cameroon | West Africa | Giant Ground Pangolin | *Smutsia gigantea* | Insectivorous mammals | 5 legs | 2018 | illegal | Hunting/poaching | NA |
| 42 | Cameroon | West Africa | African buffalo | *Syncerus caffer* | Large non-predatory mammals | 21 legs + 12 trophies | 2018 | illegal | Hunting/poaching | NA |
| 42 | Cameroon | West Africa | Red river hog | *Potamochoerus porcus* | Omnivorous mammals | 1 animals + 37 legs | 2018 | illegal | Hunting/poaching | NA |
| 42 | Cameroon | West Africa | Sitatunga | *Tragelaphus spekii* | Large non-predatory mammals | 1 animals + 4 legs | 2018 | illegal | Hunting/poaching | NA |
| 42 | Cameroon | West Africa | Bay Duiker | *Cephalophus dorsalis* | Small to medium non-predatory mammals | 8 animals + 75 legs + 6 skins | 2018 | illegal | Hunting/poaching | NA |
| 42 | Cameroon | West Africa | Blue duiker | *Philantomba monticola* | Small to medium non-predatory mammals | 12 animals + 12 legs + 138 trophies | 2018 | illegal | Hunting/poaching | NA |
| 42 | Cameroon | West Africa | Cynocéphale | *Cynophalus spp* | NA | 2 animals | 2018 | illegal | Hunting/poaching | NA |
| 42 | Cameroon | West Africa | Water chevrotain | *Hyemoschus aquaticus* | Small to medium non-predatory mammals | 5 legs + 6 skins | 2018 | illegal | Hunting/poaching | NA |
| 42 | Cameroon | West Africa | Chimpanzee | *Pan troglodytes* | Great apes | 3 animals | 2018 | illegal | Hunting/poaching | NA |
| 42 | Cameroon | West Africa | Gambian pouched rat | *Cricetomys gambianus* | NA | 1 animals | 2018 | illegal | Hunting/poaching | NA |
| 42 | Cameroon | West Africa | African palm civet | NA*ndinia binotata* | Small mammalian predators | 3 skin | 2018 | illegal | Hunting/poaching | NA |
| 42 | Cameroon | West Africa | Mountain reedbuck | *Redunca fulvorufula* | Large non-predatory mammals | 2 skin | 2018 | illegal | Hunting/poaching | NA |
| 42 | Cameroon | West Africa | Toco toucan | *Ramphastos toco* | NA | 5 trophies + 3 legs | 2018 | illegal | Hunting/poaching | NA |
| 42 | Cameroon | West Africa | Long-tailed pangolin | *Manis tetradactyla* | NA | scales | 2018 | illegal | Hunting/poaching | NA |
| 42 | Cameroon | West Africa | Sun-tailed monkey | *Cercopithecus solatus* | Small bodied primates | 7 animals | 2018 | illegal | Hunting/poaching | NA |
| 42 | Cameroon | West Africa | Brush-tailed porcupines | *Atherurus africanus* | NA | 2 animals | 2018 | illegal | Hunting/poaching | NA |
| 42 | Cameroon | West Africa | African rock python | *Python sebae* | NA | 3 legs | 2018 | illegal | Hunting/poaching | NA |
| 42 | Cameroon | West Africa | Animals | NA | NA | 0 | per year | legal | population control | NA |
| 43 | Cameroon | West Africa | Animals | NA | NA | 0 | per year | legal | population control | NA |
| 43 | Cameroon | West Africa | Animals | NA | NA | 0 | per year | legal | population control | NA |
| 44 | Cameroon | West Africa | Animals | NA | NA | 0 | per year | legal | population control | NA |
| 45 | Cameroon | West Africa | Animals | NA | NA | 0 | per year | legal | population control | NA |
| 46 | Cameroon | West Africa | Animals | NA | NA | 0 | per year | legal | population control | NA |
| 47 | Cameroon | West Africa | Animals | NA | NA | 0 | per year | legal | NA | NA |
| 47 | Cameroon | West Africa | Bohor Reedbuck | *Redunca redunca* | NA | 6 | 2017 | illegal | NA | NA |
| 47 | Cameroon | West Africa | Giant Eland | *Tragelaphus derbianus* | NA | 2 | 2017 | illegal | NA | NA |
| 47 | Cameroon | West Africa | Kob | *Kobus kob* | Small to medium non-predatory mammals | 5 | 2017 | illegal | NA | NA |
| 47 | Cameroon | West Africa | Hartebeest | *Alcelaphus buselaphus* | Large non-predatory mammals | 5 | 2017 | illegal | NA | NA |
| 47 | Cameroon | West Africa | waterbuck | *Kobus ellipsiprymnus* | Large non-predatory mammals | 2 | 2017 | illegal | NA | NA |
| 47 | Cameroon | West Africa | Red river hog | *Potamochoerus porcus* | Omnivorous mammals | 1 | 2017 | illegal | NA | NA |
| 47 | Cameroon | West Africa | Bushbuck | *Tragelaphus scriptus* | Small to medium non-predatory mammals | 2 | 2017 | illegal | NA | NA |
| 47 | Cameroon | West Africa | Greater cane rat | *Thryonomys swinderianus* | NA | 2 | 2017 | illegal | NA | NA |
| 47 | Cameroon | West Africa | birds | *Aves* | NA | 100 | 2017 | illegal | NA | NA |
| 47 | Cameroon | West Africa | Oribi | *Ourebia ourebi* | Small to medium non-predatory mammals | 1 | 2017 | illegal | NA | NA |
| 47 | Cameroon | West Africa | Savannah Monitor | *Varanus exanthematicus* | NA | 1 | 2017 | illegal | NA | NA |
| 47 | Cameroon | West Africa | Crested Porcupine | *Hystrix cristata* | NA | 2 | 2017 | illegal | NA | NA |
| 47 | Cameroon | West Africa | sable_antelope | *Hippotragus niger* | Large non-predatory mammals | 4 | 2017 | illegal | NA | NA |
| 47 | Cameroon | West Africa | Elephant | *Loxodonta spec.* | Large non-predatory mammals | 4 | 2017 | illegal | NA | NA |
| 47 | Cameroon | West Africa | hyena | *Crocuta crocuta* | Top mammalian predators | 1 | 2017 | illegal | NA | NA |
| 47 | Cameroon | West Africa | Crested Porcupine | *Hystrix cristata* | NA | 2 | 2017 | illegal | NA | NA |
| 47 | Cameroon | West Africa | Hartebeest | *Alcelaphus buselaphus* | Large non-predatory mammals | 8 | 2016 | illegal | NA | NA |
| 47 | Cameroon | West Africa | sable_antelope | *Hippotragus niger* | Large non-predatory mammals | 4 | 2016 | illegal | NA | NA |
| 47 | Cameroon | West Africa | Bohor Reedbuck | *Redunca redunca* | NA | 7 | 2016 | illegal | NA | NA |
| 47 | Cameroon | West Africa | Oribi | *Ourebia ourebi* | Small to medium non-predatory mammals | 8 | 2016 | illegal | NA | NA |
| 47 | Cameroon | West Africa | Crested Porcupine | *Hystrix cristata* | NA | 2 | 2016 | illegal | NA | NA |
| 47 | Cameroon | West Africa | Kob | *Kobus kob* | Small to medium non-predatory mammals | 4 | 2016 | illegal | NA | NA |
| 47 | Cameroon | West Africa | Red river hog | *Potamochoerus porcus* | Omnivorous mammals | 2 | 2016 | illegal | NA | NA |
| 47 | Cameroon | West Africa | Bushbuck | *Tragelaphus scriptus* | Small to medium non-predatory mammals | 1 | 2016 | illegal | NA | NA |
| 48 | Cameroon | West Africa | Elephant | *Loxodonta spec.* | Large non-predatory mammals | 1 | 2013 | illegal | Hunting/poaching | NA |
| 48 | Cameroon | West Africa | Elephant | *Loxodonta spec.* | Large non-predatory mammals | 1 | 2014 | illegal | Hunting/poaching | NA |
| 48 | Cameroon | West Africa | Elephant | *Loxodonta spec.* | Large non-predatory mammals | 1 | 2017 | illegal | Hunting/poaching | NA |
| 48 | Cameroon | West Africa | Chimpanzee | *Pan troglodytes* | Great apes | 1 | 2013 | illegal | Hunting/poaching | NA |
| 48 | Cameroon | West Africa | Animals | NA | NA | 0 | per year | legal | population control | NA |
| 49 | Cameroon | West Africa | Animals | NA | NA | 0 | per year | legal | population control | NA |
| 50 | Guinea | West Africa | Animals | NA | NA | 0 | per year | legal | population control | NA |
| 51 | Guinea | West Africa | Animals | NA | NA | 0 | per year | legal | population control | NA |
| 52 | Guinea | West Africa | Animals | NA | NA | 0 | per year | legal | population control | NA |
| 53 | Guinea | West Africa | Animals | NA | NA | 0 | per year | legal | population control | NA |
| 54 | Romania | East Europe | Animals | NA | NA | 0 | NA | illegal | NA | NA |
| 55 | Romania | East Europe | Animals | NA | NA | 0 | per year | legal | population control | NA |
| 56 | Romania | East Europe | Animals | NA | NA | 0 | per year | legal | population control | NA |
| 57 | Romania | East Europe | wolf | *Canis lupus* | Top mammalian predators | 1 | in 10 years | legal | Avoiding more damages (the wolf was constantly attacking a sheep station) | NA |
| 58 | Romania | East Europe | Animals | NA | NA | 0 | per year | legal | population control | NA |
| 59 | Spain | West Europe | Animals | NA | NA | 0 | per year | legal | population control | NA |
| 60 | Spain | West Europe | Animals | NA | NA | 0 | NA | illegal | NA | NA |
| 61 | Spain | West Europe | lynx | *Lynx lynx* | Top mammalian predators | 5 | 2017 | illegal | NA | NA |
| 61 | Spain | West Europe | Wild pig | *Sus scrofa* | Omnivorous mammals | 300 | per year | legal | NA | NA |
| 62 | Spain | West Europe | animals | NA | NA | 0 | NA | legal | NA | NA |
| 63 | Spain | West Europe | Red deer | *Cervus elaphus* | Large non-predatory mammals | 1150 killed/450 sold | NA | legal | NA | NA |
| 64 | Poland | East Europe | Wild pig | *Sus scrofa* | Omnivorous mammals | 43 | per year | legal | Prevention African Swine Fewer | NA |
| 65 | Poland | East Europe | Golden_eagle | *Aquila chrysaetos* | raptors | 4 | last 10 years | illegal | Hunting/poaching | NA |
| 65 | Poland | East Europe | wolf | *Canis lupus* | Top mammalian predators | 1 | last 10 years | illegal | Hunting/poaching | NA |
| 65 | Poland | East Europe | wolf | *Canis lupus* | Top mammalian predators | 1 | last 10 years | illegal | Hunting/poaching | NA |
| 65 | Poland | East Europe | Red deer | *Cervus elaphus* | Large non-predatory mammals | 10 | per year | legal | population control | NA |
| 65 | Poland | East Europe | Wild pig | *Sus scrofa* | Omnivorous mammals | 10 | per year | legal | Disease control | NA |
| 66 | Poland | East Europe | red_fox | *Vulpes vulpes* | Small mammalian predators | 13 | per year | legal | population control | NA |
| 66 | Poland | East Europe | Red deer | *Cervus elaphus* | Large non-predatory mammals | 4 | per year | legal | population control | NA |
| 66 | Poland | East Europe | Roe deer | *Capreolus capreolus* | Small to medum non-predatory mammals | 13 | per year | legal | population control | NA |
| 66 | Poland | East Europe | Wild pig | *Sus scrofa* | Omnivorous mammals | 96 | per year | legal | Disease control | NA |
| 67 | Poland | East Europe | Wild pig | *Sus scrofa* | Omnivorous mammals | 16 | per year | legal | NA | NA |
| 68 | Poland | East Europe | animals | NA | NA | 0 | NA | legal | NA | NA |
| 69 | Czech republic | East Europe | ungulates, mouflon | NA | NA | 200-600 | per year | legal | population control | NA |
| 70 | Czech republic | East Europe | Red deer | *Cervus elaphus* | Large non-predatory mammals | 1000 | per year | legal | population control | NA |
| 70 | Czech republic | East Europe | Roe deer | *Capreolus capreolus* | Small to medium non-predatory mammals | 200 | per year | legal | population control | NA |
| 70 | Czech republic | East Europe | Wild pig | *Sus scrofa* | Omnivorous mammals | 400 | per year | legal | population control | NA |
| 70 | Czech republic | East Europe | red_fox | *Vulpes vulpes* | Small mammalian predators | 100 | per year | legal | population control | NA |
| 71 | Germany | West Europe | lynx | *Lynx lynx* | Top mammalian predators | 15 | per year | illegal | human-wildlife-conflict | NA |
| 71 | Germany | West Europe | Red deer | *Cervus elaphus* | Large non-predatory mammals | 130-140 | per year | legal | population control | NA |
| 71 | Germany | West Europe | Wild pig | *Sus scrofa* | Omnivorous mammals | 100-300 | per year | legal | population control | NA |
| 71 | Germany | West Europe | Roe deer | *Capreolus capreolus* | Small to medium non-predatory mammals | 130-140 | per year | legal | population control | NA |
| 72 | Poland | East Europe | European bison | *Bison bo*NA*sus* | Large non-predatory mammals | 4 | last 10 years | illegal | Hunting/poaching | NA |
| 72 | Poland | East Europe | animals | NA | NA | 0 | NA | legal | NA | NA |
| 73 | Gabon | West Africa | Animals | NA | NA | 0 | per year | legal | NA | NA |
| 74 | Gabon | West Africa | Animals | NA | NA | 0 | per year | legal | NA | NA |
| 75 | Gabon | West Africa | Animals | NA | NA | 0 | per year | legal | NA | NA |
| 76 | Gabon | West Africa | Animals | NA | NA | 0 | per year | legal | NA | NA |
| 77 | France | West Europe | Animals | NA | NA | 0 | NA | illegal | NA | NA |
| 77 | France | West Europe | Animals | NA | NA | 0 | per year | legal | l | NA |
| 78 | France | West Europe | Animals | NA | NA | 0 | 2017 | illegal | NA | NA |
| 78 | France | West Europe | Animals | NA | NA | 0 | per year | legal | NA | NA |
| 79 | France | West Europe | Animals | NA | NA | 0 | NA | illegal | NA | NA |
| 79 | France | West Europe | wild pig | *Sus scrofa* | Omnivorous mammals | 30 | per year | legal | population control | shooting |
| 80 | France | West Europe | birds of prey | NA | NA | 1.5 | per per year | illegal | NA | NA |
| 80 | France | West Europe | Wild porc | *Sus scrofa* | NA | 7104.4 | 2017-2018 | legal | Agro-sylvo-cynegetic equilibrium (L425-4 with obligation of result in core area) | NA |
| 80 | France | West Europe | Deer | *Cervus* | NA | 510.1 | 2017-2018 | legal | Agro-sylvo-cynegetic equilibrium (L425-4 with obligation of result in core area) | NA |
| 81 | Germany | West Europe | Animals | NA | NA | 0 | per year | illegal | NA | NA |
| 81 | Germany | West Europe | Wild pig | *Sus scrofa* | Omnivorous mammals | 200 | per year | legal | Avoid damages for neighbours to increase acceptance of park | NA |
| 81 | Germany | West Europe | Deer | *Capreolus capreolus* | Small to medium non-predatory mammals | 50 | per year | legal | Avoid damages for neighbours to increase acceptance of park | NA |
| 81 | Germany | West Europe | Fallow deer | *Dama dama* | Small to medium non-predatory mammals | 40 | per year | legal | Avoid damages for neighbours to increase acceptance of park | NA |
| 81 | Germany | West Europe | Red deer | *Cervus elaphus* | Large non-predatory mammals | 10 | per year | legal | Avoid damages for neighbours to increase acceptance of park | NA |
| 82 | Germany | West Europe | Animals | Animals | NA | 0 | NA | illegal | NA | NA |
| 82 | Germany | West Europe | Animals | Animals | NA | 0 | NA | legal | NA | NA |
| 83 | Congo | Central Africa | Animals | NA | NA | 0 | NA | legal | NA | NA |
| 84 | Congo | Central Africa | Animals | NA | NA | 0 | NA | legal | NA | NA |
| 85 | Congo | Central Africa | Animals | NA | NA | 0 | NA | legal | NA | NA |
| 86 | Namibia | South Africa | Animals | NA | NA | 0 | NA | illegal | NA | NA |
| 86 | Namibia | South Africa | Animals | NA | NA | 0 | NA | legal | NA | NA |
| 87 | Namibia | South Africa | antelopes | NA | NA | 4 | per year | illegal | NA | NA |
| 87 | Namibia | South Africa | Animals | NA | NA | 0 | per year | legal | NA | NA |
| 88 | Slovakia | East Europe | Red deer | *Cervus elaphus* | Large non-predatory mammals | 600 | per year | legal | Population control | NA |
| 88 | Slovakia | East Europe | Wild pig | *Sus scrofa* | Omnivorous mammals | 600 | per year | legal | Population control | NA |
| 89 | Slovakia | East Europe | Animals | NA | NA | 0 | per year | legal | Population control | NA |
| 90 | Slovakia | East Europe | Red deer | *Cervus elaphus* | Large non-predatory mammals | 50 | per year | legal | NA | NA |
| 90 | Slovakia | East Europe | Wild pig | *Sus scrofa* | Omnivorous mammals | 70 | per year | legal | NA | NA |
| 91 | Slovakia | East Europe | Animals | NA | NA | 0 | per year | legal | NA | NA |
| 92 | Chad | West Africa | Animals | NA | NA | 0 | per year | legal | population control | NA |
| 93 | Chad | West Africa | Animals | NA | NA | 0 | per year | legal | population control | NA |
| 94 | Chad | West Africa | Animals | NA | NA | 0 | per year | legal | population control | NA |
| 95 | Chad | West Africa | Animals | NA | NA | 0 | per year | legal | population control | NA |
| 96 | CAR | Central Africa | Animals | NA | NA | 0 | per year | legal | population control | NA |
| 97 | CAR | Central Africa | Animals | NA | NA | 0 | per year | legal | population control | NA |
| 98 | South Africa | South African | NA | NA | NA | 0 | per year | legal | population control | NA |
| 99 | South Africa | South African | Animals | NA | NA | 0 | NA | illegal | NA | NA |
| 99 | South Africa | South African | Animals | NA | NA | 0 | NA | legal | NA | NA |
| 100 | South Africa | South African | Animals | NA | NA | 0 | NA | legal | NA | NA |
| 101 | South Africa | South African | Animals | NA | NA | 0 | NA | legal | NA | NA |
| 102 | South Africa | South African | Animals | NA | NA | 0 | NA | illegal | NA | NA |
| 103 | Sweden | North Europe | Carnivores | NA | Top mammalian predators | 0 | NA | legal | Damage control of all carnivores/ Reducing carnivores for reindeer husbandry | NA |
| 104 | Sweden | North Europe | Carnivores | NA | Top mammalian predators | 1 | NA | legal | Damage control of all carnivores/ Reducing carnivores for reindeer husbandry | NA |
| 105 | Sweden | North Europe | Carnivores | NA | Top mammalian predators | 6 | NA | legal | Damage control of all carnivores/ Reducing carnivores for reindeer husbandry | NA |
| 106 | Sweden | North Europe | Carnivores | NA | Top mammalian predators | 0 | NA | legal | Damage control of all carnivores/ Reducing carnivores for reindeer husbandry | NA |
| 107 | Sweden | North Europe | Carnivores | NA | Top mammalian predators | 0 | NA | legal | Damage control of all carnivores/ Reducing carnivores for reindeer husbandry | NA |
| 108 | Sweden | North Europe | Carnivores | NA | Top mammalian predators | 3 | NA | legal | Damage control of all carnivores/ Reducing carnivores for reindeer husbandry | NA |

*Table V Preparation and authorization of quotas*

| **PA** | **Country** | **Continent** | **Determination culling quotas** | **Authorization** |
| --- | --- | --- | --- | --- |
| 1 | Austria | Europe | Regulations of hunting associations | Ministry |
| 2 | Austria | Europe | NA | NA |
| 3 | Austria | Europe | Hunting associations | Ministry |
| 4 | Czech Republic | Europe | NA | NA |
| 5 | Czech Republic | Europe | NA | NA |
| 6 | Czech Republic | Europe | National park administration | National park administration |
| 7 | Czech Republic | Europe | NA | NA |
| 8 | France | Europe | Government | Government |
| 9 | France | Europe | NA | NA |
| 10 | France | Europe | NA | NA |
| 11 | France | Europe | NA | NA |
| 12 | Germany | Europe | NA | NA |
| 13 | Germany | Europe | National park administration | National park administration |
| 14 | Germany | Europe | National park administration | Government |
| 15 | Germany | Europe | NA | NA |
| 16 | Hungary | Europe | NA | NA |
| 17 | Hungary | Europe | Hunting associations | NA |
| 18 | Hungary | Europe | Hunting associations | NA |
| 19 | Hungary | Europe | Hunting associations | NA |
| 20 | Hungary | Europe | Hunting associations | NA |
| 21 | Poland | Europe | National park administration (management plan) | Ministry of environment |
| 22 | Poland | Europe | National park administration (management plan) | Ministry of environment |
| 23 | Poland | Europe | National park administration (management plan) | Ministry of environment |
| 24 | Poland | Europe | National park administration (management plan) | Ministry of environment |
| 25 | Poland | Europe | National park administration (management plan) | Ministry of environment |
| 26 | Poland | Europe | National park administration (management plan) | Ministry of environment |
| 27 | Romania | Europe | No regular culling | No culling |
| 28 | Romania | Europe | No regular culling | No culling |
| 29 | Romania | Europe | No regular culling | No culling |
| 30 | Romania | Europe | No regular culling | No culling |
| 31 | Romania | Europe | No regular culling | No culling |
| 32 | Romania | Europe | No regular culling | No culling |
| 33 | Slovakia | Europe | Hunting association | Ministry of agriculture |
| 34 | Slovakia | Europe | Hunting associations | Ministry of agriculture |
| 35 | Slovakia | Europe | Hunting associations | Ministry of agriculture |
| 36 | Slovakia | Europe | Hunting associations | Ministry of agriculture |
| 37 | Slovakia | Europe | Hunting associations | Ministry of agriculture |
| 38 | Spain | Europe | NA | NA |
| 39 | Spain | Europe | NA | NA |
| 40 | Spain | Europe | NA | NA |
| 41 | Spain | Europe | NA | NA |
| 42 | Spain | Europe | NA | NA |
| 43 | Sweden | Europe | NA | NA |
| 44 | Sweden | Europe | Government | Government |
| 45 | Sweden | Europe | Government | Government |
| 46 | Sweden | Europe | Government | Government |
| 47 | Sweden | Europe | Government | Government |
| 48 | Sweden | Europe | NA | NA |

**4. Cross-validation of data**

We cross-checked the validity of our data on mammals and birds abundance change with data collated from the Living Planet Database (LPD) [25], IUCN SSC A.P.E.S database [26] as well as published and unpublished reports (Table 22 in S1 Appendix). We calculated the average change in the abundance of 104 species of mammals and birds. When we compared the rate of change reported by the PA managers and the other sources, we found overlap in 82.4 % of the cases (Table 22 in S1 Appendix). Furthermore, we conducted an online survey with Non-Governmental Organizations working at the surveyed PAs containing the same questions related to socio-economic conditions. The online survey was translated into eight languages and distributed to various global and local operating NGOs, however, responses were low (22 respondents, 20 parks). Nevertheless, we cross-validate both datasets. We have grouped the variables similar to our analyses into two levels (declined or stable/improved and low, high). The overlap ranged between 38% and 90% per question and had an average of 60.04 % ± 12.27%. We further compared our data with the Protected Area Management Effectiveness (PAME) assessments [27]. The PAME data were collected on average in 2011 +- 1.4. We used due to different scales a binary scale for comparison. Unfortunately, the hunting variable from the PAME assessment only included one variable combining all types of hunting (legal, illegal) and HWCs, while our variables were differentiated. The evaluation with our hunting and HWC variable revealed here for both 48% overlap (PAME Qu. 5, our questionnaire: Qu. 35, threat assessment: hunting, killing because of human-wildlife conflicts). The variables related to involvement (PAME: Qu. 24 a, our questionnaire: VI Involvement, Qu 1) revealed 61% overlap, willingness of communities to involve in park protection revealed 75% overlap (PAME, Qu. 24c, Our questionnaire VI Involvement, Qu 5) and trust 42% (PAME: Qu. 24a), our questionnaire Index out VI (Involvement), Qu 3,VII, Qu 5). Notably, for the trust assessment, the questions were slightly different as the PAME assessment includes aspects of open communication and trust. We further searched the literature and identified three potential biases through erroneous ratings. First, systematic over-or underrating [28,29], which would bias the intercepts in our models. Second, tending towards the mean by overreporting low levels and underreporting high levels [30], which would decrease the steepness of the estimated effects and reduce the power of our analyses. Third, random errors because of imprecise memories or assessments, which would reduce the power to detect effects. To summarize, in all three cases, we would expect a higher likelihood of false-negative results but not of false-positive results.

*Table W***:** The overlap between data from this study and data from LPD, IUCN SSC A.P.E.S, and other sources.

| **ID** | **Common Name** | **PA** | **Country list** | **Change abundance from this study** | **Change in abundance from other sources** | **Overlap** | **Source** |  |
| --- | --- | --- | --- | --- | --- | --- | --- | --- |
| 1 | Ethiopian wolf | Bale Mountain National Park | Ethiopia | 0 | 0 | Yes | LPD |  |
| 2 | Mountain gorilla / Eastern lowland gorilla | Bwindi Impenetrable National Park | Uganda | 1 | 1 | Yes | LPD |  |
| 3 | Olive baboon | Kibale National Park | Uganda | 1 | 1 | Yes | LPD |  |
| 4 | Red-tailed monkey | Kibale National Park | Uganda | 1 | 0 | No | LPD |  |
| 5 | Chimpanzee | Kibale National Park | Uganda | 1 | 0 | No | LPD |  |
| 6 | African elephant | Kibale National Park | Uganda | 1 | 1 | Yes | LPD |  |
| 7 | Bushpig | Kibale National Park | Uganda | 0 | 1 | No | LPD |  |
| 8 | Topi / Tsessebe antelope | Queen Elizabeth National Park | Uganda | 1 | 1 | Yes | LPD |  |
| 9 | Hippopotamus | Queen Elizabeth National Park | Uganda | 1 | 1 | Yes | LPD |  |
| 10 | African buffalo / Cape buffalo | Queen Elizabeth National Park | Uganda | 1 | 1 | Yes | LPD |  |
| 11 | African elephant | Queen Elizabeth National Park | Uganda | 1 | 1 | Yes | LPD |  |
| 12 | Waterbuck | Queen Elizabeth National Park | Uganda | 1 | 0 | No | LPD |  |
| 13 | Common warthog / Desert warthog | Queen Elizabeth National Park | Uganda | 1 | 0 | No | LPD |  |
| 14 | African buffalo / Cape buffalo | Toro Semliki Wildlife Reserve | Uganda | 1 | 1 | Yes | LPD |  |
| 15 | African elephant | Toro Semliki Wildlife Reserve | Uganda | 1 | 0 | No | LPD |  |
| 16 | Waterbuck | Toro Semliki Wildlife Reserve | Uganda | 1 | 1 | Yes | LPD |  |
| 17 | Common warthog / Desert warthog | Toro Semliki Wildlife Reserve | Uganda | 0 | 1 | No | LPD |  |
| 18 | Bushbuck | Toro Semliki Wildlife Reserve | Uganda | 1 | 1 | Yes | LPD |  |
| 19 | Hippopotamus | Lake Mburo National Park | Uganda | 1 | 1 | Yes | LPD |  |
| 20 | African buffalo / Cape buffalo | Lake Mburo National Park | Uganda | 1 | 1 | Yes | LPD |  |
| 21 | Waterbuck | Lake Mburo National Park | Uganda | 1 | 1 | Yes | LPD |  |
| 22 | Common warthog / Desert warthog | Lake Mburo National Park | Uganda | 0 | 1 | No | LPD |  |
| 23 | Common crane | Hortobágy National Park | Hungary | 1 | 1 | Yes | LPD |  |
| 24 | Great bustard | Hortobágy National Park | Hungary | 1 | 1 | Yes | LPD |  |
| 25 | Black rhinoceros | Nairobi National Park | Kenya | 1 | 1 | Yes | LPD |  |
| 26 | African elephant | Masai Mara National Reserve | Kenya | 1 | 0 | No | LPD |  |
| 27 | spotted hyena | Nechsar National Park | Ethiopia | 1 | 1 | Yes | Unpublished PA report |  |
| 28 | Lion | Nechsar National Park | Ethiopia | 1 | 1 | Yes | Unpublished PA report |  |
| 29 | Leopard | Nechsar National Park | Ethiopia | 1 | 1 | Yes | Unpublished PA report |  |
| 30 | Jacal | Nechsar National Park | Ethiopia | 1 | 1 | Yes | Unpublished PA report |  |
| 31 | wild cat | Nechsar National Park | Ethiopia | 1 | 1 | Yes | Unpublished PA report |  |
| 32 | Zibra | Nechsar National Park | Ethiopia | 1 | 1 | Yes | Unpublished PA report |  |
| 33 | Greater kudu | Nechsar National Park | Ethiopia | 1 | 1 | Yes | Unpublished PA report |  |
| 34 | Lesser kudu | Nechsar National Park | Ethiopia | 1 | 1 | Yes | Unpublished PA report |  |
| 35 | Waterbuck | Nechsar National Park | Ethiopia | 0 | 1 | No | Unpublished PA report |  |
| 36 | Bushbuck | Nechsar National Park | Ethiopia | 1 | 1 | Yes | Unpublished PA report |  |
| 37 | Common duiker | Nechsar National Park | Ethiopia | 1 | 1 | Yes | Unpublished PA report |  |
| 38 | Common warthog / Desert warthog | Nechsar National Park | Ethiopia | 1 | 1 | Yes | Unpublished PA report |  |
| 39 | Baboon | Nechsar National Park | Ethiopia | 1 | 1 | Yes | Unpublished PA report |  |
| 40 | Colombus monkey | Nechsar National Park | Ethiopia | 1 | 1 | Yes | Unpublished PA report |  |
| 41 | Vervent monkey | Nechsar National Park | Ethiopia | 0 | 0 | Yes | Unpublished PA report |  |
| 42 | Chimpanzee | Sapo National Park | Liberia | 1 | 1 | Yes | LPD |  |
| 43 | Chimpanzee | Dja Biosphere Reserve | Cameroon | 1 | 1 | Yes | IUCN SSC A.P.E.S |  |
| 44 | Elephant | Dja Biosphere Reserve | Cameroon | 0 | 1 | No | IUCN SSC A.P.E.S |  |
| 45 | Elephant | Campo Ma'an National Park | Cameroon | 0 | 1 | No | IUCN SSC A.P.E.S |  |
| 46 | Chimpanzee | Mt Cameroon National Park | Cameroon | 1 | 1 | Yes | IUCN SSC A.P.E.S |  |
| 47 | Elephant | Lobéké National Park | Cameroon | 0 | 1 | No | IUCN SSC A.P.E.S |  |
| 48 | Elephant | Mt Cameroon National Park | Cameroon | 1 | 1 | Yes | IUCN SSC A.P.E.S |  |
| 49 | Chimpanzee | Odzala-Kokoua National Park | Congo Republic | 0 | 1 | No | IUCN SSC A.P.E.S |  |
| 50 | Elephant | Odzala-Kokoua National Park | Congo Republic | 0 | 1 | No | IUCN SSC A.P.E.S |  |
| 51 | Elephant | Dzanga-Sangha Complex of Protected Areas | Central African Republic | 0 | 1 | No | IUCN SSC A.P.E.S |  |
| 52 | Grizzly bear / Brown bear | Pyrénées National Park | France | 1 | 1 | Yes | LPD |  |
| 53 | Purple heron | Doñana National Park | Spain | 0 | 1 | No | LPD |  |
| 54 | Glossy ibis | Doñana National Park | Spain | 1 | 1 | Yes | LPD |  |
| 55 | Red fox | Vanoise National Park | France | 0 | 0 | Yes | <http://biodiversite.vanoise-parcnational.fr/> |  |
| 56 | Griffon vulture | Vanoise National Park | France | 1 | 1 | Yes | <http://biodiversite.vanoise-parcnational.fr/> |  |
| 57 | Red-billed chough | Vanoise National Park | France | 1 | 1 | Yes | <http://biodiversite.vanoise-parcnational.fr/> |  |
| 58 | Alpine ibex | Vanoise National Park | France | 1 | 1 | Yes | <http://biodiversite.vanoise-parcnational.fr/> |  |
| 59 | Chamois | Vanoise National Park | France | 1 | 1 | Yes | <http://biodiversite.vanoise-parcnational.fr/> |  |
| 60 | Mistle thrush | Vanoise National Park | France | 1 | 1 | Yes | <http://biodiversite.vanoise-parcnational.fr/> |  |
| 61 | European badger | Vanoise National Park | France | 0 | 0 | Yes | <http://biodiversite.vanoise-parcnational.fr/> |  |
| 62 | Wolf | Vanoise National Park | France | 1 | 1 | Yes | <http://biodiversite.vanoise-parcnational.fr/> |  |
| 63 | Cinereous vulture | Vanoise National Park | France | 1 | 1 | Yes | <http://biodiversite.vanoise-parcnational.fr/> |  |
| 64 | Sparrowhawk | Vanoise National Park | France | 1 | 0 | No | <http://biodiversite.vanoise-parcnational.fr/> |  |
| 65 | Red fox | Écrins National Park | France | 1 | 1 | Yes | <https://biodiversite.ecrins-parcnational.fr/> |  |
| 66 | Griffon vulture | Écrins National Park | France | 1 | 1 | Yes | <https://biodiversite.ecrins-parcnational.fr/> |  |
| 67 | red-billed chough | Écrins National Park | France | 1 | 1 | Yes | <https://biodiversite.ecrins-parcnational.fr/> |  |
| 68 | Alpine ibex | Écrins National Park | France | 1 | 1 | Yes | <https://biodiversite.ecrins-parcnational.fr/> |  |
| 69 | Chamois | Écrins National Park | France | 1 | 1 | Yes | <https://biodiversite.ecrins-parcnational.fr/> |  |
| 70 | Mistle thrush | Écrins National Park | France | 1 | 1 | Yes | <https://biodiversite.ecrins-parcnational.fr/> |  |
| 71 | Bearded vulture | Écrins National Park | France | 1 | 1 | Yes | <https://biodiversite.ecrins-parcnational.fr/> |  |
| 72 | Short-toed snake eagle | Écrins National Park | France | 1 | 1 | Yes | <https://biodiversite.ecrins-parcnational.fr/> |  |
| 73 | European badger | Écrins National Park | France | 1 | 1 | Yes | <https://biodiversite.ecrins-parcnational.fr/> |  |
| 74 | Wolf | Écrins National Park | France | 1 | 1 | Yes | <https://biodiversite.ecrins-parcnational.fr/> |  |
| 75 | European bison | Białowieża National Park | Poland | 1 | 1 | Yes | LPD |  |
| 76 | Forest elephant | Bui National Park | Ghana | 1 | 1 | Yes | LPD |  |
| 77 | Otter (*Lutra lutra*) | Nationalpark Unteres Odertal | Germany | 1 | 1 | Yes | PA report |  |
| 78 | Wild boar | Nationalpark Unteres Odertal | Germany | 1 | 1 | Yes | PA report |  |
| 79 | Lesser spotted eagle | Nationalpark Unteres Odertal | Germany | 1 | 1 | Yes | PA report |  |
| 80 | Eagle owl (Bubo bubo | Nationalpark Unteres Odertal | Germany | 1 | 1 | Yes | PA report |  |
| 81 | Osprey (Pandion haliaetus) or Hawk | Nationalpark Unteres Odertal | Germany | 1 | 1 | Yes | PA report |  |
| 82 | See adler | Nationalpark Unteres Odertal | Germany | 1 | 1 | Yes | PA report |  |
| 83 | Honey Buzzard (Pernis apivorus) | Nationalpark Unteres Odertal | Germany | 1 | 1 | Yes | PA report |  |
| 84 | Red Kite (Milvus milvus) | Nationalpark Unteres Odertal | Germany | 0 | 0 | Yes | PA report |  |
| 85 | Marsh harrier (Circus aeruginosus | Nationalpark Unteres Odertal | Germany | 1 | 1 | Yes | PA report |  |
| 86 | Hen harrier (Circus cyaneus | Nationalpark Unteres Odertal | Germany | 1 | 1 | Yes | PA report |  |
| 87 | European nightjar | Nationalpark Unteres Odertal | Germany | 1 | 1 | Yes | PA report |  |
| 88 | black tern | Nationalpark Unteres Odertal | Germany | 1 | 1 | Yes | PA report |  |
| 89 | Bluethroat | Nationalpark Unteres Odertal | Germany | 1 | 1 | Yes | PA report |  |
| 90 | corn crake | Nationalpark Unteres Odertal | Germany | 1 | 1 | Yes | PA report |  |
| 91 | Ravens | Nationalpark Unteres Odertal | Germany | 1 | 1 | Yes | PA report |  |
| 92 | Middle spotted woodpecker | Nationalpark Unteres Odertal | Germany | 1 | 1 | Yes | PA report |  |
| 93 | red-breasted flycatcher | Nationalpark Unteres Odertal | Germany | 1 | 1 | Yes | PA report |  |
| 94 | African elephants | Ivindo National Park | Gabon | 0 | 0 | Yes | Koerner et al., 2016 |  |
| 95 | blue duiker | Ivindo National Park | Gabon | 0 | 0 | Yes | Koerner et al., 2016 |  |
| 96 | yellow-backed duiker | Ivindo National Park | Gabon | 0 | 0 | Yes | Koerner et al., 2016 |  |
| 97 | Chimpanzee | Ivindo National Park | Gabon | 0 | 0 | Yes | Koerner et al., 2016 |  |
| 98 | Gorillas | Ivindo National Park | Gabon | 0 | 0 | Yes | Koerner et al., 2016 |  |
| 99 | Crowned Guenon | Ivindo National Park | Gabon | 1 | 1 | Yes | Koerner et al., 2016 |  |
| 100 | Moustached guenon | Ivindo National Park | Gabon | 0 | 0 | Yes | Koerner et al., 2016 |  |
| 101 | Grey-cheeked mangabey | Ivindo National Park | Gabon | 0 | 0 | Yes | Koerner et al., 2016 |  |
| 102 | Red-tailed monkey | Ivindo National Park | Gabon | 0 | 0 | Yes | Koerner et al., 2016 |  |

References

1. Coad L, Watson JEM, Geldmann J, Burgess ND, Leverington F, Hockings M, et al. Widespread shortfalls in protected area resourcing undermine efforts to conserve biodiversity. Front Ecol Environ. 2019;17: 259–264. doi:10.1002/fee.2042

2. Schulze K, Knights K, Coad L, Geldmann J, Leverington F, Eassom A, et al. An assessment of threats to terrestrial protected areas. Conserv Lett. 2018; 1–10. doi:10.1111/conl.12435

3. Barnes MD, Craigie ID, Harrison LB, Geldmann J, Collen B, Whitmee S, et al. Wildlife population trends in protected areas predicted by national socio-economic metrics and body size. Nat Commun. 2016;7: 1–9. doi:10.1038/ncomms12747

4. Andermann T, Faurby S, Turvey ST, Antonelli A, Silvestro D. The past and future human impact on mammalian diversity. Sci Adv. 2020;6: eabb2313. doi:10.1126/sciadv.abb2313

5. Wolf C, Ripple WJ. Range contractions of the world’s large carnivores. R Soc Open Sci. 2017;4. doi:10.1098/rsos.170052

6. de Boer WF, van Langevelde F, Prins HHT, de Ruiter PC, Blanc J, Vis MJP, et al. Understanding spatial differences in African elephant densities and occurrence, a continent-wide analysis. Biol Conserv. 2013;159: 468–476. doi:10.1016/j.biocon.2012.10.015

7. Keane A, Brooke MDL, McGowan PJK. Correlates of extinction risk and hunting pressure in gamebirds (Galliformes). Biol Conserv. 2005;126: 216–233. doi:10.1016/j.biocon.2005.05.011

8. Lamb CT, Ford AT, McLellan BN, Proctor MF, Mowat G, Ciarniello L, et al. The ecology of human–carnivore coexistence. Proc Natl Acad Sci U S A. 2020;117: 17876–17883. doi:10.1073/pnas.1922097117

9. Brooks JS, Waylen K a, Borgerhoff Mulder M. How national context, project design, and local community characteristics influence success in community-based conservation projects. Proc Natl Acad Sci U S A. 2012;109: 21265–70. doi:10.1073/pnas.1207141110

10. Brooks J, Waylen K, Mulder M, Access O. Assessing community-based conservation projects: A systematic review and multilevel analysis of attitudinal, behavioral, ecological, and economic outcomes. Environ Evid. 2013;2: 2. doi:10.1186/2047-2382-2-2

11. Waylen K a, Fischer A, McGowan PJK, Thirgood SJ, Milner-Gulland EJ. Effect of local cultural context on the success of community-based conservation interventions. Conserv Biol. 2010;24: 1119–29. doi:10.1111/j.1523-1739.2010.01446.x

12. Ostrom E. Governing the Commons: the evolutions of institutions for collective action. Political economy of institutions and decisions. 1990. Available: http://www.mendeley.com/catalog/governing-commons-evolutions-institutions-collective-action/

13. Wilson DS, Ostrom E, Cox ME. Generalizing the core design principles for the efficacy of groups. J Econ Behav Organ. 2013;90. doi:10.1016/j.jebo.2012.12.010

14. Young JC, Jordan A, R. Searle K, Butler A, S. Chapman D, Simmons P, et al. Does stakeholder involvement really benefit biodiversity conservation? Biol Conserv. 2013;158: 359–370. doi:10.1016/j.biocon.2012.08.018

15. Young JC, Searle K, Butler A, Simmons P, Watt AD, Jordan A. The role of trust in the resolution of conservation conflicts. Biol Conserv. 2016;195: 196–202. doi:10.1016/j.biocon.2015.12.030

16. Kablan YA, Diarrassouba A, Mundry R, Campbell G, Normand E, Kühl HS, et al. Effects of anti-poaching patrols on the distribution of large mammals in Taï National Park, Côte d’Ivoire. Oryx. 2017; 1–10. doi:10.1017/S0030605317001272

17. Laurance WF, Carolina Useche D, Rendeiro J, Kalka M, Bradshaw CJA, Sloan SP, et al. Averting biodiversity collapse in tropical forest protected areas. Nature. 2012;489: 290–293. doi:10.1038/nature11318

18. N’Goran PK, Boesch C, Mundry R, N’Goran EK, Herbinger I, Yapi FA, et al. Hunting, law enforcement, and African primate conservation. Conserv Biol. 2012;26: 565–71. doi:10.1111/j.1523-1739.2012.01821.x

19. Nielsen MR, Meilby H, Smith-Hall C, Pouliot M, Treue T. The Importance of Wild Meat in the Global South. Ecol Econ. 2018;146: 696–705. doi:10.1016/j.ecolecon.2017.12.018

20. van Beeck Calkoen STSSTS, Mühlbauer L, Andrén H, Apollonio M, Balčiauskas L, Belotti E, et al. Ungulate management in European national parks: Why a more integrated European policy is needed. J Environ Manage. 2020;260. doi:10.1016/j.jenvman.2020.110068

21. Jordan NR, Smith BP, Appleby RG, van Eeden LM, Webster HS. Addressing inequality and intolerance in human–wildlife coexistence. Conserv Biol. 2020;00: 1–8. doi:10.1111/cobi.13471

22. Lozano J, Olszańska A, Morales-Reyes Z, Castro AA, Malo AF, Moleón M, et al. Human-carnivore relations: A systematic review. Biol Conserv. 2019;237: 480–492. doi:10.1016/j.biocon.2019.07.002

23. Geldmann J, Coad L, Barnes MD, Craigie ID, Woodley S, Balmford A, et al. A global analysis of management capacity and ecological outcomes in terrestrial protected areas. Conserv Lett. 2018;11: 1–10. doi:10.1111/conl.12434

24. Benítez-López A, Alkemade R, Schipper AM, Ingram DJ, Verweij PA, Eikelboom JAJ, et al. The impact of hunting on tropical mammal and bird populations. Science (80- ). 2017;356: 180–183. doi:10.1126/science.aaj1891

25. Zoological Society of London, WWF. Living Planet Database. 2014. Available: https://livingplanetindex.org/home/index

26. Kühl HS, Williamson L, Sanz C, Morgan D, Boesch C. A.P.E.S., IUCN SSC A.P.E.S. database. 2007. Available: http://apesportal.eva.mpg.de

27. PAME. Meaningful Engagement of Indigenous Peoples and Local Communities in Marine Activities. 2021. Available: https://oaarchive.arctic-council.org/handle/11374/2650

28. Gavin MC, Solomon JN, Blank SG. Measuring and Monitoring Illegal Use of Natural Resources. Conserv Biol. 2009;24: 89–100. doi:10.1111/j.1523-1739.2009.01387.x

29. Golden CD, Wrangham RW, Brashares JS. Assessing the accuracy of interviewed recall for rare , highly seasonal events : the case of wildlife consumption in Madagascar. 2011; 597–603. doi:10.1111/acv.12047

30. Jones JPG, Andriamarovololona MM, Hockley N, Gibbons JM, Milner-Gulland EJ. Testing the use of interviews as a tool for monitoring trends in the harvesting of wild species. J Appl Ecol. 2008;45: 1205–1212. doi:10.1111/j.1365-2664.2008.01487.x
