# Supplementary material for: Analysis of differences and commonalities in wildlife hunting across the Africa-Europe South-North gradient
Source: PLoS Biol. 2022 Aug 30;20(8):e3001707. doi: 10.1371/journal.pbio.3001707 (PMC9426919; doi:10.1371/journal.pbio.3001707)
Supplement: S2 Appendix — (PDF) [file pbio.3001707.s002.pdf]

German Centre for Integrative Biodiversity Research (iDiv)
Halle-Jena-Leipzig

Questionnaire for Protected area managers

Evaluating the Socio-economic Cost and Effectiveness of Conservation Interventions in European and African Protected Areas

General instructions

Thank you very much for your time and willingness to participate in this study. We are researchers from German Center for Integrative Biodiversity Research (iDiv). We are conducting a survey on conservation interventions in different African and European national parks to find out which interventions are more effective for biodiversity conservation. Participation in this survey is purely voluntary. The information given in the questionnaire will be used solely for scientific research; which is the only motive of this survey. The responses will be kept absolutely confidential. No associations will be made between your responses and your personality. Thank you once again for your time and willingness to participate in this study. The survey will take around 3 hours.

Please note that in different parts of the questionnaire, we may ask you about your confidence level in your responses. The aim of this question is to differentiate between "high confidence" responses and "speculative responses". It is not about you knowledge or ability. Thank you once again for your time!

I. Personal profile

1. What is your education level? ______________ Field of expertise? ________________
2. Gender: Male _____ Female _____ Other _____
3. Nationality ___________________
4. What is your position in the park management? __________________________
5. For how long have you been working in this position? _________________

II. General profile

6. Name of the national park (NP) ___________________________________ Country _________________
7. How much do you rely on the following sources of evidence for the park management decision-making?

Source	Degree of reliance<br>**1= very high**<br>**2= high**<br>**3= medium**<br>**4= low**<br>**5= very low**<br>**6= never**
Own experience	
Experience shared from other national parks in the country	
Experience shared from other countries	
Published articles	
Published or unpublished reports	
Expert advice from the national park (e.g. internal scientists)	
Expert advice from external scientists	
Traditional knowledge	
Others, specify	

8. Was there any change to the size of the NP since its creation?
   1= Yes, increased, 2= Yes, decreased, 3= No change
9. What was the annual budget of the NP for 2017? ________________________(**mention the currency)**
10. How do you rate the **sufficiency** of the 2017 budget for achieving the parks objectives?
    1= very high, 2= high, 3= medium, 4= low, 5= very low.
11. How do you rate the performance of your NP in achieving its objectives for the year 2017?
    1= very high, 2= high, 3= medium, 4= low, 5= very low.
12. What are the sources of the budget for 2017? Please fill in the following table.

Source	Share of the total budget (in %)
Government	
International organizations	
Local NGOs	
Private organization	
NP revenue	
Others (please specify):	

13. Is the 2017 budget representative of the budget over the last five years?
    Select: **1= yes, 2= No, it was lower, 3= No, it was higher**
14. If the answer for #13 is 'No', how much was the change in percentage? ______
    What was the reason for the change?
    ____
    ____
15. Is your NP a member of any international network of NPs?
    Select: **1= Yes, 2= No.**
16. If your answer for question #15 is 'Yes', please give the name of the network of the NPs
    ____

III. Conservation interventions

Which of the following conservation programs/interventions have been implemented in your national park in the past 10 years?
Please fill in the tables for conservations implemented in your NP.

a. Presence of multipurpose buffer zone
Select, **1= Implemented or, 2= Not implemented.** *If '2= Not implemented', go to the next question.*

When did it start? (year)	When did it end? (leave blank if ongoing)	The size of the buffer zone (in hectares, or specify the unit)	Average **annual budget** (specify the currency)	Funding agency (e.g. local NGO, international NGO, Gov't, private, others)	The main objective of the intervention	How do you rate the effectiveness of this intervention in achieving its objective? **1= very high 2= high 3= medium 4= low 5= very low**

b. Conduct regular patrols for illegal activities (poaching, logging, agriculture, grazing, etc.)
Select, **1= Implemented or, 2= Not implemented.** If '2= Not implemented', go to the next question.

When did it start? (year)	When did it end? (leave blank if ongoing)	How many rangers have you had *per year* during this intervention? (on average)	How many illegal activities were reported **per year** (on average)?	Do the rangers use arms? 1= yes 2= no	Average **annual budget** (specify currency)	Funding agency (e.g. local NGO, international NGO, Gov't, private, others)	The main objective of the intervention	How do you rate the effectiveness of this intervention in achieving its objective? **1= very high 2= high 3= medium 4= low 5= very low**

c. Removing traps and snares
Select, **1= Implemented or, 2= Not implemented.** If '2= Not implemented', go to the next question.

When did it start? (year)	When did it end? (leave blank if ongoing)	How many snares/traps are removed **per year**? (on average)	Average **annual budget** (specify the currency)	Funding agency (e.g. local NGO, international NGO, Gov't, private, others)	The main objective of the intervention	How do you rate the effectiveness of this intervention in achieving its objective? **1= very high 2= high 3= medium 4=low 5= very low**

d. Inspect markets and/or cars on the road for illegal bushmeat
Select, **1= Implemented or, 2= Not implemented.** If '2= Not implemented', go to the next question.

When did it start? (year)	When did it end? (leave blank if ongoing)	How many snares/traps are removed ***per year***? (on average)	Average **annual budget** (specify the currency)	Funding agency (e.g. local NGO, international NGO, Gov't, private, others)	The main objective of the intervention	How do you rate the effectiveness of this intervention in achieving its objective? **1= very high 2= high 3= medium 4= low 5= very low**

e. Planting of buffer crops such as tea, coffee, etc. at the edge of the national park
Select, **1= Implemented or, 2= Not implemented.** If '2= Not implemented', go to the next question.

When did it start? (year)	When did it end? (leave blank if ongoing)	The size of the land covered with these crops? (in hectares, or specify the unit)	Average **annual budget** (specify the currency)	Funding agency (e.g. local NGO, international NGO, Gov't, private, others)	The main objective of the intervention	How do you rate the effectiveness of this intervention in achieving its objective? **1= very high 2= high 3= medium 4= low 5= very low**

f. Permanent presence of research site/institution
Select, **1= Implemented or, 2= Not implemented.** If '2= Not implemented', go to the next question.

When did it start? (year)	When did it end? (leave blank if ongoing)	How many employees has the institution had per year? (on average)	What is the size of the research zone? (in hectares or specify the unit)	Average **annual budget** (specify the currency)	Funding agency (local NGO, international NGO, Gov't, private, others)	The main objective of the intervention	How do you rate the effectiveness of this intervention in achieving its objective? **1= very high 2= high 3= medium 4= low 5= very low**

g. Species management
(e.g. protection/translocation of species, protection/translocation of nests for birds, creating/modifying ponds for amphibians and/or reptiles, clear vegetation for amphibians, supplementary feeding etc.)
Select, **1= Implemented or, 2= Not implemented.** If '2= Not implemented', go to the next question.

Type of species management	Name of the species	When did it start? (year)	When did it end? (leave blank if ongoing)	Intensity of the implementation (please fill this column as specified below for each intervention)	Average **annual budget** (specify currency)	Funding agency (e.g. local NGO, international NGO, Gov't, private, others)	The main objective of the intervention	How do you rate the effectiveness of this intervention in achieving its objective?<br>**1= very high**<br>**2= high**<br>**3= medium**<br>**4= low**<br>**5= very low**
protection/ translocation of species				Number of individuals protected/ translocated				
protection/ translocation of nests				Number of nests protected/ translocated				
supplementary feeding				Number of individuals fed				
creating/ modifying ponds for amphibians and/or reptiles				Number of ponds created/ modified				
clearing vegetation for amphibians and/or reptiles				Size of vegetation cleared (in hectares)				
others, please specify								

h. Species management (e.g. population control through hunting or other mechanisms)
Select, **1= Implemented or, 2= Not implemented.** If '2= Not implemented', go to the next question.

Type of species management (specify the species)	When did it start? (year)	When did it end? (leave blank if ongoing)	How many individuals **total** have been killed?	Average **annual budget** (specify the currency)	Funding agency (e.g. local NGO, international NGO, Gov't, private, others)	The main objective of the intervention	How do you rate the effectiveness of this intervention in achieving its objective?<br>**1= very high**<br>**2= high**<br>**3= medium**<br>**4= low**<br>**5= very low**

i. Ecological restoration: Reintroduction of any indigenous species
Select, **1= Implemented or, 2= Not implemented.** If '2= Not implemented', go to the next question.

Species re-introduced	When did it start? (year)	When did it end? (leave blank if ongoing)	Number of **total** individuals re-introduced	Average **annual budget** (specify the currency)	Funding agency (e.g. local NGO, international NGO, Gov't, private, others)	The main objective of the intervention	How do you rate the effectiveness of this intervention in achieving its objective?<br>**1= very high**<br>**2= high**<br>**3= medium**<br>**4= low**<br>**5= very low**

j. Ecological restoration: Re-vegetation
Select, **1= Implemented or, 2= Not implemented.** If '2= Not implemented', go to the next question.

When did it start? (year)	When did it end? (leave blank if ongoing)	The size of area covered by re-vegetation (in hectares or specify the unit)	Average **annual budget** (specify the currency)	Funding agency (e.g. local NGO, international NGO, Gov't, private, others)	The main objective of the intervention	How do you rate the effectiveness of this intervention in achieving its objective?<br>**1= very high**<br>**2= high**<br>**3= medium**<br>**4= low**<br>**5= very low**

k. Ecological restoration: Re-habilitation
Select, **1= Implemented or, 2= Not implemented.** If '2= Not implemented', go to the next question.

When did it start? (year)	When did it end? (leave blank if ongoing)	The size of area covered by re-vegetation (in hectares or specify the unit)	Average **annual budget** (specify the currency)	Funding agency (e.g. local NGO, international NGO, Gov't, private, others)	The main objective of the intervention	How do you rate the effectiveness of this intervention in achieving its objective? **1= very high 2= high 3= medium 4= low 5= very low**

l. Payment for local communities/farmers (e.g. cover the cost of conservation measures, compensation for damage by wildlife etc.)
Select, **1= Implemented or, 2= Not implemented.** If '2= Not implemented', go to the next question.

When did it start? (year)	When did it end? (leave blank if ongoing)	Number of beneficiaries per year (on average)	Payment per beneficiary per **year** (specify currency)	Average **annual budget** (specify the currency)	Funding agency (e.g. local NGO, international NGO, Gov't, private, others)	The main objective of the intervention	How do you rate the effectiveness of this intervention in achieving its objective? **1= very high 2= high 3= medium 4= low 5= very low**

m. Provision of community projects such as schools, health facilities, water wells, village roads etc.
Select, **1= Implemented or, 2= Not implemented.** If '2= Not implemented', go to the next question.

Project	When did it start? (year)	When did it end? (leave blank if ongoing)	Number of projects implemented	Average **annual budget** (specify the currency)	Funding agency (e.g. local NGO, international NGO, Gov't, private, others)	The main objective of the intervention	How do you rate the effectiveness of this intervention in achieving its objective? **1= very high 2= high 3= medium 4= low 5= very low**
schools							
health facilities							
roads (in km)							

n. Sharing tourism revenue to the local community (e.g. sharing revenue from entry fees paid to NPs by tourists, etc.)
Select, **1= Implemented or, 2= Not implemented.** If '2= Not implemented', go to the next question.

When did it start? (year)	When did it end? (leave blank if ongoing)	The amount of money shared to local community per year (on average)	The main objective of the intervention	How do you rate the effectiveness of this intervention in achieving its objective? **1=very high 2= high 3= medium 4= Low 5= very low**

o. Sustainable tourism/ecotourism management (STM)
Select, **1= Implemented or, 2= Not implemented.** If '2= Not implemented', go to the next question.

When did it start? (year)	When did it end? (leave blank if ongoing)	Do you have an independent office and/or plan for STM?	Average **annual budget** (specify the currency)	Funding agency (e.g. local NGO, international NGO, Gov't, private, others)	The main objective of the intervention	How do you rate the effectiveness of this intervention in achieving its objective? **1= very high 2= high 3= medium 4= low 5= very low**

p. Alternative livelihood projects (e.g. farming cane rats, poultry, farm pond fishing, vegetable gardening, craft making, beekeeping, fuel-efficient stoves, microcredit access etc.)
Select, **1= Implemented or, 2= Not implemented.** If '2= Not implemented', go to the next question.

When did it start? (year)	When did it end? (leave blank if ongoing)	The number of beneficiaries per year (on average)	Average **annual budget** (specify the currency)	Funding agency (e.g. local NGO, international NGO, Gov't, private, others)	The main objective of the intervention	How do you rate the effectiveness of this intervention in achieving its objective? **1= very high 2= high 3= medium 4= low 5= very low**

q. Environmental/conservation education and awareness creation

Select, **1= Implemented or, 2= Not implemented.** If '2= Not implemented', go to the next question.

When did it start? (year)	When did it end? (leave blank if ongoing)	Number of staff per year actively working in the project (on average)	Number of awareness campaigns *per year*	Average **annual budget** (specify the currency)	Funding agency (e.g. local NGO, international NGO, Gov't, private, others)	The main objective of the intervention	How do you rate the effectiveness of this intervention in achieving its objective? **1= very high 2= high 3= medium 4= low 5= very low**

r. Community based conservation (CBC) (e.g. co-management or joint management of a protected area or buffer zone with the local community)

Select, **1= Implemented or, 2= Not implemented.** If '2= Not implemented', go to the next question.

When did it start? (year)	When did it end? (leave blank if ongoing)	The size of the area under CBC (in hectares)	Average **annual budget** (specify the currency)	Funding agency (e.g. local NGO, international NGO, Gov't, private, others)	The main objective of the intervention	How do you rate the effectiveness of this intervention in achieving its objective? **1= very high 2= high 3= medium 4= low 5= very low**

s. Please specify if there are other interventions implemented in the park in the past 10 years

Name of intervention	When did it start? (year)	When did it end? (leave blank if ongoing)	Average **annual budget** (specify the currency)	Funding agency (e.g. local NGO, international NGO, Gov't, private, others)	The main objective of the intervention	How do you rate the effectiveness of this intervention in achieving its objective? **1= very high 2= high 3= medium 4= low 5= very low**

IV. Biodiversity related questions

Please fill in the following tables according to the instructions to the specific questions. Please rate the **relative changes in the past 10 years** in the abundance and/or species richness of fauna in your national park by writing **-3 if there was a decline of greater than 30%, -2= a decline of 10-30%, -1= decline of <10%, 0= No change, +1= an increase of <10%, +2= an increase of 10-30%, +3= an increase of >30%.** Please feel free to skip questions for which you have little or no knowledge. Please note that in different parts of this section of the questionnaire, there are some overlapping categories, which are intentionally included for the benefit of the project.

Mammals' abundance and species richness

1. Top mammalian predators (e.g. a=bears, b=cheetah, c=hyena, d=leopard, e=lion, f=lynx, g=wolf).

Note: As the list here is not exhaustive, please write the name of the species if it is not given in the list.

		1	2	3	4	5
**Species abundance**		Name<br>(write only the letter)	Change<br>**-3= decline of >30%<br>-2= decline of 10-30%<br>-1= decline of <10%<br>0= no change<br>+1= increase of <10%<br>+2= increase of 10-30%<br>+3= increase of >30%**	Current amount<br>Specify unit (e.g. number of individuals, encounter rate/km^2 or number of groups/km^2)	Year for the amount specified in **column 3**	Rate your confidence level for the accuracy of your response<br>**1= very high<br>2= high<br>3= medium<br>4= low<br>5= very low**
	Species 1					
	Species 2					
	Species 3					
	Species 4					
	Species 5					
	Species 6					
**Species richness**				N/A	N/A	

2. Small predatory mammals (e.g. a=fox, b=jackal, c=Marten, d=wild dog, e=wild cat etc.)

Note: As the list here is not exhaustive, please write the name of the species if it is not given in the list.

		1	2	3	4	5
**Species abundance**		Name<br>(write only the letter)	Change<br>**-3= decline of >30%<br>-2= decline of 10-30%<br>-1= decline of <10%<br>0= no change<br>+1= increase of <10%<br>+2= increase of 10-30%<br>+3= increase of >30%**	Current amount<br>Specify unit (e.g. number of individuals, encounter rate/km2 or number of groups/km2)	Year for the amount specified in **column 3**	Rate your confidence level for the accuracy of your response<br>**1= very high<br>2= high<br>3= medium<br>4= low<br>5= very low**
	Species 1					
	Species 2					
	Species 3					
	Species 4					
	Species 5					
	Species 6					
**Species richness**				N/A	N/A	

3. Large non-predatory (or herbivorous) mammals
(e.g. a=elephants, b=rhinos, c=hippos, d=giraffes, e=Oryx, f=Kudu, g=Eland, h=wildebeest, i=hartebeest, j=waterbuck, k=Topi / Tsessebe antelope, l=Zebra, m=wild ass, n=wild horse, o=Red deer, p=Moose)
Note: As the list here is not exhaustive, please write the name of the species if it is not given in the list.

		1	2	3	4	5
**Species abundance**		Name (write only the letter)	Change<br>**-3= decline of >30%**<br>**-2= decline of 10-30%**<br>**-1= decline of <10%**<br>**0= no change**<br>**+1= increase of <10%**<br>**+2= increase of 10-30%**<br>**+3= increase of >30%**	Current amountSpecify unit (e.g. number of individuals, encounter rate/km2 or number of groups/km2)	Year for the amount specified in **column 3**	Rate your confidence level for the accuracy of your response<br>**1= very high**<br>**2= high**<br>**3= medium**<br>**4= low**<br>**5= very low**
	Species 1					
	Species 2					
	Species 3					
	Species 4					
	Species 5					
	Species 6					
	Species 7					
	Species 8					
**Species richness**				N/A	N/A	

4. Small to medium sized non-predatory (or herbivorous) mammals
(e.g. a=duiker, b=roe deer, c=bushbuck, d=impala)
Note: As the list here is not exhaustive, please write the name of the species if it is not given in the list.

		1	2	3	4	5
**Species abundance**		Name<br>(write only the letter)	Change<br>**-3= decline of >30%**<br>**-2= decline of 10-30%**<br>**-1= decline of <10%**<br>**0= no change**<br>**+1= increase of <10%**<br>**+2= increase of 10-30%**<br>**+3= increase of >30%**	Current amount<br>Specify unit (e.g. number of individuals, encounter rate/km2 or number of groups/km2)	Year for the amount specified in **column 3**	Rate your confidence level for the accuracy of your response<br>**1= very high**<br>**2= high**<br>**3= medium**<br>**4= low**<br>**5= very low**
	Species 1					
	Species 2					
	Species 3					
	Species 4					
	Species 5					
	Species 6					
**Species richness**				N/A	N/A	

5. Insectivorous mammals (e.g. a=hedgehog, b=shrew, c= mole, etc.)

Note: As the list here is not exhaustive, please write the name of the species if it is not given in the list.

		1	2	3	4	5
**Species abundance**		Name<br>(write only the letter)	Change<br>**-3= decline of >30%**<br>**-2= decline of 10-30%**<br>**-1= decline of <10%**<br>**0= no change**<br>**+1= increase of <10%**<br>**+2= increase of 10-30%**<br>**+3= increase of >30%**	Current amount<br>Specify unit (e.g. number of individuals, encounter rate/km2 or number of groups/km2)	Year for the amount specified in **column 3**	Rate your confidence level for the accuracy of your response<br>**1= very high**<br>**2= high**<br>**3= medium**<br>**4= low**<br>**5= very low**
	Species 1					
	Species 2					
	Species 3					
	Species 4					
	Species 5					
**Species richness**				N/A	N/A	

6. Omnivorous/opportunistic mammals (e.g. pigs, all> 1 kg)

Note: As the list here is not exhaustive, please write the name of the species if it is not given in the list.

		1	2	3	4	5
**Species abundance**		Name<br>(write only the letter)	Change<br>**-3= decline of >30%**<br>**-2= decline of 10-30%**<br>**-1= decline of <10%**<br>**0= no change**<br>**+1= increase of <10%**<br>**+2= increase of 10-30%**<br>**+3= increase of >30%**	Current amount<br>Specify unit (e.g. number of individuals, encounter rate/km2 or number of groups/km2)	Year for the amount specified in **column 3**	Rate your confidence level for the accuracy of your response<br>**1= very high**<br>**2= high**<br>**3= medium**<br>**4= low**<br>**5= very low**
	Species 1					
	Species 2					
	Species 3					
	Species 4					
**Species richness**				N/A	N/A	

7. Great apes (e.g. a=Chimpanzees, b=Gorillas, c=bonobos)

Note: As the list here is not exhaustive, please write the name of the species if it is not given in the list.

		1	2	3	4	5
**Species abundance**		Name<br>(write only the letter)	Change<br>**-3= decline of >30%<br>-2= decline of 10-30%<br>-1= decline of <10%<br>0= no change<br>+1= increase of <10%<br>+2= increase of 10-30%<br>+3= increase of >30%**	Current amount<br>Specify unit (e.g. number of individuals, encounter rate/km2 or number of groups/km2)	Year for the amount specified in **column 3**	Rate your confidence level for the accuracy of your response<br>**1= very high<br>2= high<br>3= medium<br>4= low<br>5= very low**
	Species 1					
	Species 2					
	Species 3					
	Species 4					
**Species richness**				N/A	N/A	

8. Small bodied primates (e.g. a=baboons, b=Blue monkey, c=red-tailed monkey, d=Guereza)

Note: As the list here is not exhaustive, please write the name of the species if it is not given in the list.

		1	2	3	4	5
**Species abundance**		Name<br>(write only the letter)	Change<br>**-3= decline of >30%<br>-2= decline of 10-30%<br>-1= decline of <10%<br>0= no change<br>+1= increase of <10%<br>+2= increase of 10-30%<br>+3= increase of >30%**	Current amount<br>Specify unit (e.g. number of individuals, encounter rate/km2 or number of groups/km2)	Year for the amount specified in **column 3**	Rate your confidence level for the accuracy of your response<br>**1= very high<br>2= high<br>3= medium<br>4= low<br>5= very low**
	Species 1					
	Species 2					
	Species 3					
	Species 4					
	Species 5					
**Species richness**				N/A	N/A	

9. Invasive mammal species (not native to the NP, e.g. raccoon)

Note: As the list here is not exhaustive, please write the name of the species if it is not given in the list.

		1	2	3	4	5
**Species abundance**		Name<br>(write only the letter)	Change<br>**-3= decline of >30%**<br>**-2= decline of 10-30%**<br>**-1= decline of <10%**<br>**0= no change**<br>**+1= increase of <10%**<br>**+2= increase of 10-30%**<br>**+3= increase of >30%**	Current amount<br>Specify unit (e.g. number of individuals, encounter rate/km2 or number of groups/km2)	Year for the amount specified in **column 3**	Rate your confidence level for the accuracy of your response<br>**1= very high**<br>**2= high**<br>**3= medium**<br>**4= low**<br>**5= very low**
	Species 1					
	Species 2					
	Species 3					
**Species richness**				N/A	N/A	

10. Rodents (<1 kg) (e.g. a=jerboa, b=field mouse, c=squirrel, d=hamster etc.)

Note: As the list here is not exhaustive, please write the name of the species if it is not given in the list.

		1	2	3	4	5
**Species abundance**		Name<br>(write only the letter)	Change<br>**-3= decline of >30%**<br>**-2= decline of 10-30%**<br>**-1= decline of <10%**<br>**0= no change**<br>**+1= increase of <10%**<br>**+2= increase of 10-30%**<br>**+3= increase of >30%**	Current amount<br>Specify unit (e.g. number of individuals, encounter rate/km2 or number of groups/km2)	Year for the amount specified in **column 3**	Rate your confidence level for the accuracy of your response<br>**1= very high**<br>**2= high**<br>**3= medium**<br>**4= low**<br>**5= very low**
	Species 1					
	Species 2					
	Species 3					
	Species 4					
	Species 5					
	Species 6					
**Species richness**				N/A	N/A	

11. Bats (e.g. a=Fruit bat/flying fox, b= Egyptian Fruit Bat, etc.)

Note: As the list here is not exhaustive, please write the name of the species if it is not given in the list.

		1	2	3	4	5
**Species abundance**		Name<br>(write only the letter)	Change<br>**-3= decline of >30%**<br>**-2= decline of 10-30%**<br>**-1= decline of <10%**<br>**0= no change**<br>**+1= increase of <10%**<br>**+2= increase of 10-30%**<br>**+3= increase of >30%**	Current amount<br>Specify unit (e.g. number of individuals, encounter rate/km2 or number of groups/km2)	Year for the amount specified in **column 3**	Rate your confidence level for the accuracy of your response<br>**1= very high**<br>**2= high**<br>**3= medium**<br>**4= low**<br>**5= very low**
	Species 1					
	Species 2					
	Species 3					
	Species 4					
**Species richness**				N/A	N/A	

Birds' abundance and species richness

12. Raptors (e.g. a=eagles, b=falcons, c=hawks, d=owls, e= vultures)

Note: As the list here is not exhaustive, please write the name of the species if it is not given in the list.

		1	2	3	4	5
**Species abundance**		Name<br>(write only the letter)	Change<br>**-3= decline of >30%**<br>**-2= decline of 10-30%**<br>**-1= decline of <10%**<br>**0= no change**<br>**+1= increase of <10%**<br>**+2= increase of 10-30%**<br>**+3= increase of >30%**	Current amount<br>Specify unit (e.g. number of individuals, encounter rate/km2 or number of groups/km2)	Year for the amount specified in **column 3**	Rate your confidence level for the accuracy of your response<br>**1= very high**<br>**2= high**<br>**3= medium**<br>**4= low**<br>**5= very low**
	Species 1					
	Species 2					
	Species 3					
	Species 4					
	Species 5					
**Species richness**				N/A	N/A	

13. Large frugivorous birds (e.g. hornbills etc.)

Note: As the list here is not exhaustive, please write the name of the species if it is not given in the list.

		1	2	3	4	5
**Species abundance**		Name<br>(write only the letter)	Change<br>**-3= decline of >30%**<br>**-2= decline of 10-30%**<br>**-1= decline of <10%**<br>**0= no change**<br>**+1= increase of <10%**<br>**+2= increase of 10-30%**<br>**+3= increase of >30%**	Current amount<br>Specify unit (e.g. number of individuals, encounter rate/km2 or number of groups/km2)	Year for the amount specified in **column 3**	Rate your confidence level for the accuracy of your response<br>**1= very high**<br>**2= high**<br>**3= medium**<br>**4= low**<br>**5= very low**
	Species 1					
	Species 2					
**Species richness**				N/A	N/A	

14. Insect eating birds (e.g. a=Warblers etc.)

Note: As the list here is not exhaustive, please write the name of the species if it is not given in the list.

		1	2	3	4	5
**Species abundance**		Name<br>(write only the letter)	Change<br>**-3= decline of >30%**<br>**-2= decline of 10-30%**<br>**-1= decline of <10%**<br>**0= no change**<br>**+1= increase of <10%**<br>**+2= increase of 10-30%**<br>**+3= increase of >30%**	Current amount<br>Specify unit (e.g. number of individuals, encounter rate/km2 or number of groups/km2)	Year for the amount specified in **column 3**	Rate your confidence level for the accuracy of your response<br>**1= very high**<br>**2= high**<br>**3= medium**<br>**4= low**<br>**5= very low**
	Species 1					
	Species 2					
**Species richness**				N/A	N/A	

15. Seed eating birds (e.g. a=sparrows, b=finches)

Note: As the list here is not exhaustive, please write the name of the species if it is not given in the list.

		1	2	3	4	5
**Species abundance**		Name<br>(write only the letter)	Change<br>**-3= decline of >30%**<br>**-2= decline of 10-30%**<br>**-1= decline of <10%**<br>**0= no change**<br>**+1= increase of <10%**<br>**+2= increase of 10-30%**<br>**+3= increase of >30%**	Current amount<br>Specify unit (e.g. number of individuals, encounter rate/km2 or number of groups/km2)	Year for the amount specified in **column 3**	Rate your confidence level for the accuracy of your response<br>**1= very high**<br>**2= high**<br>**3= medium**<br>**4= low**<br>**5= very low**
	Species 1					
	Species 2					
**Species richness**				N/A	N/A	

16. Nectivorous birds (e.g. sunbirds, etc.)

Note: As the list here is not exhaustive, please write the name of the species if it is not given in the list.

		1	2	3	4	5
**Species abundance**		Name<br>(write only the letter)	Change<br>**-3= decline of >30%<br>-2= decline of 10-30%<br>-1= decline of <10%<br>0= no change<br>+1= increase of <10%<br>+2= increase of 10-30%<br>+3= increase of >30%**	Current amount<br>Specify unit (e.g. number of individuals, encounter rate/km2 or number of groups/km2)	Year for the amount specified in **column 3**	Rate your confidence level for the accuracy of your response<br>**1= very high<br>2= high<br>3= medium<br>4= low<br>5= very low**
	Species 1					
	Species 2					
	Species 3					
	Species 4					
**Species richness**				N/A	N/A	

17. Omnivorous birds (e.g. a=crows, b=ravens, c=rooks etc.)

Note: As the list here is not exhaustive, please write the name of the species if it is not given in the list.

		1	2	3	4	5
**Species abundance**		Name<br>(write only the letter)	Change<br>**-3= decline of >30%<br>-2= decline of 10-30%<br>-1= decline of <10%<br>0= no change<br>+1= increase of <10%<br>+2= increase of 10-30%<br>+3= increase of >30%**	Current amount<br>Specify unit (e.g. number of individuals, encounter rate/km2 or number of groups/km2)	Year for the amount specified in **column 3**	Rate your confidence level for the accuracy of your response<br>**1= very high<br>2= high<br>3= medium<br>4= low<br>5= very low**
	Species 1					
	Species 2					
	Species 3					
	Species 4					
**Species richness**				N/A	N/A	

18. Species dependent on tree cavities (e.g. a=parrots, b=woodpecker, etc.)
Note: As the list here is not exhaustive, please write the name of the species if it is not given in the list.

		1	2	3	4	5
**Species abundance**		Name<br>(write only the letter)	Change<br>**-3= decline of >30%<br>-2= decline of 10-30%<br>-1= decline of <10%<br>0= no change<br>+1= increase of <10%<br>+2= increase of 10-30%<br>+3= increase of >30%**	Current amount<br>Specify unit (e.g. number of individuals, encounter rate/km2 or number of groups/km2)	Year for the amount specified in **column 3**	Rate your confidence level for the accuracy of your response<br>**1= very high<br>2= high<br>3= medium<br>4= low<br>5= very low**
	Species 1					
	Species 2					
	Species 3					
	Species 4					
**Species richness**				N/A	N/A	

19. Non-migratory birds

		1	2	3	4	5
**Species abundance**		Name<br>(write only the letter)	Change<br>**-3= decline of >30%<br>-2= decline of 10-30%<br>-1= decline of <10%<br>0= no change<br>+1= increase of <10%<br>+2= increase of 10-30%<br>+3= increase of >30%**	Current amount<br>Specify unit (e.g. number of individuals, encounter rate/km2 or number of groups/km2)	Year for the amount specified in **column 3**	Rate your confidence level for the accuracy of your response<br>**1= very high<br>2= high<br>3= medium<br>4= low<br>5= very low**
	Species 1					
	Species 2					
	Species 3					
	Species 4					
**Species richness**				N/A	N/A	

20. Ground dwelling birds (e.g. a=hoopoe bird, b=pheasant, c=capercaillie, etc.)
Note: As the list here is not exhaustive, please write the name of the species if it is not given in the list.

		1	2	3	4	5
**Species abundance**		Name<br>(write only the letter)	Change<br>**-3= decline of >30%**<br>**-2= decline of 10-30%**<br>**-1= decline of <10%**<br>**0= no change**<br>**+1= increase of <10%**<br>**+2= increase of 10-30%**<br>**+3= increase of >30%**	Current amount<br>Specify unit (e.g. number of individuals, encounter rate/km2 or number of groups/km2)	Year for the amount specified in **column 3**	Rate your confidence level for the accuracy of your response<br>**1= very high**<br>**2= high**<br>**3= medium**<br>**4= low**<br>**5= very low**
	Species 1					
	Species 2					
	Species 3					
	Species 4					
**Species richness**				N/A	N/A	

21. Water birds
(a=ducks, b=swan, c=pelicans, d=grebes, e=crane, f=cormorants, g= ibises, h=spoonbills etc.)
Note: As the list here is not exhaustive, please write the name of the species if it is not given in the list.

		1	2	3	4	5
**Species abundance**		Name<br>(write only the letter)	Change<br>**-3= decline of >30%**<br>**-2= decline of 10-30%**<br>**-1= decline of <10%**<br>**0= no change**<br>**+1= increase of <10%**<br>**+2= increase of 10-30%**<br>**+3= increase of >30%**	Current amount<br>Specify unit (e.g. number of individuals, encounter rate/km2 or number of groups/km2)	Year for the amount specified in **column 3**	Rate your confidence level for the accuracy of your response<br>**1= very high**<br>**2= high**<br>**3= medium**<br>**4= low**<br>**5= very low**
	Species 1					
	Species 2					
**Species richness**				N/A	N/A	

22. Migratory birds (e.g. Geese)
Note: As the list here is not exhaustive, please write the name of the species if it is not given in the list.

		1	2	3	4	5
**Species abundance**		Name<br>(write only the letter)	Change<br>**-3= decline of >30%**<br>**-2= decline of 10-30%**<br>**-1= decline of <10%**<br>**0= no change**<br>**+1= increase of <10%**<br>**+2= increase of 10-30%**<br>**+3= increase of >30%**	Current amount<br>Specify unit (e.g. number of individuals, encounter rate/km2 or number of groups/km2)	Year for the amount specified in **column 3**	Rate your confidence level for the accuracy of your response<br>**1= very high**<br>**2= high**<br>**3= medium**<br>**4= low**<br>**5= very low**
	Species 1					
	Species 2					
	Species 3					
	Species 4					
**Species richness**				N/A	N/A	

23. Exotic or non-native bird species (e.g. nandu)
Note: As the list here is not exhaustive, please write the name of the species if it is not given in the list.

		1	2	3	4	5
**Species abundance**		Name<br>(write only the letter)	Change<br>**-3= decline of >30%**<br>**-2= decline of 10-30%**<br>**-1= decline of <10%**<br>**0= no change**<br>**+1= increase of <10%**<br>**+2= increase of 10-30%**<br>**+3= increase of >30%**	Current amount<br>Specify unit (e.g. number of individuals, encounter rate/km2 or number of groups/km2)	Year for the amount specified in **column 3**	Rate your confidence level for the accuracy of your response<br>**1= very high**<br>**2= high**<br>**3= medium**<br>**4= low**<br>**5= very low**
	Species 1					
	Species 2					
	Species 3					
	Species 4					
	Species 5					
**Species richness**				N/A	N/A	

Forest cover change

Please rate the following changes in the past 10 years by writing **-3 if there was a decline of >30%, -2= decline of 10-30%, -1= decline of <10%, 0= No change, +1= increase of <10%, +2= increase of 10-30%, +3= increase of >30%.**

24. *Forest cover* within NP __________ within 3 km from the border of NP __________

25. *Forest degradation* within NP __________ within 3 km from the border of NP __________

26. *Forest fragmentation* within NP __________ within 3 km from the border of NP __________

27. *Wood and shrub land vegetation cover* within NP ________ within 3 km from the border of NP ________

28. *Meadow and grassland vegetation cover* within NP _______ within 3 km from the border of NP _______

29. *Cropland cover* within NP __________ within 3 km from the border of NP __________

30. *Wetland vegetation cover* within NP __________ within 3 km from the border of NP __________

31. *Aquatic vegetation cover* within NP __________ within 3 km from the border of NP __________

32. *Incidence of fires* within the NP __________ within 3 km from the border of NP __________

33. *Logging* within the NP __________ within 3 km from the border of NP __________

34. *Mining* within the NP __________ within 3 km from the border of NP __________

35. How do you rate **the current situation** regarding the **severity** of the following threats to biodiversity conservation in your NP?
Please indicate the ratings of severity by writing: **1 if it is very high, 2= high, 3= moderate, 4= low, 5= very low.**
Also indicate the change in the severity of the threats in the past 10 years by writing: **1 if the severity has increased, 0= about the same or -1= decreased.**

Threat	Severity<br>**1= very high<br>2= high<br>3= moderate<br>4= low<br>5= very low**	Change in severity<br>**1= increased<br>0= about the same<br>-1= decreased**	Is the activity legal?<br>**1= Yes<br>2= No<br>3= Both**	Rate your confidence level for the accuracy of your response<br>**1= very high<br>2= high<br>3= moderate<br>4= low<br>5= very low**
Agriculture and aquaculture				
Invasive species				
Dams and water management				
Fire and fire suspension				
Pollution				
Human intrusion and disturbance (e.g. recreational activities, war, military exercise etc.)				
Transport and service corridors				
Residential and commercial development				
Climate change and sever weather				
Energy production and mining				
Hunting and trapping				
Logging				
Gathering terrestrial plants				
Fishing & harvesting aquatic resources				
Geological events (e.g. volcanos, earthquakes etc.)				
Poisoning or killing animals because of human-wildlife conflict				
Others, specify				

V. Economic benefits to local communities

How do you rate **the current situation** in and around the NP in terms of the following **economic variables**?

Please indicate your ratings by writing: **1 if it is very high, 2= high, 3= moderate, 4= low, 5= very low**.
In addition, please **rate the changes** in the economic variables **in the past 10 years** by writing: **1 if it is increased, 0= about the same or -1= decreased.**

Economic variable	Current situation<br>**1= very high<br>2= high<br>3= moderate<br>4= low<br>5= very low**	Change<br>**1= increased<br>0= about the same<br>-1= decreased**	Rate your confidence level for the accuracy of your response<br>**1= very high<br>2= high<br>3= moderate<br>4= low<br>5= very low**
1. Human population around the NP			
2. Economic benefits to local communities from the NP in terms of income, employment, etc			
3. The visitation rate of the national park by tourists			
4. Please specify the number of tourists that visit the park per year		specify the year: ______	
5. Benefits to local communities through tourism			
6. Local unemployment around the NP			
7. Local housing value around the NP			
8. Legal right to access some resources from the NP			
9. Businesses operate around the NP			
10. Grants to local council/admiration from state or federal government			
11. Tourism tax revenue to the government			
12. Livestock ownership by communities around the NP			

VI. Social impact

1. Attitude

How much do you agree with the following **statements** regarding the **current situation about attitudes** of the local community towards the NP?

Please indicate your agreement by choosing **1 if you strongly agree, 2= agree, 3= slightly agree, 4= slightly disagree, 5= disagree, 6= strongly disagree**.
In addition, please **rate the changes** in the situation of each statement **in the past 10 years** around the NP by writing **1 if increased, 0= about the same or -1= decreased.**

Attitude	Current situation<br>**1= strongly agree<br>2= agree<br>3= slightly agree<br>4= slightly disagree<br>5= disagree<br>6= strongly disagree**	Change<br>**1= increased<br>0= about the same<br>-1= decreased**	Rate your confidence level for the accuracy of your response<br>**1= very high<br>2= high<br>3= moderate<br>4= low<br>5= very low**
1. The local communities have positive attitude towards the NP			
2. The local communities have positive attitude towards the park authorities			
3. The local communities trust the park management and authorities			
4. The local communities would like to protect the wildlife in the NP			
5. The local communities would like the land under NP to be distributed to them			
6. The local communities are satisfied with the management of the NP			
7. The local communities support the protection of the NP			

2. Involvement

How much do you agree with the **following statements regarding the current situation about involvement** of the local community?

Please indicate your agreement by choosing **1 if you strongly agree, 2= agree, 3= slightly agree, 4= slightly disagree, 5= disagree, 6= strongly disagree**.
In addition, please **rate the changes** in the situation of each statement **in the past 10 years** around the NP by writing **1 if increased, 0= about the same or -1= decreased.**

Involvement	Current situation<br>**1= strongly agree<br>2= agree<br>3= slightly agree<br>4= slightly disagree<br>5= disagree<br>6= strongly disagree**	Change<br>**1= increased<br>0= about the same<br>-1= decreased**	Rate your confidence level for the accuracy of your response<br>**1= very high<br>2= high<br>3= moderate<br>4= low<br>5= very low**
1. The local communities are involved in the decision making process of the NP			
2. The distribution of the benefits from the NP is equitable			
3. The local communities are satisfied with the benefits they get from the NP			
4. The cultural and spiritual connections of the local communities to the resources from the NP are taken into account in the NP management decisions			
5. The local communities participate in the protection of the NP			
6. The village chiefs/leaders participate in the decision making process of the NP			

3. Behavioral impact

How much do you agree with the **following statements regarding the activities within the NP**?

Please indicate your agreement by choosing **1 if you strongly agree, 2= agree, 3= slightly agree, 4= slightly disagree, 5= disagree, 6= strongly disagree**.

In addition, please **rate the changes** in the situation of each statement **in the past 10 years** around the NP by writing **1 if increased, 0= about the same or -1= decreased.**

Activity	Current situation<br>**1= strongly agree<br>2= agree<br>3= slightly agree<br>4= slightly disagree<br>5= disagree<br>6= strongly disagree**	Change<br>**1= increased<br>0= about the same<br>-1= decreased**	Rate your confidence level for the accuracy of your response<br>**1= very high<br>2= high<br>3= moderate<br>4= low<br>5= very low**
1. Illegal killing/poaching/poisoning is a severe threat for protected species in the NP			
1a. Which protected species are threatened by illegal killing/poaching/poisoning in the NP?<br>If known, please specify the quantities of the animals poached/killed/poisoned)		Species:	
2. Illegal killing/poaching/poisoning is a severe threat for protected species of the NP, and outside the NP			
2a. Which protected species are threatened by illegal killing/poaching/poisoning outside the NP?<br>If known, please specify the quantities of the animals poached/killed/poisoned		Species:	
3. Illegal logging by local communities is a severe threat in the NP			
4. Illegal mining by local communities is a severe threat in the NP			
5. The local communities comply with the rules and regulation of NP			
6. The local communities encroach into the territories of the NP for agricultural expansion			
7. The local communities encroach into the territories of the NP for livestock grazing			
8. The local communities destroy the park by setting fire			

Additional behavioral questions

9. Could you please estimate the percentage of hunters within the local community around the park?

____

10. How do rate your confidence level for the accuracy of your response in question #11?
1= very high, 2= high, 3=moderate, 4=low, 5=very low

11. Did the proportion of hunters in the local community change in the past 10 years?
1= Yes, increased, 2= yes, decreased, 3= no change, 4= I don't know

12. How would you categorize the motivation for killing animals of the NP?
Please write **1 if it is the main motivation, 2= secondary motivation, 3= minor motivation, 4= not applicable, 5= I don't know.**

Motivations for killing	Categories<br>**1= main motivation<br>2= secondary motivation<br>3= minor motivation<br>4= not applicable<br>5= I don't know**	Please rate the change in the motivation for killing animals in the past 10 years<br>**1= increased<br>0= about the same<br>-1= decreased**	Rate your confidence level for the accuracy of your response<br>**1= very high<br>2= high<br>3= moderate<br>4= low<br>5= very low**
Subsistence (self-consumption)			
Commercial			
Cultural reasons			
Entertainment (e.g. trophy hunt)			
Population control			
Human-wildlife conflict			

VII. Characteristics of the local communities

How much do you agree with the following statements regarding the characteristics of the local communities living around the NP?
Please indicate your agreement by writing **1 if you strongly agree, 2= agree, 3= slightly agree, 4= slightly disagree, 5= disagree, 6= strongly disagree**.

Statement	Response<br>**1= strongly agree<br>2= agree<br>3= slightly agree<br>4= slightly disagree<br>5= disagree<br>6= strongly disagree**
1. The culture of the local community is supportive of nature conservation	
2. The local communities have institutions related to nature conservation	
3. Local communities are willing to participate in nature conservation	
4. Local communities have spiritual/religious connection to nature	
5. I trust the local community that they protect the NP	
6. Hunting is deeply rooted in the culture of local communities	
7. The local communities heavily depend on bushmeat as a source of income	
8. The local communities heavily depend on bushmeat as a source of protein	

VIII. Management capacity and political support

How much do you agree with the following statements?
Please indicate your agreement by writing **1 if you strongly agree, 2= agree, 3= slightly agree, 4= slightly disagree, 5= disagree, 6= strongly disagree**

Statement	Response<br>**1= strongly agree**<br>**2= agree**<br>**3= slightly agree**<br>**4= slightly disagree**<br>**5= disagree**<br>**6= strongly disagree**
1. I get sufficient on job training	
2. I have sufficient resources (financial, human, infrastructure, etc.) for the management of the park	
3. The government is willing to offer support whenever needed	
4. I get sufficient support from the government	
5. I have the power to make the park management decisions	
6. I have the ability to give rewards to subordinates	
7. I have the ability to penalize workers for their inappropriate actions	
8. The government gives priority to biodiversity conservation than economic development	
9. There is independent and strong government office that works for nature conservation in the country	

Thank you very much for your time.
We appreciate your participation in this study!
