# Supplementary material for: Analysis of differences and commonalities in wildlife hunting across the Africa-Europe South-North gradient
Source: PLoS Biol. 2022 Aug 30;20(8):e3001707. doi: 10.1371/journal.pbio.3001707 (PMC9426919; doi:10.1371/journal.pbio.3001707)
Supplement: Attachment — Submitted filename: PBIOLOGY-D-21-01561_R1_reviewer.pdf [file pbio.3001707.s018.pdf]

PLOS Biology

Differences and commonalities in wildlife hunting across the Global South-North gradient

Asynch.CoverPage.ManuscriptDraft

**Common.Text.ManuscriptNumber:**	PBIOLOGY-D-21-01561R1
**Common.SubmissionDetails.FullTitle:**	Differences and commonalities in wildlife hunting across the Global South-North gradient
**Common.SubmissionDetails.ShortTitle:**	Wildlife hunting across the Global South-North gradient
**Common.Labels.ArticleType**	Initial Research Submission
**Common.SubmissionDetails.Keywords:**	Conservation, Protected Area Management, Hunting, Poaching, Culling, Human-wildlife conflicts, Bushmeat, Decolonize Conservation, Commons theory, Conservation interventions, Protected Area Assessment, PA, Hunting pressure, Europe, Africa, Contrasting compa
**Common.SubmissionDetails.Abstract:**	Wildlife hunting is typically studied regionally with a focus on the Global South. However, hunting can also threaten wildlife in the Global North, and little is known about how hunting manifests across the contrasting socioeconomic and ecological South-North gradient. Here, we examine differences and similarities in rudimentary hunting characteristics using face-to-face interviews from 114 protected area managers in 25 African and European countries. Generally, we observe higher illegal, commercial and unsustainable hunting of herbivores in the South, and legal hunting of ungulates and illegal hunting of predators outside parks in the North. Commonalities were found in the increase in conflict-related kills in human-dominated landscapes and beneficial community conditions (e.g. mutual trust resulting from community involvement in park management) leading to decreased illegal hunting. Our results highlight the requirement of collaborative efforts needed for wildlife conservation across actors, while the commonalities may outline universal mechanisms of human cooperation and generalizable pathways within the complex phenomenon of wildlife hunting.
**Common.EditSubmissionSteps.AdditionalInformation:**	
**Common.Text.Question**	**Common.Text.Response**
**Competing Interests**<br><br>You are responsible for recognizing and disclosing on behalf of all authors any competing interest that could be perceived to bias their work, acknowledging all financial support and any other relevant financial or non-financial competing interests.<br><br>Do any authors of this manuscript have competing interests (as described in the PLOS Policy on Declaration and Evaluation of Competing Interests)?	The authors have declared that no competing interests exist

Asynch.CoverPage.PoweredByEM

**If yes**, please provide details about any and all competing interests in the box below. Your response should begin with this statement: *I have read the journal's policy and the authors of this manuscript have the following competing interests:*<br><br>**If no** authors have any competing interests to declare, please enter this statement in the box: "*The authors have declared that no competing interests exist.*"<br><br>* typeset<br><br>This statement is required for submission and **will appear in the published article** if the submission is accepted. Please make sure it is accurate and that any funding sources listed in your Funding Information later in the submission form are also declared in your Financial Disclosure statement.	
**Ethics Statement**<br><br>You must include an Ethics Statement in the Methods section of your manuscript if your study involved:<br>• Human participants<br>• Human specimens or tissue<br>• Vertebrate animals or cephalopods<br>• Vertebrate embryos or tissues<br>• Field research<br><br>**Not including a statement when required will delay the review process.**<br><br>General guidance is provided below. Please consult the guidelines on human subjects research and animal research for detailed instructions.	I confirm that I have read and understood PLOS' requirements for an Ethics Statement.

Asynch.CoverPage.PoweredByEM

**Format for specific study types**<br><br>**Human Subject Research (involving human participants and/or tissue)**<br>• Give the name of the institutional review board or ethics committee that approved the study<br>• Include the approval number and/or a statement indicating approval of this research<br>• Indicate the form of consent obtained (written/oral) or the reason that consent was not obtained (e.g. the data were analyzed anonymously)<br><br>**Animal Research (involving vertebrate animals, embryos or tissues)**<br>• Provide the name of the Institutional Animal Care and Use Committee (IACUC) or other relevant ethics board that reviewed the study protocol, and indicate whether they approved this research or granted a formal waiver of ethical approval<br>• Include an approval number if one was obtained<br>• If the study involved *non-human primates*, add *additional details* about animal welfare and steps taken to ameliorate suffering<br><br>**Field Research**<br><br>Include the following details if this study involves the collection of plant, animal, or other materials from a natural setting:<br>• Field permit number<br>• Name of the institution or relevant body that granted permission<br><br>Please check the box to confirm your understanding of this policy, then read and agree to one of the statements.	
Please respond:<br>Common.Text.AsFollowUpTo	My study requires an ethics statement. I confirm I have included my statement in the Methods section of my uploaded manuscript file.
**Data Availability**	No - some restrictions will apply

Asynch.CoverPage.PoweredByEM

PLOS journals require authors to make all data underlying the findings described in their manuscript fully available, without restriction and from the time of publication, with only rare exceptions to address legal and ethical concerns (see the PLOS Data Policy and FAQ for further details). When submitting a manuscript, authors must provide a Data Availability Statement that describes where the data underlying their manuscript can be found.<br><br>Your answers to the following constitute your statement about data availability and will be included with the article in the event of publication. **Please note that simply stating 'data available on request from the author' is not acceptable.** ***If, however, your data are only available upon request from the author(s), you must answer "No" to the first question below, and explain your exceptional situation in the text box provided.***<br><br>Do the authors confirm that all data underlying the findings described in their manuscript are fully available without restriction?	
Please describe where your data may be found, writing in full sentences. **Your answers should be entered into the box below and will be published in the form you provide them, if your manuscript is accepted.** If you are copying our sample text below, please ensure you replace any instances of XXX with the appropriate details.<br><br>• If your data are all contained within the paper and/or Supporting Information files, please state this in your answer below. For example, "All relevant data are within the paper and its Supporting	The data are confidential so data will be available by request, or anonymized so that individual national parks or individuals will not be identifiable.

Asynch.CoverPage.PoweredByEM

Information files.”<br>• If your data are held or will be held in a public repository, include URLs, accession numbers or DOIs. For example, “All XXX files are available from the XXX database (accession number(s) XXX, XXX)." If this information will only be available after acceptance, please indicate this by ticking the box below.<br>• If neither of these applies but you are able to provide details of access elsewhere, with or without limitations, please do so in the box below. For example:<br><br>“Data are available from the XXX Institutional Data Access / Ethics Committee for researchers who meet the criteria for access to confidential data.”<br><br>“Data are from the XXX study whose authors may be contacted at XXX.”<br><br>* typeset	
Additional data availability information:	Tick here if your circumstances are not covered by the questions above and you need the journal’s help to make your data available.

Asynch.CoverPage.PoweredByEM

Manuscript Click here to access/download;Manuscript;Manuscript_PB_25.06.2021.docx

Differences and commonalities in wildlife hunting across the Global South-North gradient

Mona Bachmann[1,2*], Lars Kulik[1], Tsegaye Gatiso[3], Martin Reinhardt Nielsen[4], Dagmar Haase[2,5], Marco Heurich[6,7,] Ana Buchadas[2,8], Lukas Bösch[9], Dustin Eirdosh[1,], Andreas Freytag[10], Jonas Geldmann[11], Arash Ghoddousi[2], Thurston Cleveland Hicks[12], Isabel Ordaz-Németh[1], Siyu Qin[2], Tenekwetche Sop[1], Suzanne van Beeck Calkoen[6,13], Hjalmar Kühl[1,14]

1. Max Planck Institute for Evolutionary Anthropology, Deutscher Platz 6, D-04013 Leipzig, Germany
2. Geography Department, Humboldt-University Berlin, Unter den Linden 6, D-10099 Berlin, Germany
3. Institute for Food and Resource Economics, Faculty of Agriculture, Bonn University, Nussallee 21, D-53115, Bonn
4. Department of Food and Resource Economics, University of Copenhagen, Rolighedsvej 23, DK-1958 Frederiksberg Copenhagen, Denmark
5. Helmholtz Centre for Environmental Research (UFZ), Leipzig, Germany, Permoserstraße 15 D-04318 Leipzig, Germany
6. Chair of Wildlife Ecology and Management, Albert Ludwigs University Freiburg, Tennenbacher Straβe 4, D-79106 Freiburg, Germany Faculty of Environment and Natural
7. Faculty of Applied Ecology, Agricultural Sciences and Biotechnology, Institute for forest and wildlife management, Campus Evenstad, NO-2480 Koppang, Norway
8. Integrated Research Institute on Transformations of Human-Environment Systems (IRI THESys), Unter den Linden 6, 10099 Berlin
9. Institute for Sociology, University Leipzig, Beethovenstr. 15, D-04107 Leipzig, Germany
10. Faculty of Economics and Business Administration, Friedrich Schiller University Jena, University of Stellenbosch; CESifo Research Network , Carl-Zeiss-Str. 3, D-07743 Jena, Germany
11. Center for Macroecology, Evolution and Climate, Globe institute, University of Copenhagen, DK-2100 Copenhagen
12. Faculty of Artes Liberales, University of Warsaw, Warsaw, Poland, Ulica Nowy Świat 69, PL–00-046 Warsaw (Poland)
13. Department of Visitor Management and National Park Monitoring, Bavarian Forest National Park, Freyunger Straβe 2, D-94881 Grafenau, Germany
14. German Centre for Integrative Biodiversity Research (iDiv) Halle-Jena-Leipzig, Puschstrasse 4 D-04103 Leipzig, Germany

* Corresponding author: mona_bachmann@eva.mpg.de

DECLARATION OF INTEREST STATEMENT:

The authors have no competing interests to declare

FUNDING SOURCES:

This project was hosted by the Max Planck Institute for Evolutionary Anthropology and funded by the German Center for Integrative Biodiversity Research (iDiv; DFG FZT 118) and the Robert Bosch Foundation.

Acknowledgements

Our gratitude goes to all PA managers and stuff who participated in our survey and filled in our questionnaire (full list is given in Supplementary Information). Furthermore, we thank all who participated in the NGO survey: Zuzana Záborská (Regional Tourism Organization Slovenský raj & Spiš), Karol Kaliský (Arolla Film), LZ VLK, Viliam Bartuš (WOLF Forest Protection Movement, Eastern Carpathians tribe), Hnutí DUHA Olomouc, Tomasz Pezold Kneževi (WWF Poland, IUCN WCPA), NHF, O. Ionescu (Transylvania University), Florin Stoican (Asociatia Kogayon), Andrei Szabo (Asociatia Euroland Banat), Asociatia Salvati flora si fauna Deltei Dunarii, Propark-Fundatia pentru Arii Protejate, Joseph Kouassi, Yves Kablan, Prof. Emmanuel Danquah (Department of Wildlife and Range Management, FRNR, KNUST, Kumasi, Ghana), Angedakin Samuel, and the CTPH Conservation Through Public Health. We also would like to thank, Adam Bohdan, Sarah Bunel, Hayfe Chamkhi, Martina Duskova, Vidrige Kandza, Elysée Mbaygone, Nyakoojo Moses, Terence Fuh Neba, Peter Sabo, Clement Tweh, Andrada Vaidos, Mercy Wambui and others for their support in collecting the data. We gratefully acknowledge the financial support obtained for the study from the German Center for Integrative Biodiversity Research and the Robert Bosch Foundation.

Author contributions

All authors equally contributed to the paper. M.B, H.K, T.G., M.R.N., D.H., M.H. conceptualized the study and carried out initial planning. L.B. and M.B. carried out the statistical analysis. The first draft was prepared by M.B., which was revised by T.G., M.R.N., D.H., A.B., L.B., D.E., A.F., J.G., A.G., M.H., T.C.H., I.O., S.Q., T.S., S.V.B.C., and H.K. All authors reviewed and contributed to a final draft and approved the final version for publication.

Abstract:

Wildlife hunting is typically studied regionally with a focus on the Global South. However, hunting can also threaten wildlife in the Global North, and little is known about how hunting manifests across the contrasting socioeconomic and ecological South-North gradient. Here, we examine differences and similarities in rudimentary hunting characteristics using face-to-face interviews from 114 protected area managers in 25 African and European countries. Generally, we observe higher illegal, commercial and unsustainable hunting of herbivores in the South, and legal hunting of ungulates and illegal hunting of predators outside parks in the North. Commonalities were found in the increase in conflict-related kills in human-dominated landscapes and beneficial community conditions (e.g. mutual trust resulting from community involvement in park management) leading to decreased illegal hunting. Our results highlight the requirement of collaborative efforts needed for wildlife conservation across actors, while the commonalities may outline universal mechanisms of human cooperation and generalizable pathways within the complex phenomenon of wildlife hunting.

Introduction:

Biodiversity loss and the rise of zoonotic diseases have reinforced global calls to mitigate the unsustainable exploitation of wildlife [1]. Scientific activity and international scrutiny commonly focus on the Global South [1], where poverty-driven and illegal hunting leads to dramatic declines in mammal and bird populations [2–4]. In the more affluent nations of the Global North, returning and growing populations of large herbivores and predators such as grey wolves (*Canis lupus*) and European bison (*Bison bonasus*) are symbols of conservation success [5]. Nevertheless, here too, both illegal and legal intensive hunting limits recoveries, and it threatens and has even eradicated populations [5–8]. For many protected predators, illegal persecution remains a major cause of mortality and enjoys social acceptance in some parts of Europe [5,6].

Despite these similarities, most research remains geographically "segmented" in a firmly anchored North-South binary [2,3,9], and little is known about how hunting unfolds across the Global South-North gradient. The function of hunting can extend far beyond the traditional provision of meat; it underlies economies and cultures, serves recreation, and aims at biodiversity conservation or the eradication of

conflict-prone species [10]. These human-wildlife relationships are regionally manifesting phenomena shaped by a complex set of social and cultural constructs, moral values, material realities, and political and historical characteristics that vary over time, locations, and human communities [11,12]. Consequently, conservation measures must integrate local diversity, and large-scale assessments over a diverse context may provide few general insights. Alternatively, has hunting played an essential role in human evolution and is practised in most modern societies, from hunter-gatherers to both rural and urban dwellers and across all social classes [10,12]. Based on this extended evolutionary perspective, synthesis along a diverse gradient may make it possible to identify deeper underlying mechanisms that outline generalizable pathways within the exceedingly complex phenomenon of wildlife overexploitation.

The Global South-North divide describes a recent historical division into colonized and colonial states. Even today, one-fourth of the world's population in the Global North controls four-fifths of global income [13]. Biodiversity appears in different stages, as more wildlife species have been exterminated in the Global North [5,14]. Nevertheless, the conservation and science sectors are largely dominated by Western actors, concepts and worldviews [15,16]. Both double standards and long-standing inequalities lead to accusations of cultural imperialism and neo-colonialism in the Global South [15,17–20]. A firm North-South binary can foster ethnocentric representations of the Global South as the “other” of the norm [21], which has a long continuity and legitimizes controversial conservation approaches that would be unacceptable in the Global North, such as “fortress protection”, “green militarization” and “shoot-on-sight policies” [15,22,23]. Implicit biases about poverty, inequality, historical grievances, and colonial and racist discourses may further still shape perceptions of hunting and poaching [22]. By contrast, comparisons across contrasting environments further our understandings of the differences, commonalities and universal mechanisms within a phenomenon and help to reduce the complexity through generalization against the comparative horizon of the contextual scale [24,25]. Such a global perspective on the processes around hunting across the Global South-North gradient and its contrasting economic and political contexts and path dependencies allows us to outline local and global processes and corresponding problems and possible solutions. Ultimately, understanding the local versus generalizable dimension of hunting fosters cross-contextual learning and allows a more equal conservation debate between actors in the Global North and South.

Here, we aim to understand the differences and commonalities in hunting across the Global South-North gradient regarding four basic characteristics: 1) “why”, 2) “what”, and 3) “where” people hunt, as well as 4) how unsustainable hunting can be mitigated. We focus on protected areas (PAs) and adjacent areas

as an interface of biodiversity protection and resource use. PAs are a tool for mediating between people's needs and natural resources and a cornerstone of biodiversity conservation [26]. Nevertheless, illegal exploitation within parks and human-wildlife conflicts (HWCs) beyond administrative boundaries pose a major risk to biodiversity [2,3,27]. PAs allow hunting for wildlife management, and it is usually subject to regulations designed to guarantee sustainability. However, legal hunting can also alter wildlife movements, shape life histories by artificial selection, and decimate populations to a socially acceptable but ecologically fragile minimum [7,28,29]. We aim to conduct a holistic assessment beyond a simplistic binary view of "legal and sustainable" vs. "illegal and unsustainable" to understand the diverse manifestations/realties of hunting and its varying impacts on wildlife. Therefore, we define hunting as the totality of activities involved in the management and pursuit of wildlife [10]. Unsustainable hunting encompasses activities identified by PA managers as not being in harmony with the PAs, such as removals exceeding population growth and disruptive hunting practices.

Current global PA assessments capture hunting as one homogeneous threat [30]. To obtain a more detailed assessment, we conducted face-to-face interviews 114 PA managers in 25 African and European countries (Fig. 1a, S1 Table). To understand 1) "why", we examined a) the predominant function of hunting, divided based on sustainability science into "economic" (subsistence, commercial hunting), "sociocultural" (non-market cultural, social, recreational aspects), and "ecological" (populations management, killing due to HWCs) [10], and b) the impact of "hunting" as a human-wildlife interaction and more specifically HWCs . To answer 2) "what", we compiled the targeted species and examined whether ecological parameters such as the trophic level influence the likelihood of being affected by hunting. To understand 3) "where", we examined illegal hunting and whether this threat to wildlife tends to be located more within or outside administrative PA boundaries. We approximated the Global South-North gradient with the Human Development Index (HDI) and included regional human population density, which is known to affect human-wildlife relationships [31]. To investigate 4) how unsustainable hunting can be mitigated, we included an index reflecting local community characteristics (nature-friendly cultures, attitudes, relationships) and implemented conservation interventions (protection and community based) (for the construction of the indices, see S2 Table). With this extraordinary juxtaposition of differences and unifying elements, we aim to identify regional challenges and general mechanisms that can ultimately help to develop effective and just wildlife conservation measures across this diverse gradient.

Results:

We collected comprehensive information through face-to-face interviews with managers of 114 protected areas in ten European (n=48) and fifteen African countries (n=66) (Fig. 1a, S1 Table). Most PAs fell into IUCN management category II (69%; Africa (65%), Europe (72%)), while the remaining PAs were in IUCN categories III-VI, or had no assigned category (see S1 Table). African PAs were larger on average (2,253 km^2 (median=973 km^2, range= 26-21,812 km^2) than European PAs (570 km^2, median=330 km^2, range=13-3,446km^2), while European PAs were on average older (45 years, median=36, range=16-109 years) than African PAs (36 years, median=32 years, range=8-87 years).

The threat from hunting was not confined to the Global South. Of the 114 interviewed PA managers, only eight of the 48 (17%) European and five of the 66 (8 %) African parks did not list any species threatened by hunting (Fig. 1b). African parks listed on average significantly more species threatened (3.6 ± 2.8) than European parks (2.2 ± 1.9) (t = 3.001, p-value = 0.003). When we differentiated between sub-regions, we found a more heterogeneous picture with the least number of species listed in Southern Africa and Western Europe (Fig. 1b). Although most parks fell under the IUCN management category II (S1 Table), legal hunting for population control was more common in Europe (25 of 48 PAs, 52%) than Africa (3 of 66 PAs, 4.55%), with an average offtake of 389 ± 1189 animals/park (1.08 ± 1.72 animals/km2) in Europe and 17 ± 133 animals/park (0.04 ± 0.26 animals/km^2) in Africa. The average offtake was lower when at least one of large predators were present (mean=0.36 animals/km^2) compared to their absence (mean=0.74 animals/km^2). However, these differences were not significant (t= 1.11, p-value = 0.272).

Figure 1 **Overview of sample locations, legal setting and threatened species** a) Map of sampled PAs (yellow dots) and HDI per surveyed country. The Global South-North divide in our data is at >0.75 defined as so-called Global North (Europe, green) and <0.75 defined as Global South (Africa, purple-blue), b) Violin plots displaying the absolute number of species threatened by hunting listed by PAs across regions, black dots=number of listed threatened species per park, blue and red dot= mean per region, blue line= interquartile range, each side of the blue line is a kernel density estimation of the data. c) legal offtake per PA in absolute numbers (left) and numbers per km^2 per continent (right).

Why people hunt

Regarding the question of why people hunt, our Bayesian regression models revealed variations in the prevailing function of hunting across the South-North gradient. Over the observed range of the HDI, we found that our three functions of hunting show different trajectories. The "ecological function" revealed slightly stronger prevalence with an increasing HDI. This is even more pronounced for the category of

“social function”. The “economic function” revealed a completely different pattern, with clearly decreasing hunting pressure with increasing HDI (Fig 2a, S4 Table). Effects were non-linear with an observed threshold and division at HDI>0.75 (European levels) (Fig 2a, HDI>0.75, grey vertical line). The average index across all functions remained relatively equal over the South-North gradient (Fig. 2a, horizontal dashed black line).

Regarding human-wildlife-interactions, 37.5% (42 of 112) of park managers reported high threat levels to wildlife through hunting due to unsustainable hunting offtake rates or practices. Increasing HDI levels over the South-North gradient were the strongest predictor reducing the probabilities of a high threat through hunting (Fig. 2b, S4 Table, estimate = -1.58, CI = [-2.59, -0.65]). The point probability halved when reaching Europe (HDI>0.75) (Fig. 2b), which suggests that unsustainable hunting also occurred in Europe, but to a lesser extent. Rankings for high hunting pressure were more evenly distributed across the gradient, while absences were more frequent with higher HDI levels. Wildlife abundances were slightly negatively associated with high hunting threats (estimate = -0.76, CI = [-1.69, 0.06]). The strong effect of HDI remained when only considering cases of illegal hunting (S6 Table, HDI: estimate = 1.7, CI = [-2.79, -0.62]).

Considering the particular aspect of HWCs, including all species, 20.6% (20 of 97) of the parks reported high threat levels through HWC-driven hunt, whereby only two (0.1%) high ratings referred to a legal setting. These high ratings referred four times (9.6 %) to legal hunt, 30 times (71.4%) to illegal hunt, and eight times (19%) to both because the effects were inseparable (Fig. 2b). The human population density was the strongest predictor, increasing the prevalence of HWC (Fig.2c, S7 Table, estimate = 0.96, CI = [-0.06, 2.04]). Our control predictor of decreasing wildlife abundances which was measured over the past ten years (S2 Table) was related to higher levels of HWC (estimate = -0.59, CI = [-1.57, 0.37]). We found similar effects for the two models (hunting, HWCs) irrespective of the grouping (considering levels of "high" and "very high" or "moderate", "high" and "very high") (S5 Table, S8).

Figure 2. **Why people hunt:** a) Functions of hunting as an interaction between HDI and the hunting functions, ecological (population control, human-wildlife conflicts), social (entertainment, socio-cultural hunting), economic function (subsistence, commercial hunting). The category "ecological function" and “social function” revealed larger hunting pressure with an increasing HDI, while the economic category revealed a clearly decreasing hunting pressure, the functions split at the Global South-North divide (grey dashed line), black dotted line= mean. Human-wildlife-interactions b) hunting: the probability of a high threat through hunting decreased along the South-North gradient, c) human-wildlife conflict-driven hunt: the probability of high threats of human-wildlife conflicts in relation to human population density (inhabitants/km^2) revealed an increasing trajectory with human densities. The dashed line depicts the expected mean of the predicted posterior distribution; the coloured areas depict the 33% and 66% credibility intervals. Transparent points are binned probabilities. The size of the bubbles corresponds to the respective number of PAs. Filled points: original data, vertical grey dashed line: Global South-North divide of our data (0.75).

What people hunt

Species were listed as threatened by illegal hunting (including all lethal interactions) 316 times, belonging to 117 different species (Fig. 3a). Within the guilds found on both continents, herbivores were more threatened by illegal hunting (including HWCs) in Africa and predators more in Europe (Fig. 3a). Overall, wild boars (*Sus scrofa*) and red deer (*Cervus elaphus*) were the most legally hunted species (Fig. 3c). Unfortunately, we received insufficient data for the comparison of the overall extracted biomass, legally or illegally hunted in Europe and Africa. The probability of a predator (predatory mammals, birds of prey) being threatened by hunting increased with higher HDI scores compared to non-predatory mammals and birds (Fig. 3b, S9 Table, estimate = 1.22, CI = [-0.11, 2.36]). A higher current threat to predators was associated with an increase in their abundance, which was measured over the past ten years (estimate = 0.57, CI = [-0.31, 1,49]).

Figure 3 **What people hunt**: a) The absolute number of threatened species per guild listed by park managers. In Africa more herbivorous species and in Europe, more top mammalian predators and raptors were named. Africa=violet, Europe=turquoise, b) the probability that a predatory species (top, small to medium predators, raptors) compared to non-predatory species (all others) were threatened showed an increasing trajectory over the South-North gradient (i.e. HDI). The dashed line depicts the expected mean of the predicted posterior distribution. The coloured areas describe the 33% and 66% credibility intervals. Transparent points are binned probabilities. The size of the bubbles corresponds to the respective number of PAs. Filled points are original data. The vertical grey dashed line is the Global South-North divide of our data (0.75), c) the legal offtake per species and square kilometre across European parks (left, turquoise) and across African parks (right, violet).

Where people hunt and how unsustainable hunting can be mitigated

In terms of where illegal hunting activities were spatially located, park managers distinguished in 206 of the 316 mentions whether species were more endangered by illegal hunting (including all lethal interactions) inside the park territory or when ranging beyond administrative borders. Here, 63.1% of the species (130 of 206 cases, 56 distinct species) were threatened more when ranging outside borders, and 36.9% (76 of 206 cases, 45 distinct species) were threatened more within the park territory (Fig. 4a). The 21 species listed as threatened only outside of PAs were mainly raptors (7) and mammalian predators (7). In Europe, predators were particularly threatened outside of PAs, and in Africa herbivores were the most threatened guild, inside and outside of PAs (Fig. 4a).

Our models revealed a declining threat inside parks through illegal hunting at an increasing rate when approaching HDI>0.75 (Europe) (Fig. 4b, S10 Table, HDI: estimate = -1.44, CI = [-2.46, -0.46]). Threats also declined outside of PAs, albeit to a lesser extent (Fig. 4c, S11 Table, HDI: estimate = -0.95, CI = [-1.94, -0.01]. The data distribution suggests that particularly the absence of the threat increased with HDI.

Protection efforts, including ranger patrols, a permanent research station, and buffer zones, were weakly associated with a lower threat within PAs (estimate = -0.58, CI = [-1.51, 0.28]).

Supportive local community characteristics were linked to lower threats through illegal hunting to animals within (Fig. 5a, S10 Table, estimate = -0.99, CI = [-1.91, -0.12]) and outside of PAs (Fig. 5e, S11 Table, estimate = -0.89, CI = [-1.83, 0.01]). When we included the components of the "local community characteristics" index separately in the models, all components showed effects. Here, mutual trust between park management and communities was linked to lower threat probabilities inside (Fig. 5c, S12 Table, estimate = -1.26, CI = [-2.23, -0.34]) and outside of PAs (Fig. 5g, S13 Table, estimate = -0,82, CI = [-1.73, 0.03]).

Likewise, conservation-friendly attitudes reduced threat probabilities inside (Fig. 5b, S14 Table, estimate = -0,82, CI = [-1.72, 0.02]) and outside of PAs (Fig. 5h, S15 Table, estimate = -0,71, CI = [-1.72, 0.27]). Similar effects were observed for conservation-friendly local cultures for inside (Fig. 5d, S16 Table, estimate = -0,7, CI = [-1.6, 0.14]) and outside of parks (Fig. 5h, S17 Table, estimate = -0,88, CI = [-1.82, -0.03]). Remarkably, in contrast to the effects of HDI, the effects here were less pronounced within rather than outside of parks. The models including only mutual trust best explained the results (S18 Table).

When examining whether specific community interventions can foster such beneficial community conditions, the only predictor that showed an effect was "scale of inclusion of local communities" (Fig 5i, S19 table, estimate = 1.4, CI = [-0.21, 2.77]) (alongside' provision of benefits to communities", "implementation of livelihood projects", and "awareness creation").

Figure 4 **Where people hunt:** a) The absolute numbers of listed species of the PA threatened by illegal hunting within the administrative borders versus outside the park borders revealed higher threats for predators outside borders in Europe (darker colours) and higher threats for herbivores inside and outside parks in Africa (lighter colours). The probability that wildlife is threatened by illegal hunting (killing/hunting/poisoning), b) within the park boundaries and c) outside the park boundaries decreased over the South-North gradient. Outside parks, the probability decreased to a lower extent. The data distribution revealed decreasing threat with higher HDI values. The dashed line depicts the expected mean of the predicted posterior distribution. The coloured areas depict the 33% and 66% credibility intervals. Transparent points are binned probabilities. The size of the bubbles corresponds to the respective number of PAs. Filled points are original data. Vertical grey dashed line is the Global South-North divide (HDI of 0.75).

Figure 5 **Where people hunt and what mitigates unsustainable hunting.** Left panel: Probability of high threat to wildlife through illegal hunting (killing/hunting/poisoning) inside PAs related to a) community characteristics and the separate components of the index community characteristics: b) attitudes of local communities towards conservation, c) mutual trust levels between park management and organizations, d) conservation-friendly local culture. Right panel: Threat levels outside park boundaries across e) community attributes, f) attitudes, g) mutual trust levels, h) local culture. Threat levels decreased equally outside and inside parks, while the effects were more pronounced outside parks. The models, including mutual trust, best explained the results i) the scale of inclusion of local communities in decision-making showed a positive relationship with the index community characteristics. The "scale of inclusion" was alongside' provision of benefits to communities", "implementation of livelihood projects", and "awareness creation" the only predictor which showed an effect. The dashed line depicts the expected mean of the predicted posterior distribution. The coloured areas are the 33% and 66% credibility intervals. Transparent points are binned probabilities. The size of the bubbles corresponds to the respective number of PAs. Filled points are original data.

Discussion:

Although comparative studies are rare, hunting in the Global South is often portrayed as fundamentally different from hunting in the Global North [22]. Our results confirm differences in all basic hunting characteristics. Towards the North, economically motivated and highly unsustainable hunting threatening herbivores within and outside parks has declined, while the legal hunting of ungulates has increased, and the threat to wildlife beyond park boundaries has persisted, albeit to lower extents, and has simultaneously increased for predators. Remarkably, the commonalities found were mainly related to factors mitigating illegal hunting, such as the favourable characteristics of local communities, including conservation-friendly attitudes, cultures, and, in particular, trust relationships associated with higher community involvement. Nevertheless, they did not compensate for the strong South-North effect. Moreover, conflict-driven hunting showed an increase with higher human populations along the gradient.

Differences across the Global South-North gradient

Considering why people hunt, we observed a prevailing economic function in Africa (Fig. 2a), reflecting the needs of those whose livelihoods depend on the consumption and trade of wild meat [10], despite being mainly classified as illegal (Fig. 1c). In the North, we observed a division into an ecological function, which primarily reflects state-regulated lethal wildlife control, and a high social function, which reflects the recreational, social, and cultural needs of hunters and their associations (Fig. 1c), with both functions being mainly legal. The prevailing social function is remarkable because hunting in PAs is considered an ecological necessity [10]. The presence of large predators showed no effect on offtake rates. Some parks, however, adapted to the return of large predators, such as the Czech Šumava National Park, which declared no-hunting zones after the arrival of a wolf pack [32]. Nevertheless, current ungulate management in Europe is determined by the national and socio-political context rather than by ecological parameters or International Union for Conservation of Nature (IUCN) guidelines, and our results emphasize the call for an integrated European management policy [33].

The results further revealed that the steep HDI gradient along the Global South-North axis drove unsustainable hunting, likely explained by the primary economic purpose, driven by reliance on wildlife products [4], combined with illegal and, thus, unregulated hunting. Uncontrolled and trade-driven hunting is the greatest threat to biodiversity [34] and increases the risks of disease transmission during sale, transport, and consumption [35], which confirms the high importance of improving the living

conditions in low-HDI countries. Nevertheless, unsustainable hunting also occurred in Europe, albeit to a lesser extent, and partly involved legal settings (Fig. 2b). In Slovakia, for example, hunting associations determined shooting quotas, and in Austria, hunting laws prohibited the use of silencers next to bird breeding areas. Overall, the continental differences in the legal frameworks for hunting within the same IUCN management categories are striking and might be rooted in colonialism, where African hunters were stigmatized, criminalized and displaced from PAs [22,23] and the insufficient co-determination of local actors is still criticized by decolonization movements [19].

In terms of what people hunt, high rates of ungulates were legally hunted in Europe (Fig. 3c). Illegal hunting entailed more predators in the North and more herbivores in the South (Fig. 3a). The latter might be related to a higher demand for wildlife products suitable for consumption and commercialization [36]. Moreover, many vulnerable species, like large herbivores such as the European bison (*Bison bonasus*), were hunted to local extinction, resulting in more resilient species represented in Europe's biodiversity [31,37,38]. The greater endangerment of predators in the North (Fig. 3b), typically victims of HWCs, is counterintuitive if considering that the costs of coexistence with wildlife are disproportionately higher in the Global South [20]. However, the extent of conflict is rarely proportional to the actual damage because cultural and social perceptions of potential threats and the values associated with wildlife are decisive [9]. The social function of hunting in the North (Fig. 2a) can encourage the persecution of predators if it is associated with enjoyment and status or competition over common prey [6,9]. Moreover, the ecological function and, thus, the support of lethal wildlife controls are typically linked to higher wildlife value orientations of domination, which are widespread in Europe [39]. Here, human needs are strongly prioritized over the perceived needs of wildlife, while in a mutualist wildlife-value orientation, both are considered to be more equally important [40]. These value orientations can affect coexistence; for example, the reintroduction of wolves was more successful in U.S. states with prevailing mutualistic value orientations [40]. The results further revealed that predators from growing populations were increasingly targeted (S9 Table), presumably in areas where people have grown unaccustomed to their presence [6,27]. Persecution does not yet seem to have decimated the overall predator populations. Nevertheless, single populations, such as lynxes in the Bohemian Forest Ecosystem, are endangered [5,6].

Regarding where people hunt, our results showed a high threat to wildlife from illegal hunting of any kind inside and outside PAs for the Global South and outside PAs in the North (Fig. 4b,c). Alternative

livelihoods available when the HDI rises may outweigh the benefits of illegal and, thus, risky hunting inside PAs. Additionally, African PAs lack financial resources for effective protection efforts inside parks [41], and they may be even weaker outside parks. Moreover, hunting concessions open to the trophy hunting of protected species often neighbour national parks [42]. For example, male lions from the park interior frequently reoccupy territories emptied by trophy hunting outside the park, precipitating declines across the whole area [43,44]. In Europe, PAs are also embedded in hunting areas, and they provide, for example, vital source areas for lynx kittens to mature safely, but illegal killings of dispersing subadults have a strong sink effect on the species [45]. These killings are often cryptic, with carcasses buried or animals poisoned, and the risks of being caught are low [46]. Migratory species and, as shown, predators with large home ranges (Fig. 4a) (i.e., 917 km^2 for male Eurasian lynxes [47]) are particularly affected. This confirms that border areas represent population sinks for predators, even though they enjoy mostly legal protection [6,45,48]. European parks are even smaller, and their role is limited, as predators largely exist outside PAs [5,45] and predators that range widely are most likely to disappear from small reserves, irrespective of their population size [48]. This underscores a general shortcoming of the PA concept, as parks are not closed systems but are embedded in ecological, economic, and social landscapes [49]. Therefore, we need landscape-connected PAs and proactive strategies enabling coexistence, as the survival of returning wildlife in the North will ultimately depend on people's willingness to share landscapes [8,50].

Commonalities across the Global South-North Gradient

Despite all the socioeconomic and environmental contrasts, we found common ground. Along the gradient, human density on the regional axis was associated with increased killings due to HWCs, including all species (Fig. 2b). This finding may be a simple function of increased encounters or competitive pressure between wildlife and growing numbers of humans. Likewise, wildlife adapt their behaviours when less habitat or natural dietary items are available, which can increase predation on livestock, for example [51]. Over millennia, growing populations of humans and their livestock have replaced wild animal populations [31]. The traditional Western PA concept is inherently rooted in an HWC, assuming that wildlife and humans cannot coexist [23]. Notably, we found no effects of human densities on predators. Indeed, all four large European carnivore species, brown bears, lynxes, wolves, and wolverines, persist in human-dominated landscapes (i.e., wolves: 36.7 ± 95.5inhabitants/km^2) [5]. Coordinated legislation across Europe, context-specific management practices, and institutional

arrangements enabled this conservation success [5,8], ultimately outweighing this archetypal human-wildlife relationship.

Considering what mitigates unsustainable hunting, we found only a modest effect of protection-based conservation that marginally decreased threats to wildlife through illegal hunting inside PAs (S10 Table). This finding could confirm that PAs are increasingly struggling to protect biodiversity from the pressures exerted by the larger context [2,49]. Alternatively, it could suggest reversed causalities, where more interventions are applied when wildlife is already threatened and the effects of threat mitigation from high original levels are difficult to detect. Hence, further interpretations require more detailed investigations.

Regarding what mitigates the threat to wildlife from illegal hunting inside and, in particular, outside parks, another identified commonality is the effect of supportive local community conditions. After conservation-friendly attitudes and cultures, mutual trust between management and communities showed the strongest effect (Fig. 5c,g). Trust has proven central to biodiversity conservation [52], such as to the success of PAs [53] or carnivore protection [54]. Trust can be broken when some feel that other parties' interests are being promoted at their expense [52]. This typically correlates with varying perceptions, uses, priorities, and impacts of wildlife or existing power gradients such as rural-urban or Global South-North [15,52,54]. Important trust-building mechanisms consist of transparent and fair decision-making processes and the ensured participation of all parties [52,55]. Notably, the inclusion of local communities in management contributed to supportive community conditions (Fig. 5i). Externally imposed rules can lead to animosity towards conservation or even forms of "resistance poaching" purposely targeting key conservation species [12,15]. Conversely, well-implemented inclusive approaches benefit the social, ecological, and ecological outcomes of PAs [53] by ensuring equity and trust, reinforcing positive attitudes, and considering the plurality of cultural values associated with nature [56].

Generalizable principles, universal pathways

Comparisons against a contrasting background help synthesize knowledge by explaining differences and commonalities as well as universal mechanisms through conceptualizations against the backdrop of a comparative horizon [24,25]. Above, we linked our results by proximal causes, which can be adaptive in the present context. However, ultimate causes related to our history as a social species may also explain commonalities while offering solutions [55,57]. Ostrom and colleagues have identified minimal

conditions, constituting core design principles, that must be met to enable sustainable resource use, rooted in evolutionary aspects of human cooperation [55,57]. Mutual trust, which is one central finding (Fig. 5c,g), is essential for any setting where parties must renounce immediate individual benefits for the long-term good of the whole, such as conservation, and an emergent property of functioning arrangements [55]. Threats beyond PA boundaries (Fig. 4b,c) show conflicts that occur because people and wildlife follow different system boundaries [27]. Therefore, the boundaries around the community of users and the resource itself must be clearly delineated [55,57]. Stronger social boundaries through shared values, rules, or goals can outweigh fuzzy geographic boundaries not respected by mobile wildlife [58], which might explain why beneficial community conditions reduced threats to wildlife, particularly beyond borders (Fig. 5a,e). Furthermore, as another key finding (Fig. 5i), the inclusion of all relevant group members through the right to organize and create rules and decisions of their own, ideally by consensus, is vital for collaboration [55,57]. Inclusive approaches enable local adaptation and trigger intrinsic incentives since people resent being told what to do but work hard to meet agreed-upon goals [55,57]. Finally, parks were largely influenced by factors beyond the control of managers (i.e., HDI, population densities, threat beyond borders); thus, they are parts of larger systems requiring collaboration across multiple scales and levels [57]. For instance, pan-European, regional, and local networks have enabled the return of long-extirpated wildlife to Europe [5,8]. Critically, some arrangements are poorly aligned. Northern countries struggled to protect predators (Fig. 3b), controlled their populations within PAs (i.e., Sweden) (S20 Table), and opposed rewilding while simultaneously advocating for coexistence abroad with costs disproportionately borne by the rural Global South [20]. Likewise, apparent continental differences in legal settings (Fig. 1c, Fig. 2a, Fig. 3c) may still be relics of colonial times and indicate a lack of local participation in the South. Furthermore, exclusionary and militarized approaches are becoming normalized among some conservation agents and sub-Saharan African locations, seeding deep mistrust among residents [15,22]. These examples violate generalized principles, such as equal cost-benefit sharing, self-determination, and inclusion, poisoning the collective efforts needed for wildlife protection [57]. More drastically, they reflect demands for decolonization and environmental justice, entailing a fair distribution of costs and benefits, participation in decision-making and the recognition of identities and cultures [17]. Traditional PA management may have low compliance with core design principles [59]. Linking the core design principles with local knowledge and values could benefit conservation work and just arrangements. However, like most PA assessments [2,49], our data reflect the perceptions of PA managers, which can involve certain biases and can differ from the perceptions of other stakeholders [10,60]. For a sound understanding of the role of these minimal core processes in

wildlife hunting, further knowledge synthesis across diverse gradients of actors, institutional arrangements, and societies is needed.

Overall, various manifestations, causes and effects of hunting on wildlife highlight the need for differentiation in research and PA risk assessments that list hunting as a single category [30] and the universal aspects emphasize the need to move beyond a Global South-North divide. Ultimately, in an increasingly human-dominated world, there might rarely be an ideal setting for wildlife, and future trajectories will likely worsen the identified wildlife-related challenges. Climate change and biodiversity loss will exacerbate the historical inequalities that currently drive unsustainable hunting associated with biodiversity and disease risks in the Global South [35]. They will further push people and wildlife into new areas, increasing HWCs along the regional axis, likely deepened by increasing demand for natural resources due to population and perpetual economic growth [27,61,62]. Current PA management, either control based or exclusionary, will be questioned by increasing demands for co-determination in the South, and returning predators in the North and growing predator populations will challenge human-wildlife relationships. Beneficial local circumstances had protective effects on wildlife but could not reverse the strong South-North effect. Consensus is growing that only systemic changes in economics, values, and society can prevent biodiversity loss and future pandemics and address long-standing inequalities [56,63]. These challenges entail social and normative negotiation processes on every single organizational level. Theory suggests that the minimal core principles briefly outlined above are applicable across contexts and scales and empower every relevant level to resolve within- and between-level conflicts, ultimately enabling more networked, just, and effective wildlife conservation [55,57].

Methods:

Data collection

Data was collected through face-to-face interviews with managers of 114 African and European PAs using a structured questionnaire [2,49]. The polygons of PAs were obtained from the World Database of Protected Areas [26]. The study was approved by the Ethics Council of the Max Planck Society and we obtained informed consent from all participants. We used three main criteria to sample the PAs from the World Database of Protected Areas [26]: 1) if possible, PAs in the category of national parks, 2) the availability of data to cross-validate our questionnaire data (Living Planet Database (LPD), IUCN SSC

A.P.E.S database reports (S22 Table), and 3) permission from governments to conduct surveys and the willingness of PA managers to fill in our questionnaire. We used face-to-face interviews to circumvent the challenge of acute data shortage on hunting and conservation strategies from an ecological, economic and social perspective [2]. As we were interested in the interface between biodiversity protection and resource use, we selected PA directors or their representatives as our interview partners, as we expected the highest data availability and expertise on hunting and its impacts on biodiversity from these key stakeholders [2]. We conducted the survey from December 2017 to September 2019 in 25 African and European countries and 114 parks (Fig. 1a, S1 Table). We used standardized questionnaires to collect data on socio-economic dimensions, community attributes, aspects of hunting and conservation interventions in place (see questionnaires in Supplementary Information). We further asked about changes in mammal and bird abundances over the past ten years (overall 462 species, 15 functional guilds/groups). Given that data of all occurring species were hardly available, the listed species are those where PA managers were able to report changes. Moreover, we collected grey literature in the form of reports and ecological and socio-economic surveys conducted by parks, management plans, and lists of confiscated animals and records of confirmed poaching cases (S20 Table). Even though we considered PA managers the best sources of this information, responses can deviate from reality due to unconscious and conscious biases. Thus, we cross-checked the validity of our data with data collated from the Living Planet Database (LPD) [64], IUCN SSC A.P.E.S database [65], published and unpublished reports, the Protected Area Management Effectiveness (PAME) assessments, and conducted an online survey with the same questionnaire with non-governmental organisations working at the surveyed PAs. For our abundance data, we found an overlap in 82.4% of the cases when comparing to other sources (S22 Table). When comparing questionnaires filled by NGOs and PA managers, we found an average overlap of 60.04% ± 12.27%. For the PAME assessment, we found an overlap of 54.8 % ± 13.3%. However, the power of the comparison was limited due to the low number of responses for the NGO survey and the PAME due to low number of comparable questions and different wording and time periods. Further details on this cross-validation are included in section 4 of the Supplementary information.

Models

We implemented Bayesian hierarchical regression models (BHRM) using the “brms” package (Bürkner, 2017). As priors, we used a standard normal distribution with a mean and standard deviation of 1. We made 2,000 iterations over four Markov chain Monte Carlo (MCMC) chains, but increased it due to failing in some models to 10,000 iterations [66]. In all models, we controlled for spatial autocorrelation

by including a Gaussian process over longitude and latitude for each PA [67,68] by using the function "gp" from the "brms" R package [66]. We standardized all variables from zero to one before compiling the indices. All predictor variables were transformed into a standard normal z distribution (with mean 0 and standard deviation 1) to facilitate comparison of the results from our models [69]. We have ruled out correlation and multicollinearity among predictors. Statistical analysis was conducted using R 3.6.1 [70]. The inspection of the MCMC results showed stationarity and convergence to a common target. All "Rhat" [71] values were below 1.01, and we had no divergent transitions after warmup. As we lacked prior information, we ran the models with weakly informative priors with a standard normal distribution (mean of 0 and standard deviation of 1). To achieve roughly symmetrical distributions and avoid influential cases, we square root- or log-transformed skewed predictors.

Responses:

To understand the differences and commonalities in hunting characteristics across the South-North gradient, we used seven binary response variables in nine models (Table 1).

i. Function of hunting: First, we investigated the multiple functions of hunting, defined as providing goods and services [10]. We grouped them based on their prevailing motivations into the three-dimensional structure common in sustainability science (e.g. Fischer 2013). An economic function combined hunting for subsistence and commercial interests. A social function comprised non-market-related hunting for entertainment and cultural and social necessities, and an ecological function included population control and killing due to human-wildlife-conflicts [10]. The response was the overall hunting index of all functions, and we tested the effect of the single categories based on an interaction between the hunting function and all main predictors (HDI, population density, community attributes).

ii. Manifestations of wildlife interactions: Second, we explored the prevalence of two common human-wildlife interactions. We used as a response the threat assessments of "hunting" and "killings because of human-wildlife conflicts". We included legal and illegal actions, but excluded ratings of legal hunting to explore whether this changed the results.

iii. Trophic level: Third, we assessed differences in hunting pressure between the ecological aspect of trophic levels of target animals. Our response was predator vs. non-predators as listed by

managers threatened by hunting. We defined small to large predatory mammals and birds of prey as predators, while primates, apes, insectivorous, omnivorous and herbivorous mammals and birds were considered as non-predatory.

iv. Threat location: Fourth, we aimed to understand the local effects on wildlife and its spatial location. Our first two responses were a rating of how protected species from the park were affected by illegal hunting within administrative park borders and outside the park borders. We included the components of our community characteristics individually, attitudes, trust, and local culture, and derived the AIC values from the differences between the models (ΔAIC) [72].

v. Follow-up analyses: We carried out follow-up analyses based on our results to further understand the scope of actions available to the park managers. We tested weather individual interventions of the "community conservation effort" index can foster supportive local community characteristics shown to protect wildlife inside and outside parks. The response was the index for “community characteristics”. Predictors were the index components of “community conservation effort”, namely "provision of economic benefits to the community", "implementation of livelihood projects", "scale of local inclusion", and "implementation of environmental awareness programs" separately in the model. In addition, the model contained population density, size, and country as control predictors, and used “continent” instead of HDI due to correlations.

All questions were recorded in an ordinal or Likert scale and transformed into binary responses, reducing the scope of error [73]. We controlled for odd numbers if a different break changed outcomes (i.e. grouping questionnaire answers moderate, high, very high, or respectively high or very high as high threat (S4-S8 Tables). For an overview of responses, see Table 1.

Predictors:

We approximated the socio-economic South-North gradient with the HDI (2017), a composite index of life expectancy, education, and per capita income (gross national income GNI (PPP) per capita) [74]. We included multiple-scale predictors known to affect human-wildlife relationships. At the landscape scale, we included human population density (2015) [2]. At the local scale, we constructed an index for community characteristics based on questions regarding nature-culture relationships, attitudes towards nature, conservation, the concept of protected areas and their current management, and levels of mutual

trust between communities and park management [53]. To account for conservation efforts, we generated one index for protection-based interventions that protect the resources within the park. The index included the presence of regular ranger patrols, buffer zones, and a permanent research station, and even if the primary intention is different, the presence of staff can have a similar protective effect as ranger patrols [75]. We further complied one index for community-based interventions, including conservation efforts altering the local context beyond park boundaries by community-based interventions (provision of benefits to communities, implementation of livelihood projects, awareness creation, inclusion of local communities). We controlled for park size [49] and included country as a random effect. All necessary random slope components were included. For the construction of the variables, see Tables 1, S2 and S3 Tables. We included all index components separately in the model to test the robustness of our indices.

Table 1 Overview of response variables and models

**Response**	**Predictors**
Hunting index of all functions (Index high, low)	HDI x functions of hunting (ecological, economic, social), human population density x functions of hunting, community characteristics x functions of hunting, CONTROL: PA size, country
Threat rating “hunting/poaching”, (legal, illegal).<br>(Threat: high, low)	HDI, human population density, community characteristics, protection-based interventions, community-based interventions, CONTROL: PA size, country, abundance mammals/birds
Threat rating “killing because of human-wildlife-conflict”, (legal, illegal).<br>(Threat: high, low)	HDI, human population density, community characteristics, protection-based interventions, community-based interventions, CONTROL: PA size, country, abundance mammals/birds
Trophic level of threatened species. Divided in predators, including raptors, predatory mammals, versus non-predatory, containing primates, apes, omnivorous, frugivorous, insectivorous mammals and birds.<br>(Threatened: yes, no)	HDI, human population density, community characteristics, protection-based interventions, community-based interventions, CONTROL: PA size, country, abundance predators
Rating of the threat to wildlife from parks through illegal hunting (killing/poaching/poisoning) within the administrative PA boundaries.<br>(Threat: high, low).	HDI, human population density, community characteristics, protection-based interventions, community-based interventions, CONTROL: PA size, country, abundance mammals/birds
Rating of the threat to wildlife from parks through illegal hunting (killing/poaching/poisoning) outside the administrative PA boundaries.<br>(Threat: high, low).	HDI, human population density, trust, protection-based interventions, community-based interventions, CONTROL: PA size, country, abundance mammals/birds

Rating of the threat to wildlife from parks through hunting (killing/poaching/poisoning) within the administrative PA boundaries, (Threat: high, low).	Replacement of community attributes by its index components: “trust”, “attitudes”, “culture”, HDI, human population density, protection-based interventions, community-based interventions, CONTROL: PA size, country, abundance mammals/birds
Rating of the threat to wildlife from parks through hunting (killing/poaching/poisoning) outside the administrative PA boundaries. (Threat: high, low).	Replacement of community attributes by its components: “trust”, “attitudes”, “culture”, HDI, human population density, protection-based interventions, community-based interventions, CONTROL: PA size, country, abundance mammals/birds
Community characteristics (beneficial characteristics: high, low)	Index-components of community-based interventions: provision of economic benefits to the community, implementation of livelihood projects, scale of local inclusion, implementation of environmental awareness programs, CONTROL: population density, country, continent

Literature

1. Roe D, Dickman A, Kock R, Milner-gulland EJ, Rihoy E, Sas-rolfes M. Beyond banning wildlife trade : COVID-19 , conservation and development. World Dev. 2020;136: 105121. doi:10.1016/j.worlddev.2020.105121
2. Laurance WF, Carolina Useche D, Rendeiro J, Kalka M, Bradshaw CJA, Sloan SP, et al. Averting biodiversity collapse in tropical forest protected areas. Nature. 2012;489: 290–293. doi:10.1038/nature11318
3. Benítez-López A, Alkemade R, Schipper AM, Ingram DJ, Verweij PA, Eikelboom JAJ, et al. The impact of hunting on tropical mammal and bird populations. Science (80-). 2017;356: 180–183. doi:10.1126/science.aaj1891
4. Nielsen MR, Meilby H, Smith-Hall C, Pouliot M, Treue T. The Importance of Wild Meat in the Global South. Ecol Econ. 2018;146: 696–705. doi:10.1016/j.ecolecon.2017.12.018
5. Trouwborst A, Krofel M, Linnell JDC. Recovery of large carnivores in Europe’s modern human-dominated landscapes. Biodivers Conserv. 2015;24: 1517–1519. doi:10.1016/S0169-5347(01)02290-X
6. Heurich M, Schultze-Naumburg J, Piacenza N, Magg N, Červený J, Engleder T, et al. Illegal hunting as a major driver of the source-sink dynamics of a reintroduced lynx population in Central Europe. Biol Conserv. 2018;224: 355–365. doi:10.1016/j.biocon.2018.05.011
7. Bischof R, Bonenfant C, Rivrud IM, Zedrosser A, Friebe A, Coulson T, et al. Regulated hunting re-shapes the life history of brown bears. Nat Ecol Evol. 2018;2: 116–123. doi:10.1038/s41559-017-0400-7
8. Cretois B, Linnell JDC, Moorter B Van, Kaczensky P, Nilsen EB. Coexistence of large mammals and humans is possible in Europe’s anthropogenic landscapes. 2020. doi:10.32942/osf.io/2md94
9. Lozano J, Olszańska A, Morales-Reyes Z, Castro AA, Malo AF, Moleón M, et al. Human-carnivore relations: A systematic review. Biol Conserv. 2019;237: 480–492. doi:10.1016/j.biocon.2019.07.002

10. Fischer A, Sandstrom C, Delibes-Mateos M, Arroyo B, Tadie D, Randall D, et al. On the multifunctionality of hunting an institutional analysis of eight cases from Europe and Africa. J Environ Plan Manag. 2013;56: 531–552. doi:10.1080/09640568.2012.689615
11. Tilker A, Abrams JF, Mohamed A, Nguyen A, Wong ST, Sollmann R, et al. Habitat degradation and indiscriminate hunting differentially impact faunal communities in the Southeast Asian tropical biodiversity hotspot. Commun Biol. 2019;2. doi:10.1038/s42003-019-0640-y
12. van Vliet N. “Bushmeat Crisis” and “Cultural Imperialism” in Wildlife Management? Taking Value Orientations Into Account for a More Sustainable and Culturally Acceptable Wildmeat Sector. Front Ecol Evol. 2018;6: 1–6. doi:10.3389/fevo.2018.00112
13. Oluwafemi MN. Globalization The Politics of Global Economic Relations and International Business. Carolina Academic Press- African World Series; 2012.
14. Plumptre AJ, Baisero D, Jędrzejewski W, Kühl H, Maisels F, Ray JC, et al. Are We Capturing Faunal Intactness? A Comparison of Intact Forest Landscapes and the “Last of the Wild in Each Ecoregion.” Front For Glob Chang. 2019;2. doi:10.3389/ffgc.2019.00024
15. Duffy R, Massé F, Smidt E, Marijnen E, Büscher B, Verweijen J, et al. Why we must question the militarisation of conservation. Biological Conservation. 2019. pp. 66–73. doi:10.1016/j.biocon.2019.01.013
16. Mohai P, Pellow D, Roberts JT. Environmental justice. Annu Rev Environ Resour. 2009;34: 405–430. doi:10.1146/annurev-environ-082508-094348
17. Martin A, Coolsaet B, Corbera E, Dawson NM, Fraser JA, Lehman I, et al. Justice and conservation: The need to incorporate recognition. Biol Conserv. 2016;197: 254–261. doi:10.1016/j.biocon.2016.03.021
18. Garland E. The Elephant in the Room: Confronting the Colonial Character of Wildlife Conservation in Africa. Afr Stud Rev. 2008;51: 51–74. doi:10.1353/arw.0.0095
19. Survival International. Decolonize Conservation. 2020 [cited 8 Oct 2020]. Available: https://www.survivalinternational.org/conservation
20. Jordan NR, Smith BP, Appleby RG, van Eeden LM, Webster HS. Addressing inequality and intolerance in human–wildlife coexistence. Conserv Biol. 2020;00: 1–8. doi:10.1111/cobi.13471
21. Ward K. Towards a relational comparative approach to the study of cities. Prog Hum Geogr. 2010;34: 471–487. doi:10.1177/0309132509350239
22. Marijnen E, Verweijen J. Selling green militarization: The discursive (re)production of militarized conservation in the Virunga National Park, Democratic Republic of the Congo. Geoforum. 2016;75: 274–285. doi:10.1016/j.geoforum.2016.08.003
23. Somerville K. Ivory: Power and Poaching in Africa. Oxford University Press; 2016.
24. Haase A, Bontje M, Couch C, Marcinczak S, Rink D, Rumpel P, et al. Factors driving the regrowth of European cities and the role of local and contextual impacts: A contrasting analysis of regrowing and shrinking cities. Cities. 2021;108: 102942. doi:10.1016/j.cities.2020.102942
25. Wolff M, Haase A. Viewpoint: Dealing with trade-offs in comparative urban studies. Cities. 2020;96: 102417. doi:10.1016/j.cities.2019.102417
26. Protected Planet. Protected planet. 2010 [cited 20 Jul 2020]. Available: https://www.protectedplanet.net/

27. König HJ, Kiffner C, Kramer-Schadt S, Fürst C, Keuling O, Ford AT. Human–wildlife coexistence in a changing world. Conserv Biol. 2020;00: 1–9. doi:10.1111/cobi.13513
28. Van De Walle J, Pigeon G, Zedrosser A, Swenson JE, Pelletier F. Hunting regulation favors slow life histories in a large carnivore. Nat Commun. 2018;9. doi:10.1038/s41467-018-03506-3
29. Doherty TS, Hays GC, Driscoll DA. Human disturbance causes widespread disruption of animal movement. Nat Ecol Evol. 2021. doi:10.1038/s41559-020-01380-1
30. Coad L, Leverington F, Knights K, Geldmann J, Eassom A, Kapos V, et al. Measuring impact of protected area management interventions: Current and future use of the global database of protected area management effectiveness. Philos Trans R Soc B Biol Sci. 2015;370. doi:10.1098/rstb.2014.0281
31. Andermann T, Faurby S, Turvey ST, Antonelli A, Silvestro D. The past and future human impact on mammalian diversity. Sci Adv. 2020;6: eabb2313. doi:10.1126/sciadv.abb2313
32. Ollos H, Society E wilderness. National Park Šumava counts on the wolf. 2019 p. https://wilderness-society.org/national-park-sumav.
33. van Beeck Calkoen STSSTS, Mühlbauer L, Andrén H, Apollonio M, Balčiauskas L, Belotti E, et al. Ungulate management in European national parks: Why a more integrated European policy is needed. J Environ Manage. 2020;260. doi:10.1016/j.jenvman.2020.110068
34. Morton O, Scheffers BR, Haugaasen T, Edwards DP. Impacts of wildlife trade on terrestrial biodiversity. Nat Ecol Evol. 2021; 1–9. doi:10.1038/s41559-021-01399-y
35. IPBES. IPBES Workshop on Biodiversity and Pandemics. 2020; 1–96.
36. Ripple WJ, Abernethy K, Betts MG, Chapron G, Dirzo R, Galetti M, et al. Bushmeat hunting and extinction risk to the world's mammals. Open Sci. 2016;3: 160498. doi:10.1098/rsos.160498
37. Ripple WJ, Newsome TM, Wolf C, Dirzo R, Everatt KT, Galetti M, et al. Collapse of the world's largest herbivores. Sci Adv. 2015;1: e1400103–e1400103. doi:10.1126/sciadv.1400103
38. Cowlishaw G, Mendelson S, Rowcliffe JM. Evidence for post-depletion sustainability in a mature bushmeat market. J Appl Ecol. 2005;42: 460–468. doi:10.1111/j.1365-2664.2005.01046.x
39. Manfredo MJ, Teel TL, Dietsch AM. Implications of human value shift and persistence for biodiversity conservation. Conserv Biol. 2016;30: 287–296. doi:10.1111/cobi.12619
40. Dietsch AM, Teel TL, Manfredo MJ. Social values and biodiversity conservation in a dynamic world. Conserv Biol. 2016;30: 1212–1221. doi:10.1111/cobi.12742
41. Coad L, Watson JEM, Geldmann J, Burgess ND, Leverington F, Hockings M, et al. Widespread shortfalls in protected area resourcing undermine efforts to conserve biodiversity. Front Ecol Environ. 2019;17: 259–264. doi:10.1002/fee.2042
42. Lindsey P a. Trophy hunting in Sub Saharan Africa: Economic scale and conservation significance. Best Pract Sustain Hunt. 2008; 41–47.
43. Croes BM, Funston PJ, Rasmussen G, Buij R, Saleh A, Tumenta PN, et al. The impact of trophy hunting on lions (Panthera leo) and other large carnivores in the Bénoué Complex, northern Cameroon. Biol Conserv. 2011;144: 3064–3072. doi:10.1016/j.biocon.2011.09.013
44. Davidson Z, Valeix M, Loveridge AJ, Madzikanda H, Macdonald DW. Socio-spatial behaviour of an African lion population following perturbation by sport hunting. Biol Conserv. 2011;144: 114–121.

doi:10.1016/j.biocon.2010.08.005
45. Müller J, Wölfl M, Wölfl S, Müller DWH, Hothorn T, Heurich M. Protected areas shape the spatial distribution of a European lynx population more than 20 years after reintroduction. Biol Conserv. 2014;177: 210–217. doi:10.1016/j.biocon.2014.07.007
46. Liberg O, Chapron G, Wabakken P, Pedersen HC, Hobbs NT, Sand H. Shoot, shovel and shut up: cryptic poaching slows restoration of a large carnivore in Europe. Proc R Soc B Biol Sci. 2012;279: 910–915. doi:10.1098/rspb.2011.1275
47. Herfindal I, Linnell JDC, Odden J, Nilsen EB, Andersen R. Prey density, environmental productivity and home-range size in the Eurasian lynx (Lynx lynx). J Zool. 2005;265: 63–71. doi:10.1017/S0952836904006053
48. Woodroffe R, Ginsberg JR. Edge effects and the extinction of populations inside protected areas. Science (80-). 1998;280: 2126–2128. doi:10.1126/science.280.5372.2126
49. Geldmann J, Manica A, Burgess ND, Coad L, Balmford A. A global-level assessment of the effectiveness of protected areas at resisting anthropogenic pressures. Proc Natl Acad Sci U S A. 2019; 1–7. doi:10.1073/pnas.1908221116
50. Kuijper DPJ, Churski M, Trouwborst A, Heurich M, Smit C, Kerley GIH, et al. Keep the wolf from the door: How to conserve wolves in Europe’s human-dominated landscapes? Biol Conserv. 2019;235: 102–111. doi:10.1016/j.biocon.2019.04.004
51. Wilkinson CE, McInturff A, Miller JRB, Yovovich V, Gaynor KM, Calhoun K, et al. An ecological framework for contextualizing carnivore-livestock conflict. Conserv Biol. 2020;In press: 1–14. doi:10.1111/cobi.13469
52. Young JC, Searle K, Butler A, Simmons P, Watt AD, Jordan A. The role of trust in the resolution of conservation conflicts. Biol Conserv. 2016;195: 196–202. doi:10.1016/j.biocon.2015.12.030
53. Brooks JS, Waylen K a, Borgerhoff Mulder M. How national context, project design, and local community characteristics influence success in community-based conservation projects. Proc Natl Acad Sci U S A. 2012;109: 21265–70. doi:10.1073/pnas.1207141110
54. Højberg PL, Nielsen MR, Jacobsen JB. Fear, economic consequences, hunting competition, and distrust of authorities determine preferences for illegal lethal actions against gray wolves (Canis lupus): a choice experiment among landowners in Jutland, Denmark. Crime, Law Soc Chang. 2017;67: 461–480. doi:10.1007/s10611-016-9670-2
55. Ostrom E. Governing the Commons: the evolutions of institutions for collective action. Political economy of institutions and decisions. 1990. Available: http://www.mendeley.com/catalog/governing-commons-evolutions-institutions-collective-action/
56. IPBES. Summary for policymakers of the global assessment report on biodiversity and ecosystem services of the Intergovernmental Science-Policy Platform on Biodiversity and Ecosystem Services. S. Díaz, J. Settele, E. S. Brondízio E.S., H. T. Ngo, M. Guèze, J. Aga. IPBES secretariat, Bonn, Germany.; 2019. Available: https://ipbes.net/system/tdf/ipbes_global_assessment_report_summary_for_policymakers.pdf?file=1&type=node&id=35329

57. Wilson DS, Ostrom E, Cox ME. Generalizing the core design principles for the efficacy of groups. J Econ Behav Organ. 2013;90. doi:10.1016/j.jebo.2012.12.010
58. Baggio JA, Barnett AJ, Perez-Ibara I, Brady U, Ratajczyk E, Rollins N, et al. Explaining success and failure in the commons: The configural nature of Ostrom's institutional design principles. Int J Commons. 2016;10: 417–439. doi:10.18352/ijc.634
59. Tebet G, Trimble M, Pereira Medeiros R. Using Ostrom's principles to assess institutional dynamics of conservation: Lessons from a marine protected area in Brazil. Mar Policy. 2018;88: 174–181. doi:10.1016/j.marpol.2017.10.037
60. Ghoddousi A, Bleyhl B, Sichau C, Ashayeri D, Moghadas P, Sepahvand P, et al. Mapping connectivity and conflict risk to identify safe corridors for the Persian leopard. Landsc Ecol. 2020;35: 1809–1825. doi:10.1007/s10980-020-01062-0
61. Puaschunder J. Governance & climate justice: Global south & developing nations. Governance & Climate Justice: Global South & Developing Nations. Palgrave Macmillan; 2020. doi:10.1007/978-3-319-63281-0
62. Beyer RM, Manica A, Mora C. Shifts in global bat diversity suggest a possible role of climate change in the emergence of SARS-CoV-1 and SARS-CoV-2. Sci Total Environ. 2021;767: 145413. doi:10.1016/j.scitotenv.2021.145413
63. IPCC. Summary for Policymakers SPM. Glob Warm 15°C An IPCC Spec Rep impacts Glob Warm 15°C above pre-industrial levels Relat Glob Greenh gas Emiss pathways, Context Strength Glob response to Threat Clim Chang. 2018; 32. Available: https://www.ipcc.ch/site/assets/uploads/sites/2/2019/05/SR15_SPM_version_report_LR.pdf
64. Living Planet Database (LPD). No Title. In: Zoological Society of London [Internet]. 2018. Available: https://livingplanetindex.org/home/index
65. Kühl HS, Williamson L, Sanz C, Morgan D, Boesch C. A.P.E.S., IUCN SSC A.P.E.S. database. 2007. Available: http://apesportal.eva.mpg.de
66. Bürkner PC. brms: An R package for Bayesian multilevel models using Stan. J Stat Softw. 2017;80. doi:10.18637/jss.v080.i01
67. Schielzeth H, Forstmeier W. Conclusions beyond support: Overconfident estimates in mixed models. Behav Ecol. 2009;20: 416–420. doi:10.1093/beheco/arn145
68. McElreath R. Statistical Rethinking. Stat Rethink. 2020. doi:10.1201/9780429029608
69. Schielzeth H. Simple means to improve the interpretability of regression coefficients. Methods Ecol Evol. 2010;1: 103–113. doi:10.1111/j.2041-210X.2010.00012.x
70. R Core Team. R: A language and environment for statistical computing (R Foundation for Statistical Computing). Vienna, Austria: R Core Team; 2017. Available: https://www.r-project.org
71. Gelman A, Carlin JB, Stern HS, Dunson DB, Vehtari A, Rubin DB. Bayesian data analysis, third edition. Bayesian Data Analysis, Third Edition. 2013.
72. Burnham KKP, Anderson DRD. Model Selection and Multimodel Inference: A Practical Information-Theoretic Approach (2nd ed). Ecological Modelling. 2002. doi:10.1016/j.ecolmodel.2003.11.004
73. Lindsey PA, Petracca LS, Funston PJ, Bauer H, Dickman A, Everatt K, et al. The performance of African protected areas for lions and their prey. Biol Conserv. 2017;209: 137–149.

doi:10.1016/j.biocon.2017.01.011
74. UNDP. United Nations Development Programme. 2020 [cited 24 Nov 2020]. Available: http://hdr.undp.org/en/content/human-development-index-hdi
75. N'Goran PK, Boesch C, Mundry R, N'Goran EK, Herbinger I, Yapi FA, et al. Hunting, law enforcement, and African primate conservation. Conserv Biol. 2012;26: 565–71. doi:10.1111/j.1523-1739.2012.01821.x

Data availability

The datasets generated and analyzed during the current study are not publicly available due to sensitivity and confidentiality of information but are available from the corresponding author on reasonable request.

Code availability

The R code used for data analyses in this study is available as supplementary information.

Figure 1

Click here to access/download;Figure;Fig1.tif

Fig1.tif

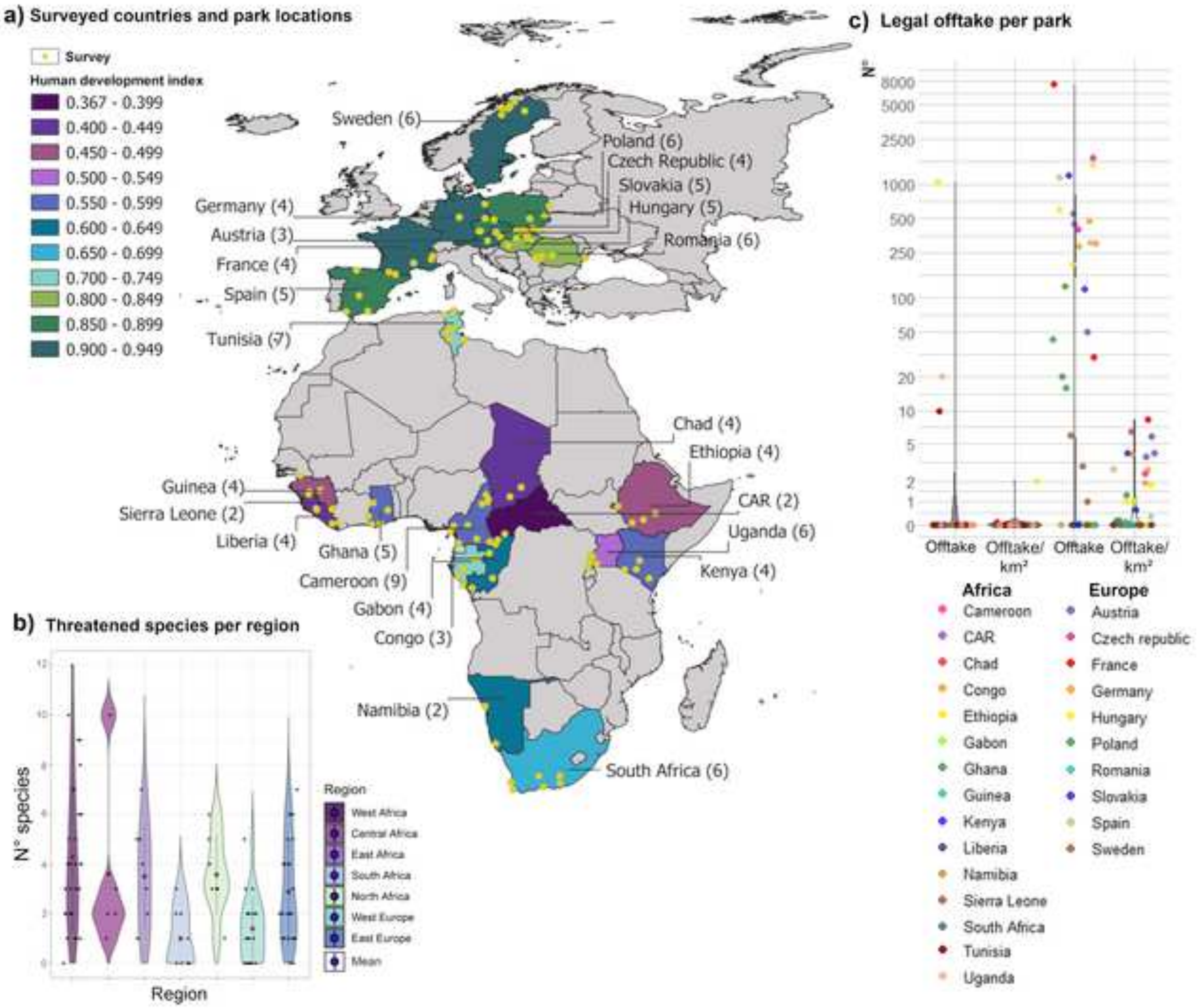

Figure 2

Click here to access/download;Figure;Fig2.tif

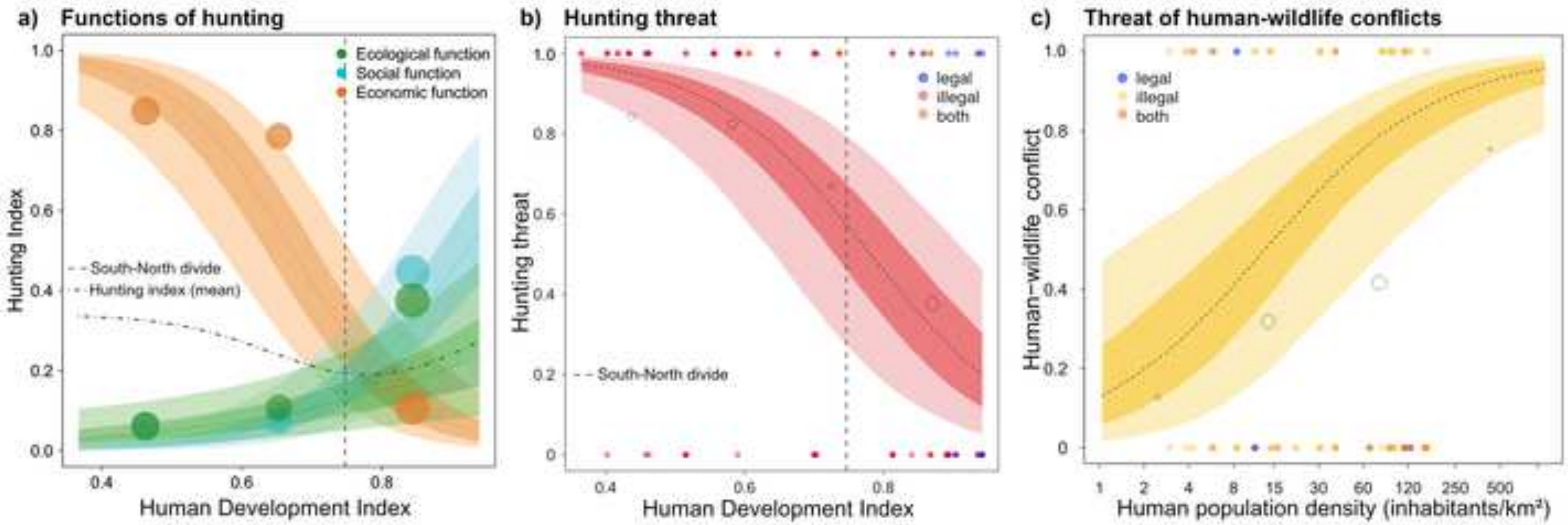

Fig2.tif

Figure 3

Click here to access/download;Figure;Fig3.tif

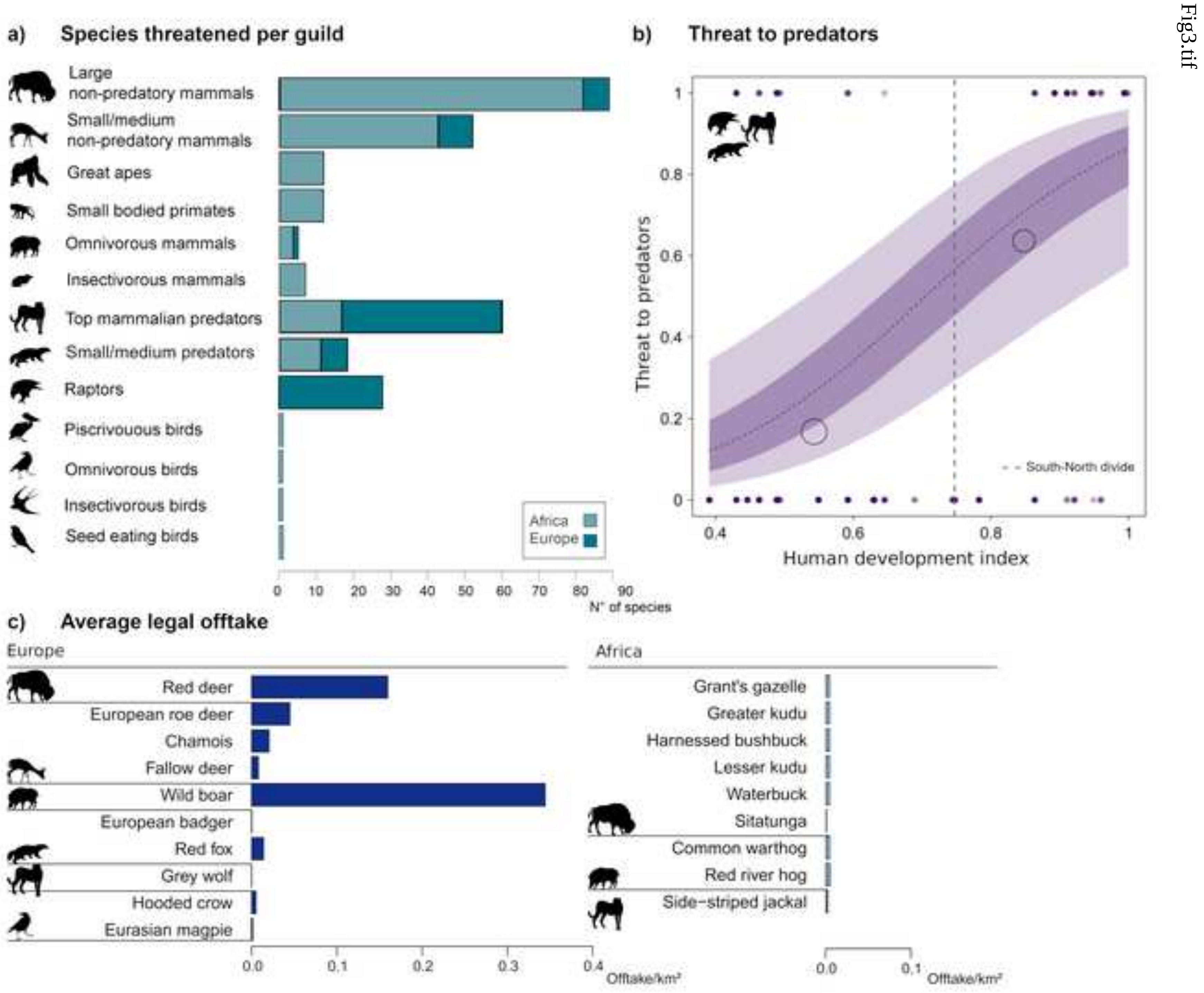

Figure 4 Click here to access/download;Figure;Fig4.tif

Fig4.tif

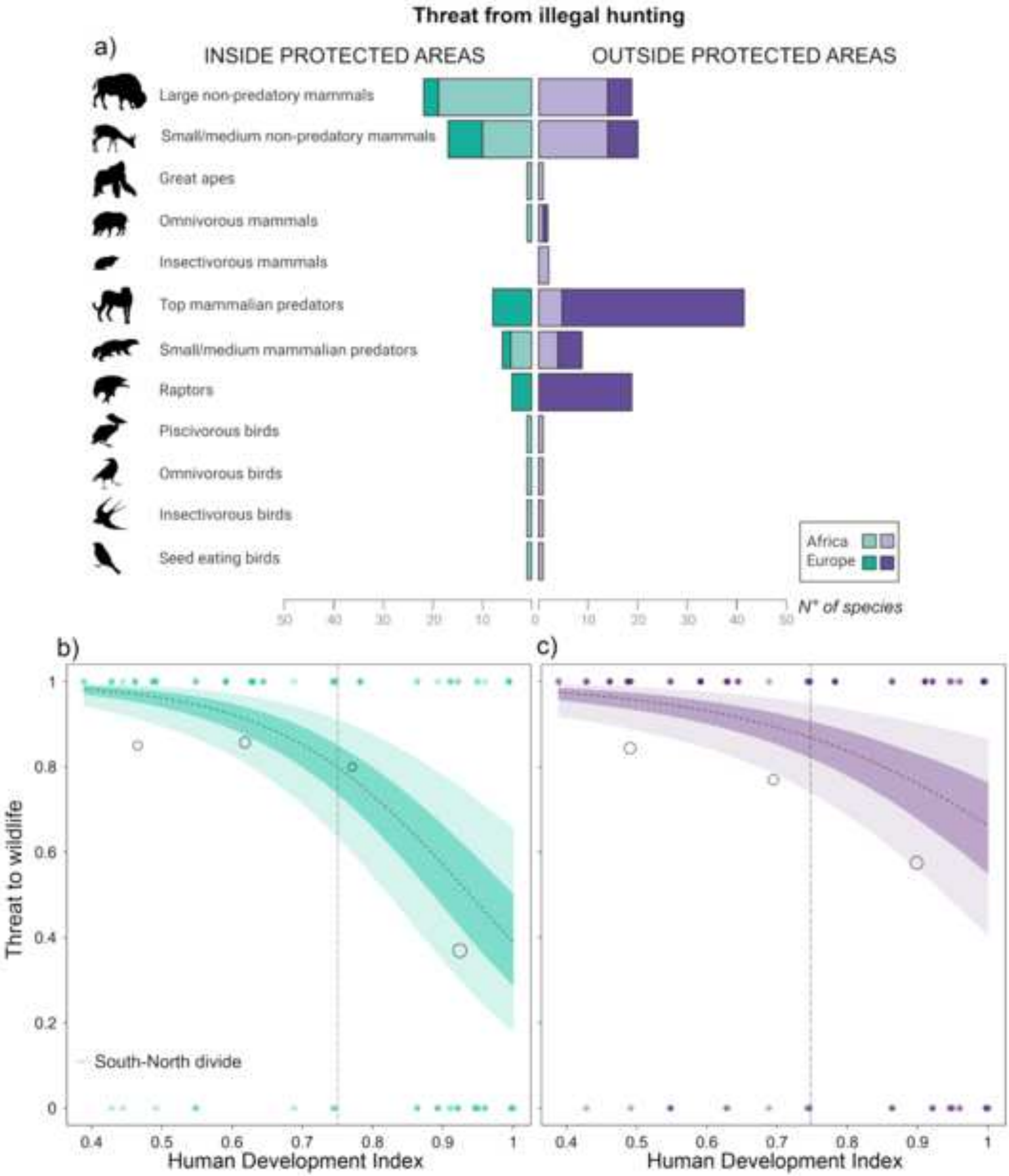

Figure 5

Click here to access/download;Figure;Fig5.tif

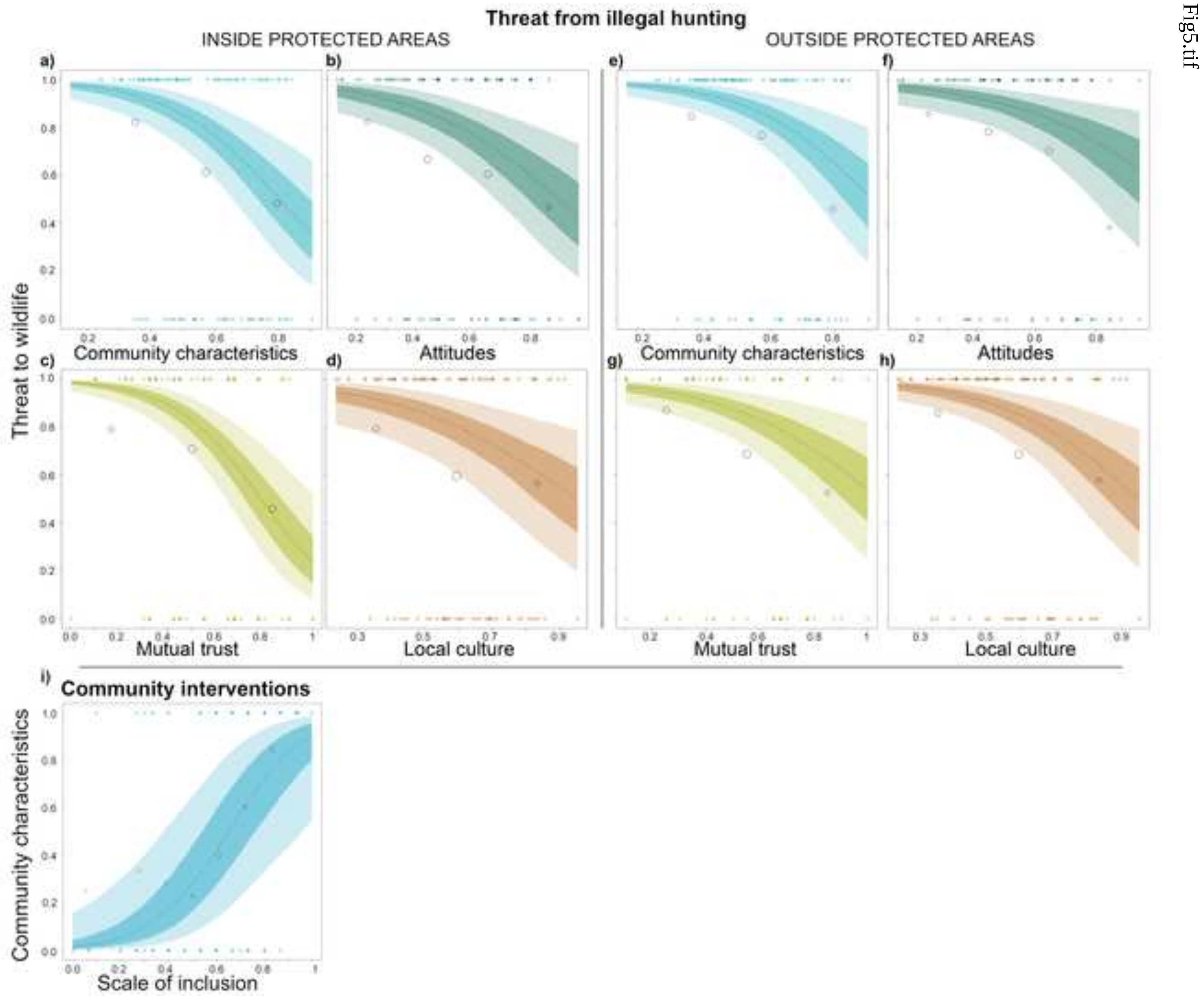

Click here to access/download
Supporting Information
Appendix_PB_17_06_21.docx

Title Page

Click here to access/download
Other
Title page_25.05.2021_PB.docx
