# Supplementary material for: Analysis of differences and commonalities in wildlife hunting across the Africa-Europe South-North gradient
Source: PLoS Biol. 2022 Aug 30;20(8):e3001707. doi: 10.1371/journal.pbio.3001707 (PMC9426919; doi:10.1371/journal.pbio.3001707)
Supplement: Attachment — Submitted filename: Review_29_4_2022.docx [file pbio.3001707.s019.docx]

GENERAL COMMENTS

Comment reviewer:

This is an enormous study, conducting face-to-face interviews with park managers across 114 protected areas in 25 countries in Africa and Europe. The sheer breadth and volume of data make this a landmark study. I buy the main thesis of the paper, that unsustainable exploitation of wildlife is often seen as a problem peculiar to the global south, hampering identification of similarities and differences in patterns that can tell us something about the underlying processes.

**Reply:** We would like to thank the reviewer for taking the time and effort to carefully read the manuscript and for providing a very constructive review with very helpful suggestions and comments. We follow your suggestions and have incorporated your comments in the revised manuscript.
_______

Comment reviewer:

The main body of the paper is brilliantly written, with great figures and text that concisely and clearly tell the unfolding story of the analyses. The results are compelling, and wonderfully unify hunting as a phenomenon that has interesting commonalities and interesting differences across a very broad gradient of socio-economic contexts. The discussion, however. goes quite some distance beyond the data, but by and large it feels OK. The one thing that does seem unfortunately missing from the discussion is the voices of the park managers themselves. Were there any open-ended questions in the survey that could be used to liven up the discussion with some thoughts from the front line?

**Reply:** Thank you very much for these two comments. With regard to the discussion, we have now changed considerably the last section ‘Generalizable principles, universal pathways’. We renamed the section to “Potential regional and universal solutions”’ to focus more on the findings of our study and develop more concrete implications derived from the differences and commonalities found in this study. With regard to the voices of park managers, there were already some statements in the previous manuscript version; however, they were not easily recognizable as such. We present them now as ‘pers. comm’ to highlight them as the voices of park managers. We have also added new quotes, which makes indeed the paper more applicable because it enriches the purely quantitative data with real experiences from practice.
_______

Comment reviewer:
[the questionnaire itself was not included in the version of the supplementary information that I could access, which was unfortunate]

**Reply:** We have now included the questionnaire as an item in supplementary material.
_______
However, my main concern is focused more "under the hood". Most notably, the way in which variables were constructed is unclear, hampering detailed interpretation of the results, and rendering the study unreproducible. For example, page 17 "Responses" is very confusing, with 7 binary response variables, 9 models, 5 bullet points, and only vague explanations of the response variables in each. For all of the response variables it is not clear exactly how they are calculated, or how they are binary as specified in the preamble to this section. This section needs to make clear to the reader precisely how the data from the questionnaire are processed to form the response variables. Many of the variables were derived from Likert scales, but how were data from multiple scales combined, and how were cutoffs for binary divisions determined etc. There do not appear to be any sensitivity analyses presented that explore different ways of deriving the variables. Even wading through TableS2 doesn't provide enough information for the reader to work out exactly how the responses in the questionnaire were used to calculate variable values. The same is true for the predictor variables, and much more clarity is needed around describing how all the variables used in the study were put together.

**Reply:** Thank you very much for pointing out this lack of information and clarity. We have now considerably improved Table S2 and Table 1 to give more detailed information on the construction of response and predictor variables, including an explanation of how we derived the variables from Likert scales and incorporated them into the analyses. Moreover, we explained more the results performed with using different thresholds (Table S2, S3-S9). In addition, we have again emphasized that all analyses were performed with different thresholds.

_______

SPECIFIC COMMENTS

Comment reviewer:
Page 4, para 1
Paragraphs starts well, but then goes rather hyperbolic, e.g. "synthesis along a diverse gradient may make it possible to identify deeper underlying mechanisms that outline generalizable pathways within the exceedingly complex phenomenon of wildlife overexploitation" - consider simplifying the writing in this paragraph to make it more direct and meaningful.

**Reply:** Thank you for pointing this out. We rewrote this sentence to make it more readable. It now reads on page 4 as:

“Based on this extended evolutionary perspective, a large-scale, comparative analysis of hunting patterns across socioeconomic contexts will help us to better understand the mechanisms that outline generalisable pathways and solutions within the complex phenomenon of wildlife overexploitation.”
_______

Comment reviewer:
Page 9
Important to caveat many of these analysis as being correlational. They are not robust tests, e.g. of effects of protection efforts on threat. A robust test would require before / after, or BACI designs. I think the analyses are valuable, but there just needs to clear recognition that these are associative results only, and do not indicate effects of predictor variables on response variables. In addition, there could be feedback effects, e.g. more ranger patrols being instituted when hunting levels are high.

**Reply:** Thank you for this comment and bringing up this issue. We entirely agree and have added an extra paragraph in the discussion to explain this limitation of the study. See Limitations of the study.

We also changed wording as far as possible throughout the manuscript and use formulations such as ‘being associated’ and ‘being related’ to avoid the impression that our study is based on causal inference.
_______

Comment reviewer:
Page 16, para 1

Unclear sentence: "For our abundance data, we found an overlap in 82.4% of the cases when comparing to other sources (S22 Table)". Unclear how overlap is judged. I appreciate S22 and section 4 of SI is being referred to, but this point should be made much clearer in the main text otherwise that prime real estate is being wasted. The very next sentence "When comparing questionnaires filled by NGOs and PA managers..." suffers from exactly the same problem.

**Reply:**
Thank you or this valuable point. We wrote now on page 23:

“Thus, we cross-checked the validity of our data with data collated from the Living Planet Database (LPD) [69], IUCN SSC A.P.E.S Database [70], published and unpublished reports, and Protected Area Management Effectiveness (PAME) [71] assessments. Furthermore, we conducted an online survey with the same questionnaire with nongovernmental organisations (NGOs) working in the surveyed PAs. We grouped similar variables as in our analyses into two levels (declined or stable/improved and low, high) and compared the values between our data and these reference sources. For our abundance data, we found an overlap regarding increasing, stable, or decreasing population trends in 82.4% of the cases compared to other sources (S23 Table). When comparing questionnaires completed by NGOs and PA managers, we found an average overlap of 60.04% ± 12.27%. For the PAME assessment, we found an overlap of 54.8% ± 13.3%.”

_______

Reviewer #2:
[please also see attached file]

Dear authors, I commend you for a comprehensive and well-executed study comparing wildlife hunting inside and outside PA's across 2 continents. The authors find commonalities and differences in the threat posed by hunting to wildlife and explore a range of predicted variables in an effort to understand the social, governance and economic aspects underpinning the patterns.

**Reply:** Thank you very much for your positive assessment of our study and your very constructive review. We greatly appreciate the time and effort you have put into this. We have tried our best to incorporate all of your very helpful suggestions and comments and give a detailed point-by-point response below.
______

Comment reviewer:

Please find detailed comments in the attached documents, but here are some major comments:

Methods are suitable for the questions asked, but I would advise the authors to develop the choice of predictors so it aligns with the scope of the work; use literature to support the selection of predictors at various scales, and how do they relate directly to the questions posed here. It would also help if hypotheses are postulated based on these arguments.

**Reply:** Thank you for this comment to which we entirely agree. We have now inserted an additional column into Table S2 ‘Hypotheses and references’, in which we present specific hypotheses to support inclusion of predictors along with relevant literature. In addition, we have formulated hypotheses for each question in the introduction.

Page 5/6

“To understand 1) why, we examined a) the predominant function of hunting, using "economic" (subsistence, commercial hunting), "sociocultural" (nonmarket cultural, social, recreational aspects), and "ecological" (population management, killing due to HWC) motivations (analysis i, Table 1) [9]. We hypothesised that a change in the motivation for hunting across the studied PAs would reflect changing socioeconomic conditions (all hypotheses in S2 Table). We expected a strong economic function of hunting in areas with low HDI values due to a reliance on hunting for livelihoods [3] that is gradually replaced by other functions with an increasing HDI. We also assessed the impact of hunting as a human–wildlife interaction and, more specifically, in response to HWC (analysis ii). We expected a decreasing threat of hunting along the S-N gradient since increasing prosperity reduces hunting pressure related to maintaining livelihoods [3]. In contrast, we expected the effects of HWC to be higher in the Global South due to the higher proportionate costs to households from HWC [17]…..”

_______

Comment reviewer:

"Gradient": this was a major sticking point: I understand that he authors wan to sell this manuscript to a high IF journal, and that the "Global N-S gradient" has a certain allure to it, but this is comparison of European and African wildlife hunting in 25 PAs, no a comprehensive global analysis that warrants this name. In fact, the Results compare Europe and Africa, and the Discussion build on these comparison, and I can't see the 'gradient' anywhere. Also, the Introduction does not make ha case hat a gradient exists, but that comparative analyses of wildlife management systems in different continents can lead to improved inference compared to focusing on a single continent. It seems like the authors use it as a catchphrase to increase the appeal. If so, I strongly advise the authors to reframe the paper and be truthful to what this paper presents: a comparative study of hunting in 25 European and African PAs. This would not affect the outcomes or importance of this work.

**Reply:** We thank the reviewer for this comment. We would like to point out that our study included 114 PAs in 25 countries, and not only 25 PAs as mentioned above. This is a much larger sample size covering a diverse range of settings. Although we agree that our study is not global, the inclusion of two continents and 25 countries were in fact was intended to cover important socio-economic gradients. We have therefore made it clearer throughout the manuscript and response letter that our sample size is 110 parks in 25 countries. However, if there are other arguments besides the wrongly assumed sample size that speak against our approach, we are of course open to a further discussion.

Nevertheless, we see this as a very important objection and incentive to explain better why we have chosen this approach. Furthermore, we followed your suggestion to tone down some of the wording to situate the study in its context.

1) Why the concept of the Global South-North gradient concept:

Even though a comparison between Europe and Africa can be an exciting starting point, we deliberately have chosen the term gradient to go beyond the often-used binary distinction of Global South/North, Africa-Europe. Neither Africa nor Europe are homogeneous continents, neither from a historical, political, ecological or socio-economic nor from a park management point of view. We have chosen Africa and Europe, as it is the steepest socio-economic gradient worldwide, which is still highly nuanced. Nevertheless, the gradient still presents typical features of the North-South gradient applicable to other regions such as South America or Asia, such as tropical and subtropical climate, flora and fauna, socio-economic disparities, colonial history, and partial continuities in the conservation sector. We also believe that a purely binary analysis will not yield much new here, since it does not reveal the gradual changes. As we consider the reviewers' comment very important, we have elaborated more clearly in the manuscript why we chose Africa and Europe as examples to better understand differences and commonalities across the Global South and North. We hope that we have been able to explain our approach better and to your satisfaction.

To explain better why we chose the S-N gradient concept, we added now:

Page 4

“The Global South-North gradient describes a specific socio-economic gradient that has been shaped by the recent historical division into largely colonised and colonial states. Even today, one-fourth of the world's population in the Global North controls four-fifths of global income [12]. The state of biodiversity appears in different stages, as more wildlife species have been exterminated in the Global North [4,13].”

Page 23

“We surveyed the southern, eastern, western, central, and northern parts of Europe and Africa to capture a steep yet nuanced gradient regarding socioeconomic and PA management conditions along the Global South-North axis. This gradient ranges from countries in Central Africa with extremely low HDI values to the high HDI-scored countries of Northern Europe but also countries in Southern and Northern Africa that reach values similar to those in Eastern Europe (Fig. 1a)”

2) Why we consider our survey covering a gradient:

Since it was difficult to recognize a gradient, we added now in addition to the map, showing the different HDI values of the countries studied (Fig. 1a), a new figure to the manuscript showing the HDI values of the PAs we studied (Fig. 1d). The figure 1d shows well the HDI gradient covered by our survey. It is important to note that some values in Africa are closer to European values and vice versa compared to within continent variability. We have also plotted the other variables from our models (additional figure: Appendix Fig. 1, Fig. 2), which do not show this pattern but rather a more uniform distribution.

3) We toned down some of our statements to prevent the impression of a global study and point out that we chose here Africa and Europe as one exemplary S-N gradient (we have adopted your S-N notation for the manuscript).

We changed therefore the title from:

**Differences and commonalities in wildlife hunting across the Global South-North gradient**

To:

**Differences and commonalities in wildlife hunting across a Global South-North gradient**

We further added to the text:

Page 1:

“Herein, we examined differences and commonalities in hunting characteristics across an exemplary South-North gradient approximated by the Human Development Index (HDI) using face-to-face interviews with 114 protected area (PA) managers in 25 African and European countries.”

Page 4

“Herein, we aim to understand the differences and commonalities of wildlife hunting across the contextual South-North gradient using Africa and Europe as examples.”

Page 5

“ We used the Human Development Index (HDI) to approximate the different socioeconomic contexts and path dependencies across the two continents (Fig. S1), which we refer to as a South-North gradient (S-N gradient) (Fig. 1d).”

To avoid the impression that this is a global study, we have changed the wording throughout the manuscript from Global South-North gradient to South-North gradient, or respectively S-N gradient.

_______

Comment reviewer:

The Discussions make a lot of unsupported or contradictory statements, which are only made worse by the use of words such as "remarkable" (seemingly to also boost the importance of this work for a high IF journal). No need for that; this is a solid study without the need to use hyperbolas. I made many comments in the Discussion in which I am trying to challenge the authors to delve deeper in some portions of the wildlife literature and move away from grandiose but unsupported statements. In particular, the Discussion needs a much stronger presence of the actual results and how they relate to the assertions. Right now it reads largely disconnected from Results.

**Reply:** We thank the reviewer for these comments. We provide detailed statements to each point made below. We have now rewritten the paragraph of the discussion ‘Generalizable principles, universal pathways’ and focus more on discussing the actual results of our study. We renamed the paragraph: “Potential regional and universal solutions” (Page 18). We have deleted words such as ‘remarkable’ from the text so as not to exaggerate our results.

_______

Comment reviewer:

Lastly, the last section should contain less arm-waving and theory and more practical implications of this important work. What exactly do the authors propose as immediate steps to safeguard wildlife based on this analysis? Talking about SES and frameworks, and how everyone should work together is great, but not very realistic or useful in the near-term, which is what is needed to stop defaunation. I challenge the authors o think outside the box and find practical solutions to met this challenges based on their work.

In a nutshell, this is a great paper that needs some additional explanations and stepping back from big statements to more practical issues. Thank you for the opportunity to read your work.

**Reply:** Thank you very much for your positive assessment of our study and the suggestion on how to improve the manuscript. We re-wrote the last section more concrete recommendations on how to reduce defaunation in the short term and present. See page 19 section Potential regional and general solutions.

Comments in the text:

_______

Page 3

“Scientific activity and international scrutiny commonly focus on the Global South [1], where poverty-driven and illegal hunting leads to dramatic declines in mammal and bird populations [2–4]. In the more affluent nations of the Global North, returning and growing populations of large herbivores and predators such as grey wolves (Canis lupus) and European bison (Bison bonasus) are symbols of conservation success [5].”

Comment reviewer:

The Global N and S concept is a bit misleading here. First, although catchy, these are vague terms. Please state your focus on European and African wildlife management systems. What is the "gradient" that authors make the case for? It seems like the entire paper is a comparison of hunting in Europe and Africa.

**Reply:** Thank you for your comments and perspective on the Global N and S concept. See also our response to your related comment above. Our study included 114 PAs in 25 European and African countries with the largest possible coverage of conditions along variable space. Although we fully agree that our focus on African and European PAs is only part of the overall Global S/N, we still think that mentioning this concept is justified, by highlighting at the same time the limitations of our study. We have therefore decided to highlight more the focus of our study on African and European PAs and present it as one example of the Global S/N differences/similarities (see examples mentioned above).

E.g., page 4:

“Herein, we aim to understand the differences and commonalities of wildlife hunting across the contextual South-North gradient using Africa and Europe as examples.”

Page 21/22

“Moreover, this study examines only one exemplary gradient between two continents, Africa and Europe. Therefore, more research including other continents is needed to understand the gradual changes and commonalities across the South-North axis.”

_______

Page 3

“Despite these similarities, most research remains geographically “segmented” in a firmly anchored North-South binary [2,3,9], and little is known about how hunting unfolds across the Global South-North gradient. “

Comment reviewer:

This is vague; I believe that the authors make the case that developed and developing countries have been treated separately as case studies, and this work reaches across both. Again, this is not a gradient, but a comparison of Europe and Africa. By telling what this study really focuses on, does not detract from its novelty or usefulness.

Overall, I recommend that the authors move away from seemingly catchy concept of Global N-S gradient, and reword the Intro to highlight the comparison implemented here.

**Reply:** We thank you for your comment and perspective on this topic. As this comment is also related to some of the comments above, please see also our previous responses. We would like to highlight that conditions in African PAs are very different and heterogeneous, as they are in European PAs. The variation of conditions within each continent is very large and some PAs are closer in their conditions to PAs on the other continent than on the same. We agree that the mentioning of the Global S-N gradient without the limiting focus on European and African PAs may give a wrong impression of the study. We have therefore decided to tone down the Global S-N gradient wording. We still think, however, that its use is justified when presenting our study as one example of the Global S-N concept, and better highlight all the limitations.

_______

Page 4

“By contrast, comparisons across contrasting environments further our understandings

Of the differences, commonalities and universal mechanisms within a phenomenon and help to reduce the complexity through generalization against the comparative horizon of the contextual scale [24,25].”

Comment reviewer:

yep, this is a comparison, not a 'gradient'

**Reply:** Thank you for your comment and we would like to provide some clarifications. We have designed the study to compare hunting against a contextual gradient. We did this to get more nuanced results, which can tell us something about the universal mechanisms. These insights might provide knowledge for various settings beyond Africa and Europe. A binary comparison would also have been a very interesting approach but would probably require a slightly different analysis. For example, instead of including HDI, we would have needed to include the binary variable Africa/Europe. However, we have decided against this for the reasons mentioned above. We also think that a binary analyses would have revealed little new insights. We hope that we have been able to explain this better in the revised version of the manuscript.

_______

Page 4/5

“We focus on protected areas (PAs) and adjacent areas as an interface of biodiversity protection and resource use.”

Comment reviewer:

Please be more specific. What are "adjacent areas"? How far away from PA's are you considering hunting impacts? Are they proportional to PA size?

**Reply:** This is indeed an important point. Adjacent areas in our study are not defined on a metric scale. The park managers ask whether species from the park are more endangered by illegal hunting inside the park borders or when they roam outside the park borders. How far this zone extends depends on the species and, like you rightly mentioned, on the park's size (therefore, park size was included as a control variable in all analyses). The other aspect refers to the municipalities adjacent to the park. This can also be very park-specific. Considering the variations, we considered the ‘adjacent areas’ as a descriptive measure without defining a fixed size and according to the interviewee’s perceptions.

To clarify this issue we added now:

On page 10:

“How far species range beyond borders is dependent on the ecology.”

On page 11:

Threats to animals when ranging outside PAs also declined, albeit to a lesser extent (Fig. 4c, S12 Table, HDI: estimate = -0.95, CI = [-1.94, -0.01].

On page 25:

“Our first two responses were a rating of how protected species from the park were affected by illegal hunting within administrative park borders or when ranging outside the park borders.”

On page 26:

“Which municipalities and in which area belong to the local communities was determined here by the park manager.”

_______

Page 5

“PAs allow hunting for wildlife management, and it is usually subject to regulations designed to guarantee sustainability.”

Comment reviewer:

please develop... in most cases considered here, legal hunting for wildlife management occurs outside PAs as well. Thus, comparisons of "inside vs outside PAs" may be blurred in many cases

**Reply:** That is correct. In our study, we therefore only compared the threat from illegal hunting inside and outside parks, whereby illegal hunting outside parks mainly refers to protected species. We have now made this clearer by pointing out throughout the manuscript that this refers to illegal hunting.

E.g., page 6

“To understand 3) where, we examined illegal hunting and whether this threat to wildlife tends to be located more within or outside PA boundaries (analysis iv).”

_______

Page 5

“To answer 2) “what”, we compiled the targeted species and examined whether ecological parameters such as the trophic level influence the likelihood of being affected by hunting. To understand 3) “where”, we examined illegal hunting and whether this threat to wildlife tends

to be located more within or outside administrative PA boundaries.”

Comment reviewer:

you need to develop this a bit here; "target species" comes out of nowhere.. target for what? your assessment? hunting? HWC?

**Reply:**

That is a good point. We wrote now on page 6:

**“**To answer 2) what, we compiled information about species targeted by legal and illegal hunting (including HWCs) and examined whether ecological parameters such as trophic level influence the likelihood of being threatened by illegal hunting (analysis iii).**”**

Further, we defined hunting on page 5 as:

**“**Therefore, we define hunting as the entirety of activities involved in the management and pursuit of wildlife [10].**”**

_______

Page 6

“The threat from hunting was not confined to the Global South.”

Comment reviewer:

This needs more development of hypotheses in the Intro... Why would threats be confined to Globals S. There are plenty of examples in Europe where wildlife management via hunting is subpar and not rooted in species biology and biology, for example in several Eastern European countries.

Moreover, the only "gradient" that I see in the Results is the human development index. Yet, the results are still heavily focused on comparing European and African wildlife hunting.

**Reply:** Thank you for your comment and we fully agree that threats are not confined to the Global S. The point we want to make here is related to the general perception of threats and perceived differences in Africa and Europe due to double standards. Frequently, hunting in the Global South is considered to be ‘unnecessary’, while in Europe it is considered ‘necessary’ due to absence of large predators or human-wildlife conflict. And we agree with your point that the gradient refers to HDI. We created an additional plot to Fig.1 (1e), showing that HDI variation within continents is larger than the gap between Africa and Europe. Given the spread of conditions in Africa and Europe, we think it would not be appropriate to talk about a comparison between continents here. It is more appropriate to talk about a comparison with regard to the different context PAs are embedded in. We highlighted now by added a new sentence that in some cases of the results, there is a clear divide between Africa and Europe, meanwhile others change gradually over the gradient. We were especially interested in this nuanced outcome.

e.g., page 14

“While some differences, such as the threat of hunting, varied gradually across the S-N gradient, others, such as the function of hunting or the species threatened, showed a clear separation between Africa and Europe.”

_______

Page 6

“Although most parks fell under the IUCN management category II (S1 Table), legal hunting for population control was more common in Europe (25 of 48 PAs, 52%) than Africa (3 of 66 PAs, 4.55%), with an average offtake of 389 ± 1189 animals/park (1.08 ± 1.72 animals/km2) in Europe and 17 ± 133 animals/park (0.04 ± 0.26 animals/km2 in Africa.”

Comment reviewer:

This is an interesting result, but lacking a lot of background; it this because there are differences in animal density? Or in the number of hunters? Or in the economic incentives to hunt? Or tradition? Europeans feed wildlife thus, densities can be huge for ungulates; hunters and gun ownership is much higher in Europe. None of these have to do with the number of threatened species or of hunting as a threat.

As presented, this is not very useful.

**Reply:** We mentioned this in the results section because we think it shows the differences in management. Unfortunately, our data does not allow for further investigate the reasons for this difference and we do not want the speculate in this section. However, we have listed a few possibilities for such a difference in the discussion section. In order to give a bit more background to these numbers, we have added on page 7:

“The maximum was reached in a PA in France, with on average, 7104 hunted wild boars (*Sus scrofa*) and 510 hunted deer per year (Fig. 1c, S21 Table).”

_______

Page 7

“This is even more pronounced for the category of “social function”. The “economic function” revealed a completely different pattern, with clearly decreasing hunting pressure with increasing HDI (Fig 2a, S4 Table).”

Comment reviewer:

yet, this is in contract with the results above about larger number of animals being hunted in Europe. How do you reconcile these results?

**Reply:** Thank you for this comment. We discuss this aspect in several parts of our discussion section, as this is indeed an interesting point. The higher number of animals being hunted in Europe are not evidence for the economic function, rather they are hunted (officially) for ecological reasons. It is also important to note that hunting is considered very differently on the continents, while hunting is frequently considered a management activity in European PAs, this is often not the case in African PAs.

_______

Page 7:

“Wildlife abundances were slightly negatively associated with high hunting threats (estimate = -0.76, CI = [-1.69, 0.06]).”

Comment reviewer:

this could be a spurious result from management practices: supplemental feeding in Europe leads to higher densities and thus lower perceived threat of hunting to populations.

**Reply:** We agree that this could be one additional explanation. Nevertheless, since this result is over all PAs, and most have no supplemented feeding practices, we assume that the most likely assumption might be that extensive hunting is leading to population declines. Nevertheless, it is of course important to mention that our results can also be attributed to other causes, since unfortunately we do not describe causalities but only correlations. Therefore, we have added a new paragraph in the discussion section where we point out this limitation. See "limitations of this study" (page 21).

_______

Page 7

Our control predictor of decreasing wildlife abundances which was measured over the past

ten years (S2 Table) was related to higher levels of HWC (estimate = -0.59, CI = [-1.57, 0.37]).

Comment reviewer:

Please rephrase; I do not understand what this means... lower animal abundance = higher HWC?

**Reply:** We modified this sentence so that it reads now (page 8)

‘Wildlife abundance trends over the last ten years, which were included as a control predictor in the analyses (Table S2), were negatively associated with higher levels of HWC (estimate = -0.59, CI = [-1.57, 0.37]).’

_______

Page 8

“Species were listed as threatened by illegal hunting (including all lethal interactions) 316 times, belonging to 117 different species (Fig. 3a).”

Comment reviewer:

what is the significance of these numbers? Its this a lot? Not a lot? How does it relate back to threat from hunting?

**Reply:** We agree that the figures given alone might not be very informative without the necessary context. However, park managers rarely know how many species are occurring in the parks, especially when it comes to rodents or elusive predators. Thus, no relation to the total number of species in each study site can be provided. Our purpose for mentioning these numbers was to give the reader a description of the prevalence of threat for a wide range of species. We modified the text to include an average number of threatened species by hunting per park.

We added now to the text on page 9:

“Unfortunately, it is not possible to give a proportional figure here, as the total number of species present in a PA was often unknown.”

_______

Page 8

“ (including all lethal interactions)”

Comment reviewer:

examples?

**Reply:** we have now expanded it to:

(Page 9)

“Species were listed as threatened by illegal hunting (including all lethal interactions, such as snaring, shooting, poisoning or beating to death) 316 times, belonging to 117 different species (Fig. 3a).

_______

Page 8

“Unfortunately, we received insufficient data for the comparison of the overall extracted biomass, legally or illegally hunted in Europe and Africa.”

Comment reviewer:

well, that would pretty much impossible anyway, since it is illegal!

**Reply:** Yes, this is true! We nevertheless thought it would be good to mention it here. However, we added now a reference to table S21, where we at least aimed at collecting some known quantities for a comparison. We futher changed the scentence to increase the meaning.

(Page 9)

“Although we unfortunately did not obtain sufficient data to compare the total biomass taken, legally or illegally hunted, in Europe and Africa, the limited data suggest a higher quantity taken in Africa (S21 Table).”

_______

Page 8

“A higher current threat to predators was associated with an increase in their abundance.”

Comment reviewer:

Not sure I follow this: predators increase in numbers, therefore their conservation status should change... Higher hunting reflect higher availability, but does it really threaten the population? This is quite circumstantial and not well backed up y the data presented here.

(Illegal hunting!)

**Reply:** We agree that this sentence might be misleading. The expansion of predator populations in Europe is associated with an increase in illegal hunting, as the recolonization areas host conflicts with interest groups.

To increase clarity, we reformulated the sentence now:

Page 9

“Predators were more likely listed as threatened by illegal hunting when their abundance increased over the last ten years (estimate = 0.57, CI = [-0.31, 1,49]).”

_______

Page 8

“Here, 63.1% of the species (130 of 206 cases, 56 distinct species) were threatened more when ranging outside borders, and 36.9% (76 of 206 cases, 45 distinct species) were threatened more within the park territory (Fig. 4a).”

Comment reviewer:

Please see comment above on "adjacent areas": what is "outside"? How can PA managers know and assess what happens outside the PAs?

**Reply:** According to our knowledge and experience, park managers have sufficient information about hunting in the vicinity of their PAs. Vicinity means up to several kilometers away from the park border and differs between species and PAs assessed. The question posed to the PA managers is to which degree species are threatened by illegal killings when ranging outside PA borders (see table S2).

We added now additional sentences to clarify this point:

On page 10:

“How far species range beyond borders is dependent on the ecology.”

On page 11:

Threats to animals when ranging outside PAs also declined, albeit to a lesser extent (Fig. 4c, S12 Table, HDI: estimate = -0.95, CI = [-1.94, -0.01].

On page 25:

“Our first two responses were a rating of how protected species from the park were affected by illegal hunting within administrative park borders or when ranging outside the park borders.”

On page 26:

“Which municipalities and in which area belong to the local communities was determined here by the park manager.”

_______

Page 8

“Threats also declined outside of Pas..”

Comment reviewer:

where? In Europe?

**Reply:** The decline we observed was both in Africa and Europe with increasing HDI values. In the plot, the transition from values representing African and European PAs, respectively, is marked with a vertical dashed line.

_______

Page 9

“The data distribution suggests that particularly the absence of the threat increased with HDI. Protection efforts, including ranger patrols, a permanent research station, and buffer zones, were weakly associated with a lower threat within PAs (estimate = -0.58, CI = [-1.51, 0.28]).”

Comment reviewer:

still talking about illegal hunting, correct? This is a good, yet, expected finding for Europe given the Habitats Directive.

**Reply:** Yes, this still refers to illegal hunting and we agree with the point you raised. We added now on page 11:

“The data distribution suggests that the absence of the threat from illegal hunting particularly increased with the HDI.”

_______

Page 9

“Supportive local community characteristics…”

Comment reviewer:

Elaborate on these.. what are these characteristics?

**Reply:** Thank you for this point. We now explained the composition of the variable here (page 11).

“Supportive local community characteristics, including mutual trust and conservation-friendly attitudes and culture, were linked to lower threats from illegal hunting to animals within (Fig. 5a, S11 Table, estimate = -0.99, CI = [-1.91, -0.12]) and outside of PAs (Fig. 5e, S12 Table, estimate = -0.89, CI = [-1.83, 0.01]).”

_______

Page 9

“Remarkably, in contrast to the effects of HDI, the effects here were less pronounced within rather than outside of parks.”

Comment reviewer:

Hmmm, maybe "expectedly"? This study deals with attitudes and social and economic context locally. HDI is such a gross metric, and does not necessarily have much to do with how educated local people are or their attitudes towards wildlife or the level of gun ownership. There are remarkable differences between neighboring countries in Europe on how countries deal with wildlife and hey are contrary to what you would expect. Take for example wolves: attitudes towards wolves are less negative in Eastern Europe (see Romania, Poland) compared to Western Europe (see France, Italy), yet HDI is the exact opposite.... Overall, HDI may be useful for global analyses, but parsing out and explaining local processes, as used here, is not straightforward.

I would rather expect that local predictor to tell a better story than he HDI... In fact, if the authors use HDI to justify the use of the 'gradient', they are probely better of not emphasizing it.

**Reply:** Thank you for these important insights, which we fully agree with. We have now modified the text so that it reads

(Page 11)

‘In comparison to the effects of HDI, these effects were less pronounced within than outside PAs.’

We further point out that community characteristics and population densities are “local”, while HDI is a national scale.

Page 26:

“We approximated the S-N gradient with the HDI (2017), a composite national index of life expectancy, education, and per capita income (gross national income GNI (PPP) per capita) [80]. We included multiple-scale predictors known to affect human–wildlife relationships. At the landscape scale, we included human population density (2015) [2]. At the local scale, we constructed an index for community characteristics based on questions regarding nature-culture relationships, attitudes towards nature, conservation, the concept of protected areas and their current management, and levels of mutual trust between communities and park management [25].”

_______

Page 10

“Although comparative studies are rare, hunting in the Global South is often portrayed as fundamentally different from hunting in the Global North [22].”

Comment reviewer:

Yes, it is COMPARATIVE STUDY, not a gradient of N-S or S-N

**Reply:** Thank you for your comment. We would like to refer to the details we have provided above on related comments and responses.

_______

Page 10

“The prevailing social function is remarkable because hunting in PAs is

considered an ecological necessity [10].”

Comment reviewer:

please use the work 'remarkable' sparingly... change to something that is not as hyperbolic

**Reply:** We replace ‘remarkable’ by ‘unexpected’

(page 11)

“The prevailing social function is unexpected because hunting in PAs is frequently presented as an ecological necessity”

_______

Page 10

“our results emphasize the call for an integrated European management policy [33].”

Comment reviewer:

Hmm... this is indeed a great suggestion, but I do not see how the results of this study lead to this conclusion (see my comments above).

**Reply:** We agree that our wording here was unclear and have now reworded it to:

(page 14)

**“**The prevailing social function is unexpected because hunting in PAs is frequently presented as an ecological necessity [9]. In addition, ecological factors, such as the presence of large predators, show little effect on offtake rates. Some parks, however, have adapted to the return of large predators, such as the Czech Šumava National Park, which declared no-hunting zones after the arrival of a wolf pack [27]. Nevertheless, the prevailing social function might confirm that current ungulate management in Europe is determined by the sociopolitical contexts rather than by ecological parameters or the guidelines of the International Union for Conservation of Nature (IUCN) [28].”

And added in section Regional and universal solutions (page 18):

“Additionally, as observed in our study, hunting in European PAs serves not only an ecological function but also a social function (Fig. 2a). This is disputable and emphasises the call for an integrated European management policy [28]. PA management needs to obtain the main authority over hunting activities in PAs to develop management plans in cooperation with hunters so that hunting activities fully serve the protected areas’ objectives.”

_______

Page 11

“Overall, the continental differences in the legal frameworks for hunting within the same IUCN management categories are striking and might be rooted in colonialism, where African hunters were stigmatized, criminalized and displaced from PAs [22,23] and the insufficient co-determination of local actors is still criticized by decolonization movements [19].”

Comment reviewer:

did same-category As in Europe and Africa have the same management objectives? If not, then this is a stretch and the differences could be rooted in a myriad of other factors not assessed here.

**Reply:** Thank you for this comment. Most parks fall into the same management category in our study.

We mention in page 6:

“Most PAs fell into IUCN management category II (69%; Africa 65%, Europe 72%), while the remaining PAs were in IUCN categories III-VI or had no assigned category (see S1 Table).”

We also changed the text on page 15 to:

“Potential explanations include colonial legacies, with African hunters being stigmatised, criminalised and displaced from PAs [18,19]. Furthermore, international organisations have been strongly involved in African PA management, while local actors have been insufficiently included [16] in establishing strong institutional structures regulating hunting..“

Also we added in “Limitations of this study” that interpretation are based on our suggestions and could, indeed, be rooted in other factors.

_______

Page 11

“like large herbivores such as the European bison (Bison bonasus), were hunted to local extinction,”

Comment reviewer:

whoa! When did this happen and does this relate to the current study, which uses hunt data from the last 10 years? European bison is subject to reintroduction in several countries in Europe, and not threatened by hunting... Please provide other, more meaningful examples.

**Reply:** To clarify, we have provided further explanation that bison have historically been exterminated by hunting on page 15:

” Moreover, several vulnerable species, including large herbivores, e.g., the European bison, have historically been hunted to (local) extinction.”

_______

Page 11

“….resilient species….”

Comment reviewer:

or not of economic interest!

**Reply:** We wrote now on page 15:

“In combination with a lack of economic interest for other species, this has resulted in impoverished but resilient species communities in Europe”].”

_______

Page 11

“The social function of hunting in the North (Fig. 2a) can encourage the persecution of predators if it is associated with enjoyment and status or competition over common prey [6,9].”

Comment reviewer:

yes, you nailed this... it also affects tolerance which is discussed above. Also, see paper by Chapron and Treves about wolves in US Midwest and how legal hunting encouraged poaching.

**Reply:** Thank you for your thoughts on this. We have included the paper by Chapron and Treves into the manuscript (page 15).

“Moreover, policy signals that allow, for example, the state-level culling of wolves can send negative messages about their conservation value and the acceptability of poaching, which can ultimately increase the poaching of these protected species [35].”

_______

Page 11

“Persecution does not yet seem to have decimated the overall predator populations.”

Comment reviewer:

you mean "it has not happened recently"... Persecution is responsible for most extirpations

**Reply:** Thank you for your comment, we agree that this needs to be formulated more clearly and have revised the sentence.

Page 16

“Our results suggest that illegal persecution is not severe enough to decimate the increasing predator populations, thereby confirming that predators are generally on the rise throughout Europe [4]. Nevertheless, single populations, such as lynxes (*Lynx lynx*) in the Bohemian Forest Ecosystem, are strongly endangered by illegal persecution [4,5]"

_______

Comment reviewer:

Currently by hunting? Are they strictly protected?

**Reply:** To clarify, this statement refers to illegal hunting, as we wrote now on page 16:

“Our results suggest that illegal persecution is not severe enough to decimate the increasing predator populations, thereby confirming that predators are generally on the rise throughout Europe [4]. Nevertheless, single populations, such as lynxes (*Lynx lynx*) in the Bohemian Forest Ecosystem, are strongly endangered by illegal persecution [4,5].”.

_______

Page 12

“Alternative livelihoods available when the HDI rises may outweigh the benefits of illegal and, thus, risky hunting inside PAs.”

Comment reviewer:

Also a MUCH STRONGER regulatory framework under the Habitats Directive, along with enforcement etc.

**Reply:** Even though this might be a valid argument, there are some points against it. For instance, highly armed ranger patrols are only common in parts of Africa and people are still taking the risk of poaching in parks. Also, regarding regulatory framework under the Habitats Directive, Europe lacks for example an integrated European management policy regarding culling, which considers ecological indicators (see for instance Van Beeck Calkoen et al, 2020). Given above, if possible, we retain our statement as it was in the text.

_______

Page 12

“European parks are even smaller, and their role is limited, as predators largely exist outside Pas..”

Comment reviewer:

Yes, I was wondering when you would bring this up... European parks cannot protect large carnivores and other large mammals; they may represent safe zones (for hibernating, pup and kitten rearing, calving, overwintering), but most species have HRs that are much larger than most European National Parks.

**Reply:** Thank you for your comments on this, we have incorporated the information you provided into the text.

We wrote now on page 16:

“They may represent safe zones (for hibernating, pup and kitten rearing, calving, wintering), but most carnivores and large herbivore species have home ranges that are much larger than typical European national parks.”

_______

Page 12

“…predators that range widely are most likely to disappear from small

reserves, irrespective of their population size [48].”

Comment reviewer:

This is likely highly dependent on the local context. A small PA surrounded by suburban or agriculture has a very different function than a PA embedded in a forest landscape. Please elaborate.

Reply: Indeed, this is a valuable point and would have been also our assumption. Surprisingly, Woodroffe & Ginsberg showed in the cited article that the edge effect is consistent across ten different taxa of predators and four different continents, including various regional conditions. (Woodroffe R, Ginsberg JR. Edge effects and the extinction of populations inside protected areas. Science (80- ). 1998;280: 2126–2128. doi:10.1126/science.280.5372.2126). If there are any novel insights which disprove this statement we are here open for further suggestions.

_______

Page 13

“Regarding what mitigates the threat to wildlife from illegal hunting inside and, in particular, outside parks, another identified commonality is the effect of supportive local community conditions. After conservation-friendly attitudes and cultures, mutual trust between management and communities showed the strongest effect (Fig. 5c,g). Trust has proven central to biodiversity conservation [52], such as to the success of PAs [53] or carnivore protection [54]. Trust can be broken when some feel that other parties' interests are being promoted at their expense [52]. This typically correlates with varying perceptions, uses, priorities, and impacts of wildlife or existing power gradients such as rural-urban or Global South-North [15,52,54]. Important trust-building mechanisms consist of transparent and fair decision-making processes and the ensured participation of all parties [52,55]. Notably, the inclusion of local communities in management contributed to supportive community conditions (Fig. 5i). Externally imposed rules can lead to animosity towards conservation or even forms of "resistance poaching" purposely targeting key conservation species [12,15]. Conversely, well-implemented inclusive approaches benefit the social, ecological, and ecological outcomes of PAs [53] by ensuring equity and trust, reinforcing positive attitudes, and considering the plurality of cultural values associated with nature [56].”

Comment reviewer:

-This is GREAT!-

**Reply:** Thanks!

_______

Page 13

Generalizable principles, universal pathways

Comment reviewer:

A bit grandiose, don't you think? Also vague.. pathways to what? Sustainable hunting? Low HWC?

**Reply:** As this section did not contain direct discussion of the findings, we have considerably changed it in the revised version of the manuscript and renamed it (Potential regional and universal solutions). Still, we felt that conclusions beyond our studied system is needed as this is one of the main reasons to conduct studies on such a large scale.

_______

Page 13

Comparisons against a contrasting background help synthesize knowledge by explaining differences and commonalities as well as universal mechanisms through conceptualizations against the backdrop of a comparative horizon [24,25].

Comment reviewer:

.. English for all, please

**Reply:** As we rewrote the entire section, this sentence has been removed.

_______

Page 14

“Therefore, the boundaries around the community of users and the resource itself must be clearly delineated [55,57].”

Comment reviewer:

Agreed, but Ostrom really focuses on local governance.

Wildlife management and hunting involves people that are not part of these 'boundaries' (think trophy hunters in Africa or Eastern Europe), thus this is a much more complex issue.

**Reply:** Thank you for this comment. This is exactly the point we wanted to make here, according to the Ostrom principles it cannot be a sustainable system, as it is much more complex. Even if we consider only the immediate neighborhood of a PA, the Ostrom principles are violated.

We write now on page 21

“Finally, parks are largely influenced by factors beyond the control of managers (i.e., HDI, population densities, threat beyond borders). This underscores a general shortcoming of the PA concept, as parks are not closed systems and parts of larger systems that require collaboration across multiple scales and levels [54,62]. Decision and policy makers must be made aware of the potential consequences of their actions for wildlife, even when those actions are apparently not directly linked to PAs.”

_______

Page 14

“Critically, some arrangements are poorly aligned. Northern countries struggled to protect predators (Fig. 3b), controlled their populations within PAs (i.e., Sweden) (S20 Table), and opposed rewilding while simultaneously advocating for coexistence abroad with costs disproportionately borne by the rural Global South [20]. Likewise, apparent continental

differences in legal settings (Fig. 1c, Fig. 2a, Fig. 3c) may still be relics of colonial times and indicate a lack of local participation in the South. Furthermore, exclusionary and militarized approaches are becoming normalized among some conservation agents and sub-Saharan African locations, seeding deep mistrust among residents [15,22]. These examples violate generalized principles, such as equal cost benefit sharing, self-determination, and inclusion, poisoning the collective efforts needed for wildlife protection [57].”

Comment reviewer:

yes, this is important and you should try to it directly to and discuss the actual findings of this work!

**Reply:** We think that this is an important point. We have adjusted this scentence to our actual results (page 21).

“Critically, some arrangements might violate core principles, such as equal cost–benefit sharing, self-determination, and inclusion, i.e., apparent continental differences in legal settings and the absence of social function of hunting in Africa, that might indicate lacking local participation in wildlife management and unequal costs and benefits from wildlife.”

_______

Page 15

“…regional axis…”

Comment reviewer:

????

**Reply:** These words are now removed from the text.

_______

Page 15

“Consensus is growing that only systemic changes in economics, values, and society can prevent biodiversity loss and future pandemics and address long-standing inequalities [56,63]. These challenges entail social and normative negotiation processes on every single organizational level.”

Comment reviewer:

Yes, very aspirational... we have been talking about this for decades... Likely to happen? Not really... we can't even agree over a vaccine or a mask...

Thus, I encourage the authors to think beyond this big picture, aspirational messages that have no future (at least in the short term, since the study addresses threatened species. What do we have available RIGHT NOW to act at the local level and implement the findings of this study? Think practically, not theoretically. Wildlife and wildlife managers do not care about theory (both social or ecological).

**Reply:** Thank you for pointing this out and sharing your perspective. We have now rewritten parts of the discussion and give now much more concrete and practical recommendations that can be implemented in the short term. Nevertheless, our study is a large-scale assessment and therefore, by its very design, more suitable for identifying universal mechanisms but less suitable for evaluating concrete local management strategies. Moreover, even if concrete quick solutions are important, systemic solutions are the only way out of the current biodiversity and climate crisis (e.g., IPCC 2022). The current developments make it important to think about long-term and more fundamental changes, as pointed out by the cited IPCC, IPBES reports or recently by the CBD (e.g., <https://www.theguardian.com/environment/2022/jan/19/expanding-national-parks-not-enough-to-protect-nature-say-scientists-aoe>). Our results show, that HDI is a main driver of unsustainable hunting and local conditions or conservation incentives could not outweigh this strong effect. We think it is important to emphasize here that if we will not improve conditions fundamentally and widespread, this situation will likely not change. Interestingly, good institutional setting, and fostering high trust levels within a society can help to make good decisions and to navigate challenging situations. For example, like your example on wearing masks, in so-called high-trust countries, such as in Denmark, the pandemic was better managed, thanks to the compliance of the citizens, than in lower-trust countries. (e.g., https://www.forbes.com/sites/lutzfinger/2021/05/06/get-vaccinated-how-trust-into-the-institutions-determines-vaccination-rates-in-the-eu/?sh=661350423a2f).However, we followed the suggestion and added much more concrete and short-term solutions to the text and fell that in considerably improved.

We made this point clearer by writing on page 22:

“Consensus is growing that only systemic changes in economics, values, and society can prevent biodiversity loss and address long-standing inequalities [51,67]. Protected areas and local communities cannot protect their wildlife until large-scale and sustainable economic development removes the economic constraints that currently drive poachers to exploit even remote parks. Wildlife will only be tolerated in human-dominated landscapes in the Global North when people accept that their rights mutually exist. These challenges entail social and normative negotiation processes at every single organisational level and the development of context-specific adaptive practical solutions and arrangements, which require strong institutional settings on different levels.”

_______

Page 18

“Predictors:”

Comment reviewer:

This section needs a good overhaul. Each of the predictors should be accompanied by arguments (backed by literature) of why they are applicable or relevant to this study, which will help authors formulate hypotheses that can be tested.

In this form, this looks like a shotgun approach, despite the carefully crafted Introduction.

**Reply:** Thank you for pointing this out (please see also related comments above). We have now extended Table S2 in the SI and provide for each predictor a hypothesis along with supporting literature.
